# Rapid detection of pathogenic fungi from coastal population with respiratory infections using microfluidic chip technology

**DOI:** 10.1186/s12879-024-09212-4

**Published:** 2024-03-18

**Authors:** Qingmei Yao, Yuan He, Liehua Deng, Dafeng Chen, Yuanli Zhang, Hui Luo, Wei Lei

**Affiliations:** 1 Guangdong Provincial Engineering Technology Research Center for Molecular Diagnosis and Innovative Drugs Translation of Cardiopulmonary Vascular Diseases, University Joint Laboratory of Guangdong Province and Macao Region on Molecular Targets and Intervention of Cardiovascular Diseases, Affiliated Hospital of Guangdong Medical University, Zhanjiang, 524001 China; 2 Department of Precision Laboratory, Affiliated Hospital of Guangdong Medical University, Zhanjiang, 524001 China; 3 Marine Biomedical Research Institution, Guangdong Provincial Laboratory of Southern Marine Science and Engineering, Guangdong Medical University, Zhanjiang, 524023 China; 4 Laboratory of Cardiovascular Diseases, Affiliated Hospital of Guangdong Medical University, Zhanjiang, 524001 China; 5 Department of Critical Care Medicine, Affiliated Hospital of Guangdong Medical University, Zhanjiang, 524001 China

**Keywords:** Pathogenic fungi, Respiratory infections, Microfluidic chip, Rapid detection, Diagnostic accuracy

## Abstract

**Background:**

Currently, culture methods are commonly used in clinical tests to detect pathogenic fungi including *Candida* spp. Nonetheless, these methods are cumbersome and time-consuming, thereby leading to considerable difficulties in diagnosis of pathogenic fungal infections, especially in situations that respiratory samples such as alveolar lavage fluid and pleural fluid contain extremely small amounts of microorganisms. The aim of this study was to elucidate the utility and practicality of microfluidic chip technology in quick detection of respiratory pathogenic fungi.

**Methods:**

DNAs of clinical samples (mainly derived from sputa, alveolar lavage fluid, and pleural fluid) from 64 coastal patients were quickly detected using microfluidic chip technology with 20 species of fungal spectrum and then validated by Real-time qPCR, and their clinical baseline data were analyzed.

**Results:**

Microfluidic chip results showed that 36 cases infected with *Candida* spp. and 27 cases tested negative for fungi, which was consistent with Real-time qPCR validation. In contrast, only 16 cases of fungal infections were detected by the culture method; however, one of the culture-positive samples tested negative by microfluidic chip and qPCR validation. Moreover, we found that the patients with *Candida* infections had significantly higher rates of platelet count reduction than fungi-negative controls. When compared with the patients infected with *C. albicans* alone, the proportion of males in the patients co-infected with multiple *Candidas* significantly increased, while their platelet counts significantly decreased.

**Conclusions:**

These findings suggest that constant temperature amplification-based microfluidic chip technology combined with routine blood tests can increase the detection speed and accuracy (including sensitivity and specificity) of identifying respiratory pathogenic fungi.

**Supplementary Information:**

The online version contains supplementary material available at 10.1186/s12879-024-09212-4.

## Background

The growth and reproduction of microorganisms require appropriate temperature and humidity. In particular, fungi prefer a humid and warm environment, and the optimum temperature for fungal growth is around 30 °C. Fungi are present everywhere in the environment such as atmosphere, plants, animals, feces, floors, and soil [1]. During spring and summer in Southern China, high-temperature and humidity environments are especially suitable for the growth and reproduction of fungi, and thereby increase risk of pathogenic fungal infections in population with long-term exposure to warm and humid environments. Older individuals and patients with chronic respiratory diseases are susceptible to respiratory fungal colonization and invasion [2]. Generally, fungal infections in the respiratory tract are dominated by *Candida* spp., especially *C. albicans* [3].

Detection methods such as 1,3-beta-D-glucan assay (G test) [4], microscopic examination [5], culture methods [6], histopathology [7], serology [8], and molecular biology [9] have been used in diagnosis of fungal infections. Traditional culture methods are widely used to characterize pathogenic fungi including *Candida* spp. However, culture-based methods are cumbersome, time-consuming, and thus unsuitable for rapid detection. Even more, the results are generally false negative. G test is used to detect one of the main structural components of the fungal cell wall (1,3-beta-D-glucan), but cannot distinguish fungal species. Despite the accuracy and efficacy of PCR-based methods [10], using a pair of primers or probes can only detect one type of the fungi; even multiplex PCR can detect no more than 5 *Candida* spp [11]. Therefore, bio-chips including microfluidic chip are becoming an alternative and revolutionary technology for assaying multiple fungi.

In this study, we aimed to rapidly detect the pathogenic fungi in coastal population with respiratory infectious diseases using microfluidic technology. To verify the accuracy of microfluidic chip, the results were further validated using Real-time qPCR and the clinical baseline data of the patients with or without fungal infections were analyzed.

## Methods

### Respiratory samples of patients

Patients with severe respiratory infections and hospitalized in the Department of Critical Care Medicine and Department of Respiratory and Critical Care Medicine between July 2020 and June 2021 at the Affiliated Hospital of Guangdong Medical University (Zhanjiang, China) were selected in this study. The patients had to be aged ≥ 18 years and their respiratory samples (sputum, alveolar lavage fluid, pleural fluid, etc.) were collected and sent to the department of precision laboratory for inspection. Cases who refused to accept the chip-based detection of pathogenic fungi were excluded. This study was approved by the ethics committee of the Affiliated Hospital of Guangdong Medical University (approval No. KT2022-119-01) and the consent of all patients was obtained.

### Clinical data collection

Clinical baseline data (including age, gender, fever, cough, sputum production, coma, use of antifungal agents, white blood cells, neutrophils, lymphocytes, red blood cells, platelets, glucose, total protein, albumin, and globulin) and microbiological culture results were obtained from electronic medical records (Table S1).

### Microfluidic-based detection of pathogenic fungi

The genomic DNAs of respiratory samples were isolated using the nucleic acid extraction kit (CapitalBio Technology, China) and subsequently used for chip-based detection of pathogenic fungi. The butterfly-shaped microfluidic chip system for rapid detection of broad-spectrum fungi was customized from CapitalBio Technology and prepared by constant temperature amplification with microfluidic technologies. The reaction of microfluidic-based detection was carried out on a microfluidic disk chip. Each chip had 24 reaction pools, in which were embedded with a set of specific primers targeting 20 species of pathogenic fungi. The fluorescent dye incorporation method was used, and the isothermal amplification technology adopted was loop-mediated isothermal amplification (LAMP). Its principle is based on the fact that DNA is in a dynamic equilibrium state at about 65℃. When any primer carries out base pairing extension to the complementary position of double-stranded DNA, the other strand will dissociate and become a single strand. After the one-step amplification and detection of target sequences, the samples generating snake-shaped amplification curves represented positive signals.

### Validation using real-time qPCR

According to the detection results of microfluidic chip, the specific primers of pathogenic *Candida* spp. including *C. albicans*, *C. tropicalis*, *C. glabrata*, *C. parapsilosis*, and *C. krusei* were synthesized for qPCR validation. Real-time qPCR was performed according to the specification of 2×RealStar Green Power Mixture with ROX (GenStar, China) by Applied Biosystems 7300 Real-Time PCR System (Thermo, USA). The cycling conditions were as follows: a pre-run at 95 °C for 10 min, 40 cycles of denaturation at 95 °C for 15 s, followed by a 60 °C annealing for 45 s and a 72 °C extension for 30 s; the melting curve conditions were automatically set up. The Real-time qPCR reactions were performed in triplicate and GAPDH was used as internal reference. Finally, the amplified products were electrophoresed on 1.0% agarose gels and developed using the nucleic acid dye Gelred (Biotium, USA). The sequences of PCR primers were listed in Table S2.

### Statistical analysis

The measurement data that conformed to a normal distribution were expressed as mean ± standard deviation (SD), and the *t*-test was used for the comparison between the two groups; the measurement data that did not conform to the normal distribution were expressed as median and interquartile range (IQR), and the comparison between the two groups was conducted by Mann-Whitney U test. The count data were expressed as the number of cases and percentage, and the difference was analyzed by chi-square test or Fisher’s exact value test. *P* < 0.05 was considered to demonstrate statistically significant differences.

## Results

### Fungal species detected by culture method and microfluidic chip technology

**Fig. 1:**
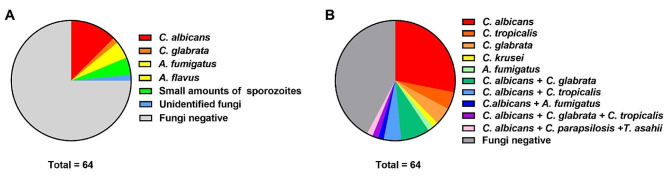
Detection of pathogenic fungi in patients with respiratory infections using the culture method and microfluidic chip. (**A**) Microbiological culture results. (**B**) Microfluidic chip analysis

A total of 64 patients with clinical infections were included in this study, and their detected results were finished by the microfluidic chip technologies (Fig. S1). Among the patients recruited, the mean age was 60.50 ± 16.44 years and 44 patients (68.75%) were male (Table S1). Only 8 cases with *C. albicans*, 1 case with *C. glabrata*, 2 cases with *A. fumigatus*, 1 case with *A. flavus*, 3 cases with small amounts of sporozoites, and 1 case with unidentified fungi were detected by culture method (Fig. 1A). In contrast, 36 cases with *Candida* spp. (including *C. albicans*, *C. tropicalis*, *C. glabrata*, *C. parapsilosis*, and *C. krusei*) and 1 case with *A. fumigatus* were detected using the microfluidic chip; and two of those cases infected with *Candida* spp. were co-infected with *T. asahii* and *A. fumigatus*, respectively (Fig. 1B). Confusingly, one of the *C. albicans*-positive samples detected by culture method was negative in the microfluidic chip. These findings imply that microfluidic-based chip system is a technique much more sensitive than traditional culture method.

### qPCR-based validation for chip detection

**Fig. 2:**
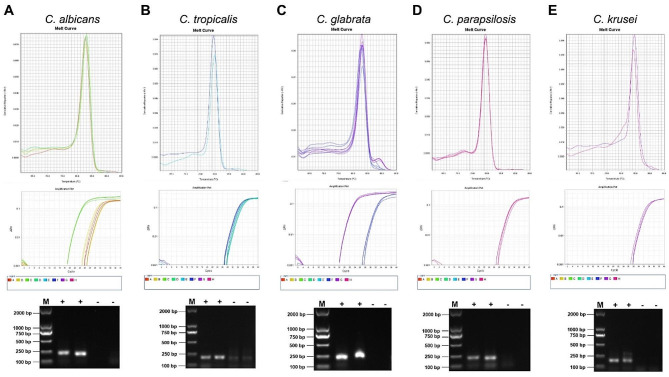
Real-time qPCR validation of *Candida* spp. Specific gene segments of *C. albicans* (**A**), *C. tropicalis* (**B**), *C. glabrata* (**C**), *C. parapsilosis* (**D**), and *C. krusei* (**E**) were amplified using Real-time qPCR and electrophoresed on 1.0% agarose gels. M, marker; +, *Candida* positive; -, *Candida* negative

Real-time qPCR was performed for validation, and the results were consistent with the chip detection results (Fig. 2). We found that the case with *C. albicans* positive in cultures but negative in the microfluidic chip showed no obvious amplification, and thus it was judged as negative. The qPCR validation indicated that microfluidic chip technology is more accurate in diagnosing fungal infections (Fig. S2).

### The accuracy for detection of *Candida* spp.

The patients were divided into case group (*Candida* infection; *n* = 36) and control group (fungal-negative; *n* = 27) according to the standard qPCR assay. As shown in Table 1, the sensitivity and specificity of microfluidic chip technology were both 100%, while the sensitivity of culture method was 22.22%. This finding suggests that the accuracy of microfluidic chip technology was superior to that of culture method.

**Table 1:** The sensitivity and specificity for detection of *Candida* spp.

		Microfluidic chip technology			Culture method	
Detection results		Case	Control		Case	Control
*Candida* positive		36	0		8	1
*Candida* negative		0	27		28	26
Sensitivity (%)		100.00			22.22	
Specificity (%)		100.00			96.30	

### Clinical features of patients infected with *Candida* spp

The clinical baseline data were compared between the patients infected with *Candida* spp. (*n* = 36) and fungal-negative controls (*n* = 27) detected by microfluidic chip. As shown in Table 2, age, gender, fever, cough, sputum production, coma, use of antifungal agents, and laboratory examination indexes (including white blood cells, neutrophils, red blood cells, platelets, blood glucose, total protein, albumin, and globulin) were not significantly different between the two groups, except for the proportion of abnormal platelet counts (*P* = 0.038). The results suggest that *Candida* infections may affect platelets.

**Table 2:** Clinical features of patients infected with *Candida* spp.

Variable	Patients infected with Candida spp. (*n* = 36)	Fungal-negative controls (*n* = 27)	*P* value
Age			
mean ± SD — years	63.03 ± 17.05	56.89 ± 15.48	0.141
18 ~ 60 years	17 (47.22)	18 (66.67)	0.200
> 60 years	19 (52.78)	9 (33.33)	
Gender — n (%)			
Female	11 (30.56)	8 (29.63)	1.000
Male	25 (69.44)	19 (70.37)	
Fever — n (%)	18	8	0.127
Cough — n (%)	12	9	1.000
Sputum production — n (%)	11	9	1.000
Coma	5	6	0.507
Abnormal Chest imaging — n (%)	34	27	0.502
Use of antifungal agents — n (%)	14	7	0.418
White blood cell count			
Median (IQR) — ×10^9^/L	11.65 (7.72 ~ 14.95)	10.31 (8.07 ~ 15.05)	0.934
> 10 × 10^9^/L — n (%)	21 (58.33)	15 (55.56)	0.758
< 4 × 10^9^/L — n (%)	1 (2.78)	2 (7.41)	
Neutrophil count			
Median (IQR) — ×10^9^/L	9.11 (5.92 ~ 11.78)	9.07 (6.15 ~ 14.37)	0.879
> 6.3 × 10^9^/L — n (%)	26 (72.22)	20 (74.07)	1.000
< 1.8 × 10^9^/L — n (%)	0 (0)	0 (0)	
Lymphocyte count			
Median (IQR) — ×10^9^/L	1.02 (0.71 ~ 1.27)	1.25 (0.67 ~ 1.7)	0.255
> 3.2 × 10^9^/L — n (%)	1 (2.78)	3 (11.11)	0.366
< 1.1 × 10^9^/L — n (%)	21 (58.33)	12 (44.44)	
Red blood cell count			
Median (IQR) — ×10^12^/L	2.83 (2.18 ~ 3.94)	2.93 (2.25 ~ 3.37)	0.950
> 5.0 (F) and 5.5 (M) ×10^12^/L	5 (13.89)	1 (3.7)	0.351
< 4.0 (F) and 4.5 (M) ×10^12^/L	30 (83.33)	24 (88.89)	
Platelet count			
Median (IQR) — ×10^9^/L	159.5 (113.5 ~ 228.75)	231 (114 ~ 382)	0.063
> 350 × 10^9^/L — n (%)	2 (5.56)	8 (29.63)	0.038
< 125 × 10^9^/L — n (%)	12 (33.33)	7 (25.93)	
Glucose			
Median (IQR) — mmol/L	7.15 (5.52 ~ 11.7)	7.93 (5.74 ~ 10.3)	0.972
> 6.1 mmol/L — n (%)	26 (72.22)	19 (70.37)	0.440
< 3.8 mmol/L — n (%)	4 (11.11)	1 (3.7)	
Total protein			
mean ± SD — g/L	57.61 ± 6.91	59.56 ± 8.07	0.320
> 85 g/L — n (%)	0 (0)	0 (0)	0.796
< 60 g/L — n (%)	23 (63.89)	16 (59.26)	
Albumin			
mean ± SD — g/L	30.2 ± 5.31	32.8 ± 5.26	0.059
> 53 g/L — n (%)	0 (0)	0 (0)	0.572
< 40 g/L — n (%)	35 (97.22)	25 (92.59)	
Globulin			
mean ± SD — g/L	27.13 ± 6.6	26.76 ± 5.71	0.812
> 32 g/L — n (%)	8 (22.22)	3 (11.11)	0.498
< 20 g/L — n (%)	5 (13.89)	3 (11.11)	

### Clinical features of patients co-infected with *C. albicans* and other *Candidas*

As shown in Table 3, when compared with the patients infected with *C. albicans* alone (*n* = 18), the proportion of males in the patients co-infected with *C. albicans* and other *Candidas* (*n* = 10) significantly increased (*P* = 0.048), while the platelet counts significantly decreased (*P* = 0.021). These findings indicate that *Candida* complex infections which are more likely to occur in males than females may lead to severe thrombocytopenia.

**Table 3:** Clinical features of patients infected with *C. albicans* and other *Candidas*

Variable	Patients infected with C. albicans alone (*n* = 18)	Patients co-infected with C. albicans and other Candidas (*n* = 10)	*P* value
Age			
mean ± SD — years	65.06 ± 15.67	61.2 ± 20.18	0.578
18 ~ 60 years	8 (44.44)	4 (40)	1.000
> 60 years	10 (55.56)	6 (60)	
Gender — n (%)			
Female	9 (50)	1 (10)	0.048
Male	9 (50)	9 (90)	
White blood cell count			
Median (IQR) — ×10^9^/L	11.75 (9.13 ~ 19.2)	12.12 (9.49 ~ 15.58)	1.000
> 10 × 10^9^/L — n (%)	11 (61.11)	7 (70)	0.703
< 4 × 10^9^/L — n (%)	0 (0)	0 (0)	
Neutrophil count			
Median (IQR) — ×10^9^/L	9.35 (6.8 ~ 16.4)	9.61 (7.09 ~ 12.37)	1.000
> 6.3 × 10^9^/L — n (%)	14 (77.78)	9 (90)	0.626
< 1.8 × 10^9^/L — n (%)	0 (0)	0 (0)	
Lymphocyte count			
mean ± SD — ×10^9^/L	1.13 ± 0.61	1.21 ± 0.72	0.752
> 3.2 × 10^9^/L — n (%)	1 (5.56)	0 (0)	0.204
< 1.1 × 10^9^/L — n (%)	10 (55.56)	5 (50)	
Red blood cell count			
Median (IQR) — ×10^12^/L	2.96 (2.25 ~ 4.36)	2.51 (1.87 ~ 2.97)	0.089
> 5.0 (F) and 5.5 (M) ×10^12^/L	3 (16.67)	0 (0)	0.365
< 4.0 (F) and 4.5 (M) ×10^12^/L	14 (77.78)	10 (100)	
Platelet count			
Median (IQR) — ×10^9^/L	220 (147 ~ 271.5)	119.5 (98.5 ~ 159.75)	0.021
> 350 × 10^9^/L — n (%)	2 (11.11)	0 (0)	0.054
< 125 × 10^9^/L — n (%)	3 (16.67)	6 (60)	
Glucose			
Median (IQR) — mmol/L	7.46 (6.14 ~ 12.19)	6.57 (5.26 ~ 8.72)	0.226
> 6.1 mmol/L — n (%)	14 (77.78)	7 (70)	0.814
< 3.8 mmol/L — n (%)	2 (11.11)	1 (10)	
Total protein			
mean ± SD — g/L	58.18 ± 8.3	55.44 ± 5.13	0.354
> 85 g/L — n (%)	0 (0)	0 (0)	0.247
< 60 g/L — n (%)	10 (55.56)	8 (80)	
Albumin			
mean ± SD — g/L	30.76 ± 6.2	29.77 ± 2.82	0.640
> 53 g/L — n (%)	0 (0)	0 (0)	1.000
< 40 g/L — n (%)	17 (94.44)	10 (100)	
Globulin			
mean ± SD — g/L	27.42 ± 7.36	25.67 ± 5.93	0.526
> 32 g/L — n (%)	5 (27.78)	2 (20)	1.000
< 20 g/L — n (%)	3 (16.67)	2 (20)	

## Discussion

While microfluidic chip has been used for rapid detection of fungal infections [12], it is still a bottleneck problem to test special clinical samples via high-throughput assays. To detect low-density fungi in body fluid, we customized the microfluidic-based chip system which allows timely detection of broad-spectrum pathogenic fungi, including *C. albicans*, *C. tropicalis*, *C. glabrata*, *C. parapsilosis*, *C. krusei*, *C. auris*, *A. fumigatus*, *A. flavus*, *A. niger*, *C. neoformans*, *T. asahii*, etc. It is shown that the pathogenic fungi detected by culture method and chip technology mainly consisted of *Candidas*, but the detection rate of microfluidic chip technology were higher than that of culture method (Fig. 1). The molecular detection results of *Candidas* using the microfluidic chip were validated by Real-time qPCR and showed good agreement (Fig. 2). Moreover, the blood routine examination implies that the platelets counts may be reduced in the patients with *Candida* infections and even associated with infection severity (Tables 2 and 3).

Invasive fungal infections are the most common causes of high mortality in ICU, and prompt antifungal therapy is a critical determinant for good clinical outcomes [13]. However, the treatment of fungal infections is often delayed due to the insensitivity and slow steps of microbial culture. Therefore, the development and validation of non-culture diagnostics targeting pathogenic fungi are of utmost priority in medicine. While commercial PCR detection has been widely applied to fungal examination, the use of DNA chip is becoming the method of choice for profiling fungal gene expression [14, 15]. The results of microbial culture showed only fungal infections in 16 of 64 patients, in which one was negative by microfluidic chip and qPCR validation (Fig. 1 and 2; Table 1). In contrast, 36 cases that test *Candida* positive by microfluidic chip were consistent with the qPCR validation (Figs. 1 and 2; Table 1), suggesting that the sensitivity and accuracy of microfluidic chip technology for fungal detection are more superior than culture methods. Furthermore, the microfluidic chip can simultaneously detect 20 species of pathogenic fungi, suggesting that it has the advantage of high-throughput detection compared with conventional PCR methods.

*Candida* spp. especially *C. albicans* play essential roles in invasive fungal infections. The development of diagnosis and treatment methods increases the risk of *Candida* infections [16–18], for instance, the use of broad-spectrum antibiotics, immunosuppressants, total parenteral nutrition, and mechanical ventilation leads to microbial dysbiosis. According to common sense, pathogenic microorganism infections cause blood-routine changes. However, we found that white blood cell counts were not significantly different between the patients infected with *Candida* spp. and fungal-negative controls (Table 2), probably due to additional bacterial infections.

As multiple players in innate immunity, platelets interact with *Candida* spp. and may contribute to the fatal outcome of invasive *Candida* infections [19, 20]. Thrombocytopenia, an important risk factor for fungal infections was also observed in the patients infected with *Candidas* (Table 3). It is suggested that *Candidas* enhance platelet adherence [19]. Platelets cannot directly reflect *Candida* infections, but may favor the survival of invasive *Candidas* in blood [21]. We found that platelet counts decreased in some patients with *Candida* infections in respiratory tract (Table 3), which is consistent with previous literature reports [22, 23], suggesting that *Candida* spp. may also be present in their blood and hence cause platelet aggregation and destruction. The mechanism by which platelets adhere to *Candidas* depends on the species of *Candidas* [20]. A study showed that *C. albicans* was able to evade complement-mediated killing without platelet adherence, and thus survived in blood during the infection period [24]. Moreover, it is showed that thrombocytopenia in the patients co-infected with *C. albicans* and other *Candidas* was more severe than those infected with *C. albicans* alone (Table 3). These findings suggest that platelet counts may be used as a parameter to investigate the severity of invasive *Candida* infections. However, the number of the patients in each group is small. Therefore, future studies should expand the sample size to observe and explore the relationship between platelets and *Candida* infections.

## Conclusions

In summary, microfluidic chip technology combined with routine blood tests can improve the accuracy and efficacy of detection of respiratory pathogenic *Candida* spp. However, the suitability of microfluidic chip technology for detecting clinically relevant fungal infections in other tissues and organs remains to be further explored.

### Electronic supplementary material

Below is the link to the electronic supplementary material.

Supplementary Material 1

Supplementary Material 2

Supplementary Material 3

Supplementary Material 4

## Data Availability

All data generated or analysed during this study are included in this published article [and its supplementary information files].
