# Supplementary material for: Rapid detection of pathogenic fungi from coastal population with respiratory infections using microfluidic chip technology
Source: BMC Infect Dis. 2024 Mar 18;24:326. doi: 10.1186/s12879-024-09212-4 (PMC10949588; doi:10.1186/s12879-024-09212-4)

Respiratory fungal nucleic acid test results

Affiliated Hospital Of Guangdong Medical University

Basic sample information

Name		Sexuality	male	Age	73 years old
Section	department of respiratory medicine	Ward		Bed Number	C19
ID		Clinical Diagnosis		Sample Types	sputum
Test Items		Chip Number	C030320238	Sample Number	c19

Detailed Results:

Serial Number	Test Items	Detected Results	Remark
1	Positive external control	+	
2	Positive internal control	+	
3	Candida albicans	+	
4	Candida tropicalis	-	
5	Candida parapsilosis	-	
6	Candida krusei	-	
7	Candida glabrata	-	
8	Candida auris	-	
9	Aspergillus fumigatus	-	
10	Aspergillus flavus	-	
11	Aspergillus niger	-	
12	Aspergillus terreus	-	
13	Aspergillus nidulans	-	
14	Aspergillus calidoustu	-	
15	Cryptococcus neoformans	-	
16	Cryptococcus gattii	-	
17	Mucor circinelloides	-	
18	Lichtheimia corymbifera	-	
19	Mucor pusillus	-	
20	Trichosporon asahii	-	
21	Pneumocystis jirovecii	-	
22	Rhizopus oryzae	-	
23	Negative control	-	
24	Blank	-	

Results Suggest:

Candida albicans index was positive.

Detector ____________ Auditor ____________ Detection date 2020-07-17 18:38:57

Respiratory fungal nucleic acid test results

Affiliated Hospital Of Guangdong Medical University

Basic sample information

Name		Sexuality	Male	Age	56 years old
Section	Second affiliated hospital	Ward		Bed Number	
ID		Clinical Diagnosis		Sample Types	bronchoalveolar lavage fluid
Test Items		Chip Number	C030320239	Sample Number	20200814001

Detailed Results:

Serial Number	Test Items	Detected Results	Remark
1	Positive external control	+	
2	Positive internal control	+	
3	Candida albicans	−	
4	Candida tropicalis	−	
5	Candida parapsilosis	−	
6	Candida krusei	−	
7	Candida glabrata	−	
8	Candida auris	−	
9	Aspergillus fumigatus	−	
10	Aspergillus flavus	−	
11	Aspergillus niger	−	
12	Aspergillus terreus	−	
13	Aspergillus nidulans	−	
14	Aspergillus calidoustu	−	
15	Cryptococcus neoformans	−	
16	Cryptococcus gattii	−	
17	Mucor circinelloides	−	
18	Lichtheimia corymbifera	−	
19	Mucor pusillus	−	
20	Trichosporon asahii	−	
21	Pneumocystis jirovecii	−	
22	Rhizopus oryzae	−	
23	Negative control	−	
24	Blank	−	

Results Suggest:

No abnormality was found after detection.

Detector ____________ Auditor ____________ Detection date 2020-08-14 16:32:53

Respiratory fungal nucleic acid test results

Affiliated Hospital Of Guangdong Medical University

Basic sample information

Name		Sexuality	Male	Age	74 years old
Section	ICU	Ward		Bed Number	5
ID		Clinical Diagnosis		Sample Types	sputum
Test Items		Chip Number	C030320241	Sample Number	20200821001

Detailed Results:

Serial Number	Test Items	Detected Results	Remark
1	Positive external control	+	
2	Positive internal control	+	
3	Candida albicans	−	
4	Candida tropicalis	+	
5	Candida parapsilosis	−	
6	Candida krusei	−	
7	Candida glabrata	−	
8	Candida auris	−	
9	Aspergillus fumigatus	−	
10	Aspergillus flavus	−	
11	Aspergillus niger	−	
12	Aspergillus terreus	−	
13	Aspergillus nidulans	−	
14	Aspergillus calidoustu	−	
15	Cryptococcus neoformans	−	
16	Cryptococcus gattii	−	
17	Mucor circinelloides	−	
18	Lichtheimia corymbifera	−	
19	Mucor pusillus	−	
20	Trichosporon asahii	−	
21	Pneumocystis jirovecii	−	
22	Rhizopus oryzae	−	
23	Negative control	−	
24	Blank	−	

Results Suggest:

Candida tropicalis index was positive.

Detector ____________ Auditor ____________ Detection date 2020-08-21 17:49:15

Respiratory fungal nucleic acid test results

Affiliated Hospital Of Guangdong Medical University

Basic sample information

Name		Sexuality	Male	Age	55 years old
Section	emergency ICU	Ward		Bed Number	7
ID		Clinical Diagnosis		Sample Types	sputum
Test Items		Chip Number	C030320242	Sample Number	20200821002

Detailed Results:

Serial Number	Test Items	Detected Results	Remark
1	Positive external control	+	
2	Positive internal control	+	
3	Candida albicans	−	
4	Candida tropicalis	+	
5	Candida parapsilosis	−	
6	Candida krusei	−	
7	Candida glabrata	−	
8	Candida auris	−	
9	Aspergillus fumigatus	−	
10	Aspergillus flavus	−	
11	Aspergillus niger	−	
12	Aspergillus terreus	−	
13	Aspergillus nidulans	−	
14	Aspergillus calidoustu	−	
15	Cryptococcus neoformans	−	
16	Cryptococcus gattii	−	
17	Mucor circinelloides	−	
18	Lichtheimia corymbifera	−	
19	Mucor pusillus	−	
20	Trichosporon asahii	−	
21	Pneumocystis jirovecii	−	
22	Rhizopus oryzae	−	
23	Negative control	−	
24	Blank	−	

Results Suggest:

Candida tropicalis index was positive.

Detector ____________ Auditor ____________ Detection date 2020-08-22 11:57:25

Respiratory fungal nucleic acid test results

Affiliated Hospital Of Guangdong Medical University

Basic sample information

Name		Sexuality	Male	Age	80 years old
Section	emergency ICU	Ward		Bed Number	9
ID		Clinical Diagnosis		Sample Types	sputum
Test Items		Chip Number	C030320243	Sample Number	20200821003

Detailed Results:

Serial Number	Test Items	Detected Results	Remark
1	Positive external control	+	
2	Positive internal control	+	
3	Candida albicans	+	
4	Candida tropicalis	-	
5	Candida parapsilosis	-	
6	Candida krusei	-	
7	Candida glabrata	-	
8	Candida auris	-	
9	Aspergillus fumigatus	-	
10	Aspergillus flavus	-	
11	Aspergillus niger	-	
12	Aspergillus terreus	-	
13	Aspergillus nidulans	-	
14	Aspergillus calidoustu	-	
15	Cryptococcus neoformans	-	
16	Cryptococcus gattii	-	
17	Mucor circinelloides	-	
18	Lichtheimia corymbifera	-	
19	Mucor pusillus	-	
20	Trichosporon asahii	-	
21	Pneumocystis jirovecii	-	
22	Rhizopus oryzae	-	
23	Negative control	-	
24	Blank	-	

Results Suggest:

Candida albicans index was positive.

Detector ____________ Auditor ____________ Detection date 2020-08-22 14:12:35

Respiratory fungal nucleic acid test results

Affiliated Hospital Of Guangdong Medical University

Basic sample information

Name		Sexuality	Male	Age	54 years old
Section	emergency ICU	Ward		Bed Number	204
ID		Clinical Diagnosis		Sample Types	sputum
Test Items		Chip Number	C030320244	Sample Number	20200821004

Detailed Results:

Serial Number	Test Items	Detected Results	Remark
1	Positive external control	+	
2	Positive internal control	+	
3	Candida albicans	+	
4	Candida tropicalis	−	
5	Candida parapsilosis	+	
6	Candida krusei	−	
7	Candida glabrata	−	
8	Candida auris	−	
9	Aspergillus fumigatus	−	
10	Aspergillus flavus	−	
11	Aspergillus niger	−	
12	Aspergillus terreus	−	
13	Aspergillus nidulans	−	
14	Aspergillus calidoustu	−	
15	Cryptococcus neoformans	−	
16	Cryptococcus gattii	−	
17	Mucor circinelloides	−	
18	Lichtheimia corymbifera	−	
19	Mucor pusillus	−	
20	Trichosporon asahii	+	
21	Pneumocystis jirovecii	−	
22	Rhizopus oryzae	−	
23	Negative control	−	
24	Blank	−	

Results Suggest:

Candida albicans,Candida parapsilosis and Trichosporon asahii indexes were positive.

Detector ____________ Auditor ____________ Detection date 2020-08-22 14:14:38

Respiratory fungal nucleic acid test results

Affiliated Hospital Of Guangdong Medical University

Basic sample information

Name		Sexuality	Male	Age	49 years old
Section	emergency ICU	Ward		Bed Number	208
ID		Clinical Diagnosis		Sample Types	sputum
Test Items		Chip Number	C030320240	Sample Number	20200821005

Detailed Results:

Serial Number	Test Items	Detected Results	Remark
1	Positive external control	+	
2	Positive internal control	+	
3	Candida albicans	+	
4	Candida tropicalis	-	
5	Candida parapsilosis	-	
6	Candida krusei	-	
7	Candida glabrata	-	
8	Candida auris	-	
9	Aspergillus fumigatus	-	
10	Aspergillus flavus	-	
11	Aspergillus niger	-	
12	Aspergillus terreus	-	
13	Aspergillus nidulans	-	
14	Aspergillus calidoustu	-	
15	Cryptococcus neoformans	-	
16	Cryptococcus gattii	-	
17	Mucor circinelloides	-	
18	Lichtheimia corymbifera	-	
19	Mucor pusillus	-	
20	Trichosporon asahii	-	
21	Pneumocystis jirovecii	-	
22	Rhizopus oryzae	-	
23	Negative control	-	
24	Blank	-	

Results Suggest:

Candida albicans index was positive.

Detector ____________ Auditor ____________ Detection date 2020-08-21 18:48:31

Respiratory fungal nucleic acid test results

Affiliated Hospital Of Guangdong Medical University

Basic sample information

Name		Sexuality	Male	Age	58 years old
Section	ICU	Ward		Bed Number	
ID		Clinical Diagnosis		Sample Types	sputum
Test Items		Chip Number	C030320245	Sample Number	20201030001

Detailed Results:

Serial Number	Test Items	Detected Results	Remark
1	Positive external control	+	
2	Positive internal control	+	
3	Candida albicans	+	
4	Candida tropicalis	–	
5	Candida parapsilosis	–	
6	Candida krusei	–	
7	Candida glabrata	–	
8	Candida auris	–	
9	Aspergillus fumigatus	–	
10	Aspergillus flavus	–	
11	Aspergillus niger	–	
12	Aspergillus terreus	–	
13	Aspergillus nidulans	–	
14	Aspergillus calidoustu	–	
15	Cryptococcus neoformans	–	
16	Cryptococcus gattii	–	
17	Histoplasma capsulatum	–	
18	Lichtheimia corymbifera	–	
19	Mucor pusillus	–	
20	Trichosporon asahii	–	
21	Pneumocystis jirovecii	–	
22	Rhizopus oryzae	–	
23	Negative control	–	
24	Blank	–	

Results Suggest:

Candida albicans index was positive.

Detector ____________ Auditor ____________ Detection date 2023-06-25 20:44:45

Respiratory fungal nucleic acid test results

Affiliated Hospital Of Guangdong Medical University

Basic sample information

Name		Sexuality	Male	Age	89 years old
Section	ICU	Ward		Bed Number	
ID		Clinical Diagnosis		Sample Types	sputum
Test Items		Chip Number	C030320246	Sample Number	20201030002

Detailed Results:

Serial Number	Test Items	Detected Results	Remark
1	Positive external control	+	
2	Positive internal control	+	
3	Candida albicans	-	
4	Candida tropicalis	-	
5	Candida parapsilosis	-	
6	Candida krusei	-	
7	Candida glabrata	-	
8	Candida auris	-	
9	Aspergillus fumigatus	-	
10	Aspergillus flavus	-	
11	Aspergillus niger	-	
12	Aspergillus terreus	-	
13	Aspergillus nidulans	-	
14	Aspergillus calidoustu	-	
15	Cryptococcus neoformans	-	
16	Cryptococcus gattii	-	
17	Histoplasma capsulatum	-	
18	Lichtheimia corymbifera	-	
19	Mucor pusillus	-	
20	Trichosporon asahii	-	
21	Pneumocystis jirovecii	-	
22	Rhizopus oryzae	-	
23	Negative control	-	
24	Blank	-	

Results Suggest:

No abnormality was found after detection.

Detector ____________ Auditor ____________ Detection date 2020-10-30 16:59:25

Respiratory fungal nucleic acid test results

Affiliated Hospital Of Guangdong medical university

Basic sample information

Name		Sexuality	Female	Age	83 years old
Section	ICU	Ward		Bed Number	
ID		Clinical Diagnosis		Sample Types	sputum
Test Items		Chip Number	C030320248	Sample Number	20201030003

Detailed Results:

Serial Number	Test Items	Detected Results	Remark
1	Positive external control	+	
2	Positive internal control	+	
3	Candida albicans	+	
4	Candida tropicalis	–	
5	Candida parapsilosis	–	
6	Candida krusei	–	
7	Candida glabrata	–	
8	Candida auris	–	
9	Aspergillus fumigatus	–	
10	Aspergillus flavus	–	
11	Aspergillus niger	–	
12	Aspergillus terreus	–	
13	Aspergillus nidulans	–	
14	Aspergillus calidoustu	–	
15	Cryptococcus neoformans	–	
16	Cryptococcus gattii	–	
17	Histoplasma capsulatum	–	
18	Lichtheimia corymbifera	–	
19	Mucor pusillus	–	
20	Trichosporon asahii	–	
21	Pneumocystis jirovecii	–	
22	Rhizopus oryzae	–	
23	Negative control	–	
24	Blank	–	

Results Suggest:

Candida albicans index was positive.

Detector ____________ Auditor ____________ Detection date 2020-10-30 17:53:45

Respiratory fungal nucleic acid test results

Affiliated Hospital Of Guangdong medical university

Basic sample information

Name		Sexuality	Male	Age	80 years old
Section	ICU	Ward		Bed Number	
ID		Clinical Diagnosis		Sample Types	sputum
Test Items		Chip Number	C030320249	Sample Number	20201209001

Detailed Results:

Serial Number	Test Items	Detected Results	Remark
1	Positive external control	+	
2	Positive internal control	+	
3	Candida albicans	-	
4	Candida tropicalis	+	
5	Candida parapsilosis	-	
6	Candida krusei	-	
7	Candida glabrata	-	
8	Candida auris	-	
9	Aspergillus fumigatus	-	
10	Aspergillus flavus	-	
11	Aspergillus niger	-	
12	Aspergillus terreus	-	
13	Aspergillus nidulans	-	
14	Aspergillus calidoustu	-	
15	Cryptococcus neoformans	-	
16	Cryptococcus gattii	-	
17	Histoplasma capsulatum	-	
18	Lichtheimia corymbifera	-	
19	Mucor pusillus	-	
20	Trichosporon asahii	-	
21	Pneumocystis jirovecii	-	
22	Rhizopus oryzae	-	
23	Negative control	-	
24	Blank	-	

Results Suggest:

Candida tropicalis index was positive.

Detector ____________ Auditor ____________ Detection date 2020-12-09 17:46:27

Respiratory fungal nucleic acid test results

Affiliated Hospital Of Guangdong medical university

Basic sample information

Name		Sexuality	Male	Age	5 years old
Section	ICU	Ward		Bed Number	204
ID		Clinical Diagnosis		Sample Types	sputum
Test Items		Chip Number	C020420068	Sample Number	20201209002

Detailed Results:

Serial Number	Test Items	Detected Results	Remark
1	Positive external control	+	
2	Positive internal control	+	
3	Candida albicans	-	
4	Candida tropicalis	-	
5	Candida parapsilosis	-	
6	Candida krusei	-	
7	Candida glabrata	-	
8	Candida auris	-	
9	Aspergillus fumigatus	-	
10	Aspergillus flavus	-	
11	Aspergillus niger	-	
12	Aspergillus terreus	-	
13	Aspergillus nidulans	-	
14	Aspergillus calidoustu	-	
15	Cryptococcus neoformans	-	
16	Cryptococcus gattii	-	
17	Histoplasma capsulatum	-	
18	Lichtheimia corymbifera	-	
19	Mucor pusillus	-	
20	Trichosporon asahii	-	
21	Pneumocystis jirovecii	-	
22	Rhizopus oryzae	-	
23	Negative control	-	
24	Blank	-	

Results Suggest:

No abnormality was found after detection.

Detector ____________ Auditor ____________ Detection date 2020-12-10 17:12:55

Respiratory fungal nucleic acid test results

Affiliated Hospital Of Guangdong medical university

Basic sample information

Name		Sexuality	Male	Age	87 years old
Section	ICU	Ward		Bed Number	208
ID		Clinical Diagnosis		Sample Types	sputum
Test Items		Chip Number	C020420069	Sample Number	20201209003

Detailed Results:

Serial Number	Test Items	Detected Results	Remark
1	Positive external control	+	
2	Positive internal control	+	
3	Candida albicans	-	
4	Candida tropicalis	-	
5	Candida parapsilosis	-	
6	Candida krusei	-	
7	Candida glabrata	-	
8	Candida auris	-	
9	Aspergillus fumigatus	-	
10	Aspergillus flavus	-	
11	Aspergillus niger	-	
12	Aspergillus terreus	-	
13	Aspergillus nidulans	-	
14	Aspergillus calidoustu	-	
15	Cryptococcus neoformans	-	
16	Cryptococcus gattii	-	
17	Histoplasma capsulatum	-	
18	Lichtheimia corymbifera	-	
19	Mucor pusillus	-	
20	Trichosporon asahii	-	
21	Pneumocystis jirovecii	-	
22	Rhizopus oryzae	-	
23	Negative control	-	
24	Blank	-	

Results Suggest:

No abnormality was found after detection.

Detector ____________ Auditor ____________ Detection date 2020-12-11 09:13:38

Respiratory fungal nucleic acid test results

Affiliated Hospital Of Guangdong medical university

Basic sample information

Name		Sexuality	Male	Age	58 years old
Section	ICU	Ward		Bed Number	305
ID		Clinical Diagnosis		Sample Types	sputum
Test Items		Chip Number	C020420072	Sample Number	20201209005

Detailed Results:

Serial Number	Test Items	Detected Results	Remark
1	Positive external control	+	
2	Positive internal control	+	
3	Candida albicans	-	
4	Candida tropicalis	-	
5	Candida parapsilosis	-	
6	Candida krusei	+	
7	Candida glabrata	-	
8	Candida auris	-	
9	Aspergillus fumigatus	-	
10	Aspergillus flavus	-	
11	Aspergillus niger	-	
12	Aspergillus terreus	-	
13	Aspergillus nidulans	-	
14	Aspergillus calidoustu	-	
15	Cryptococcus neoformans	-	
16	Cryptococcus gattii	-	
17	Histoplasma capsulatum	-	
18	Lichtheimia corymbifera	-	
19	Mucor pusillus	-	
20	Trichosporon asahii	-	
21	Pneumocystis jirovecii	-	
22	Rhizopus oryzae	-	
23	Negative control	-	
24	Blank	-	

Results Suggest:

Candida krusei index was positive.

Detector ____________ Auditor ____________ Detection date 2020-12-14 17:50:44

Respiratory fungal nucleic acid test results

Affiliated Hospital Of Guangdong medical university

Basic sample information

Name		Sexuality	Male	Age	42 years old
Section	ICU	Ward		Bed Number	311
ID		Clinical Diagnosis		Sample Types	sputum
Test Items		Chip Number	C020420071	Sample Number	20201209004

Detailed Results:

Serial Number	Test Items	Detected Results	Remark
1	Positive external control	+	
2	Positive internal control	+	
3	Candida albicans	-	
4	Candida tropicalis	-	
5	Candida parapsilosis	-	
6	Candida krusei	-	
7	Candida glabrata	-	
8	Candida auris	-	
9	Aspergillus fumigatus	-	
10	Aspergillus flavus	-	
11	Aspergillus niger	-	
12	Aspergillus terreus	-	
13	Aspergillus nidulans	-	
14	Aspergillus calidoustu	-	
15	Cryptococcus neoformans	-	
16	Cryptococcus gattii	-	
17	Histoplasma capsulatum	-	
18	Lichtheimia corymbifera	-	
19	Mucor pusillus	-	
20	Trichosporon asahii	-	
21	Pneumocystis jirovecii	-	
22	Rhizopus oryzae	-	
23	Negative control	-	
24	Blank	-	

Results Suggest:

No abnormality was found after detection.

Detector ________ Auditor ________ Detection date 2020-12-14 16:54:38

Respiratory fungal nucleic acid test results

Affiliated Hospital Of Guangdong medical university

Basic sample information

Name		Sexuality	Male	Age	57 years old
Section	ICU	Ward		Bed Number	
ID		Clinical Diagnosis		Sample Types	sputum
Test Items		Chip Number	C020420067	Sample Number	20201210001

Detailed Results:

Serial Number	Test Items	Detected Results	Remark
1	Positive external control	+	
2	Positive internal control	+	
3	Candida albicans	-	
4	Candida tropicalis	-	
5	Candida parapsilosis	-	
6	Candida krusei	-	
7	Candida glabrata	-	
8	Candida auris	-	
9	Aspergillus fumigatus	-	
10	Aspergillus flavus	-	
11	Aspergillus niger	-	
12	Aspergillus terreus	-	
13	Aspergillus nidulans	-	
14	Aspergillus calidoustu	-	
15	Cryptococcus neoformans	-	
16	Cryptococcus gattii	-	
17	Histoplasma capsulatum	-	
18	Lichtheimia corymbifera	-	
19	Mucor pusillus	-	
20	Trichosporon asahii	-	
21	Pneumocystis jirovecii	-	
22	Rhizopus oryzae	-	
23	Negative control	-	
24	Blank	-	

Results Suggest:

No abnormality was found after detection.

Detector ____________ Auditor ____________ Detection date 2020-12-10 16:10:19

Respiratory fungal nucleic acid test results

Affiliated Hospital Of Guangdong medical university

Basic sample information

Name		Sexuality	Female	Age	92 years old
Section	ICU	Ward		Bed Number	105
ID		Clinical Diagnosis		Sample Types	bronchoalveolar lavage fluid
Test Items		Chip Number	C020420073	Sample Number	2021012002

Detailed Results:

Serial Number	Test Items	Detected Results	Remark
1	Positive external control	+	
2	Positive internal control	+	
3	Candida albicans	+	
4	Candida tropicalis	-	
5	Candida parapsilosis	-	
6	Candida krusei	-	
7	Candida glabrata	-	
8	Candida auris	-	
9	Aspergillus fumigatus	-	
10	Aspergillus flavus	-	
11	Aspergillus niger	-	
12	Aspergillus terreus	-	
13	Aspergillus nidulans	-	
14	Aspergillus calidoustu	-	
15	Cryptococcus neoformans	-	
16	Cryptococcus gattii	-	
17	Histoplasma capsulatum	-	
18	Lichtheimia corymbifera	-	
19	Mucor pusillus	-	
20	Trichosporon asahii	-	
21	Pneumocystis jirovecii	-	
22	Penicillium marneffei	-	
23	Negative control	-	
24	Blank	-	

Results Suggest:

Candida albicans index was positive.

Detector ____________ Auditor ____________ Detection date 2021-01-20 16:49:56

Respiratory fungal nucleic acid test results

Affiliated Hospital Of Guangdong medical university

Basic sample information

Name		Sexuality	Female	Age	84 years old
Section	ICU	Ward		Bed Number	106
ID		Clinical Diagnosis		Sample Types	bronchoalveolar lavage fluid
Test Items		Chip Number	C020420074	Sample Number	20210120003

Detailed Results:

Serial Number	Test Items	Detected Results	Remark
1	Positive external control	+	
2	Positive internal control	+	
3	Candida albicans	+	
4	Candida tropicalis	-	
5	Candida parapsilosis	-	
6	Candida krusei	-	
7	Candida glabrata	-	
8	Candida auris	-	
9	Aspergillus fumigatus	-	
10	Aspergillus flavus	-	
11	Aspergillus niger	-	
12	Aspergillus terreus	-	
13	Aspergillus nidulans	-	
14	Aspergillus calidoustu	-	
15	Cryptococcus neoformans	-	
16	Cryptococcus gattii	-	
17	Histoplasma capsulatum	-	
18	Lichtheimia corymbifera	-	
19	Mucor pusillus	-	
20	Trichosporon asahii	-	
21	Pneumocystis jirovecii	-	
22	Penicillium marneffei	-	
23	Negative control	-	
24	Blank	-	

Results Suggest:

Candida albicans index was positive.

Detector ____________ Auditor ____________ Detection date 2021-01-20 17:45:21

Respiratory fungal nucleic acid test results

Affiliated Hospital Of Guangdong medical university

Basic sample information

Name		Sexuality	Female	Age	79 years old
Section	ICU	Ward		Bed Number	203
ID		Clinical Diagnosis		Sample Types	sputum
Test Items		Chip Number	C020420075	Sample Number	2021012104

Detailed Results:

Serial Number	Test Items	Detected Results	Remark
1	Positive external control	+	
2	Positive internal control	+	
3	Candida albicans	-	
4	Candida tropicalis	-	
5	Candida parapsilosis	-	
6	Candida krusei	-	
7	Candida glabrata	-	
8	Candida auris	-	
9	Aspergillus fumigatus	-	
10	Aspergillus flavus	-	
11	Aspergillus niger	-	
12	Aspergillus terreus	-	
13	Aspergillus nidulans	-	
14	Aspergillus calidoustu	-	
15	Cryptococcus neoformans	-	
16	Cryptococcus gattii	-	
17	Histoplasma capsulatum	-	
18	Lichtheimia corymbifera	-	
19	Mucor pusillus	-	
20	Trichosporon asahii	-	
21	Pneumocystis jirovecii	-	
22	Penicillium marneffei	-	
23	Negative control	-	
24	Blank	-	

Results Suggest:

No abnormality was found after detection.

Detector ____________ Auditor ____________ Detection date 2021-01-21 08:58:59

Respiratory fungal nucleic acid test results

Affiliated Hospital Of Guangdong medical university

Basic sample information

Name		Sexuality	Male	Age	73 years old
Section	ICU	Ward		Bed Number	207
ID		Clinical Diagnosis		Sample Types	sputum
Test Items		Chip Number	C020420076	Sample Number	2021012005

Detailed Results:

Serial Number	Test Items	Detected Results	Remark
1	Positive external control	+	
2	Positive internal control	+	
3	Candida albicans	+	
4	Candida tropicalis	−	
5	Candida parapsilosis	−	
6	Candida krusei	−	
7	Candida glabrata	−	
8	Candida auris	−	
9	Aspergillus fumigatus	−	
10	Aspergillus flavus	−	
11	Aspergillus niger	−	
12	Aspergillus terreus	−	
13	Aspergillus nidulans	−	
14	Aspergillus calidoustu	−	
15	Cryptococcus neoformans	−	
16	Cryptococcus gattii	−	
17	Histoplasma capsulatum	−	
18	Lichtheimia corymbifera	−	
19	Mucor pusillus	−	
20	Trichosporon asahii	−	
21	Pneumocystis jirovecii	−	
22	Penicillium marneffei	−	
23	Negative control	−	
24	Blank	−	

Results Suggest:

Candida albicans index was positive.

Detector ____________ Auditor ____________ Detection date 2021-01-21 09:57:23

Respiratory fungal nucleic acid test results

Affiliated Hospital Of Guangdong medical university

Basic sample information

Name		Sexuality	Female	Age	43 years old
Section	ICU	Ward		Bed Number	301
ID		Clinical Diagnosis		Sample Types	sputum
Test Items		Chip Number	C020420161	Sample Number	2021012006

Detailed Results:

Serial Number	Test Items	Detected Results	Remark
1	Positive external control	+	
2	Positive internal control	+	
3	Candida albicans	+	
4	Candida tropicalis	-	
5	Candida parapsilosis	-	
6	Candida krusei	-	
7	Candida glabrata	-	
8	Candida auris	-	
9	Aspergillus fumigatus	-	
10	Aspergillus flavus	-	
11	Aspergillus niger	-	
12	Aspergillus terreus	-	
13	Aspergillus nidulans	-	
14	Aspergillus calidoustu	-	
15	Cryptococcus neoformans	-	
16	Cryptococcus gattii	-	
17	Histoplasma capsulatum	-	
18	Lichtheimia corymbifera	-	
19	Mucor pusillus	-	
20	Trichosporon asahii	-	
21	Pneumocystis jirovecii	-	
22	Penicillium marneffei	-	
23	Negative control	-	
24	Blank	-	

Results Suggest:

Candida albicans index was positive.

Detector ____________ Auditor ____________ Detection date 2023-06-25 20:54:28

Respiratory fungal nucleic acid test results

Affiliated Hospital Of Guangdong medical university

Basic sample information

Name		Sexuality	Female	Age	48 years old
Section	ICU	Ward		Bed Number	J050
ID		Clinical Diagnosis		Sample Types	bronchoalveolar lavage fluid
Test Items		Chip Number	C010420162		2021012001

Detailed Results:

Serial Number	Test Items	Detected Results	Remark
1	Positive external control	+	
2	Positive internal control	+	
3	Candida albicans	–	
4	Candida tropicalis	–	
5	Candida parapsilosis	–	
6	Candida krusei	–	
7	Candida glabrata	–	
8	Candida auris	–	
9	Aspergillus fumigatus	–	
10	Aspergillus flavus	–	
11	Aspergillus niger	–	
12	Aspergillus terreus	–	
13	Aspergillus nidulans	–	
14	Aspergillus calidoustu	–	
15	Cryptococcus neoformans	–	
16	Cryptococcus gattii	–	
17	Histoplasma capsulatum	–	
18	Lichtheimia corymbifera	–	
19	Mucor pusillus	–	
20	Trichosporon asahii	–	
21	Pneumocystis jirovecii	–	
22	Penicillium marneffei	–	
23	Negative control	–	
24	Blank	–	

Results Suggest:

No abnormality was found after detection.

Detector ____________ Auditor ____________ Detection date 2021-01-21 12:11:58

Respiratory fungal nucleic acid test results

Affiliated Hospital Of Guangdong medical university

Basic sample information

Name		Sexuality	Male	Age	61 years old
Section	ICU	Ward		Bed Number	3001
ID		Clinical Diagnosis		Sample Types	sputum
Test Items		Chip Number	C010420163	Sample Number	2021012101

Detailed Results:

Serial Number	Test Items	Detected Results	Remark
1	Positive external control	+	
2	Positive internal control	+	
3	Candida albicans	-	
4	Candida tropicalis	-	
5	Candida parapsilosis	-	
6	Candida krusei	-	
7	Candida glabrata	-	
8	Candida auris	-	
9	Aspergillus fumigatus	-	
10	Aspergillus flavus	-	
11	Aspergillus niger	-	
12	Aspergillus terreus	-	
13	Aspergillus nidulans	-	
14	Aspergillus calidoustu	-	
15	Cryptococcus neoformans	-	
16	Cryptococcus gattii	-	
17	Histoplasma capsulatum	-	
18	Lichtheimia corymbifera	-	
19	Mucor pusillus	-	
20	Trichosporon asahii	-	
21	Pneumocystis jirovecii	-	
22	Penicillium marneffei	-	
23	Negative control	-	
24	Blank	-	

Results Suggest:

No abnormality was found after detection.

Detector ____________ Auditor ____________ Detection date 2021-01-21 17:27:09

Respiratory fungal nucleic acid test results

Affiliated Hospital Of Guangdong medical university

Basic sample information

Name		Sexuality	Female	Age	70 years old
Section	ICU	Ward		Bed Number	
ID		Clinical Diagnosis		Sample Types	sputum
Test Items		Chip Number	ZJ-202011010228	Sample Number	2021012601

Detailed Results:

Serial Number	Test Items	Detected Results	Remark
1	Positive external control	+	
2	Positive internal control	+	
3	Candida albicans	+	
4	Candida tropicalis	-	
5	Candida parapsilosis	-	
6	Candida krusei	-	
7	Candida glabrata	-	
8	Candida auris	-	
9	Aspergillus fumigatus	-	
10	Aspergillus flavus	-	
11	Aspergillus niger	-	
12	Aspergillus terreus	-	
13	Aspergillus nidulans	-	
14	Aspergillus calidoustu	-	
15	Cryptococcus neoformans	-	
16	Cryptococcus gattii	-	
17	Histoplasma capsulatum	-	
18	Lichtheimia corymbifera	-	
19	Mucor pusillus	-	
20	Trichosporon asahii	-	
21	Pneumocystis jirovecii	-	
22	Penicillium marneffei	-	
23	Negative control	-	
24	Blank	-	

Results Suggest:

Candida albicans index was positive.

Detector ____________ Auditor ____________ Detection date 2021-02-02 18:07:31

Respiratory fungal nucleic acid test results

Affiliated Hospital Of Guangdong medical university

Basic sample information

Name		Sexuality	Male	Age	89 years old
Section	ICU	Ward		Bed Number	3008
ID		Clinical Diagnosis		Sample Types	sputum
Test Items		Chip Number	c010420167	Sample Number	2021012102

Detailed Results:

Serial Number	Test Items	Detected Results	Remark
1	Positive external control	+	
2	Positive internal control	+	
3	Candida albicans	+	
4	Candida tropicalis	-	
5	Candida parapsilosis	-	
6	Candida krusei	-	
7	Candida glabrata	+	
8	Candida auris	-	
9	Aspergillus fumigatus	-	
10	Aspergillus flavus	-	
11	Aspergillus niger	-	
12	Aspergillus terreus	-	
13	Aspergillus nidulans	-	
14	Aspergillus calidoustu	-	
15	Cryptococcus neoformans	-	
16	Cryptococcus gattii	-	
17	Histoplasma capsulatum	-	
18	Lichtheimia corymbifera	-	
19	Mucor pusillus	-	
20	Trichosporon asahii	-	
21	Pneumocystis jirovecii	-	
22	Penicillium marneffei	-	
23	Negative control	-	
24	Blank	-	

Results Suggest:

Candida albicans and Candida glabrata indexes were positive.

Detector ____________ Auditor ____________ Detection date 2021-01-30 18:52:31

Respiratory fungal nucleic acid test results

Affiliated Hospital Of Guangdong medical university

Basic sample information

Name		Sexuality	Male	Age	48 years old
Section	ICU	Ward		Bed Number	3009
ID		Clinical Diagnosis		Sample Types	sputum
Test Items		Chip Number	c010420168	Sample Number	2021012103

Detailed Results:

Serial Number	Test Items	Detected Results	Remark
1	Positive external control	+	
2	Positive internal control	+	
3	Candida albicans	+	
4	Candida tropicalis	-	
5	Candida parapsilosis	-	
6	Candida krusei	-	
7	Candida glabrata	+	
8	Candida auris	-	
9	Aspergillus fumigatus	-	
10	Aspergillus flavus	-	
11	Aspergillus niger	-	
12	Aspergillus terreus	-	
13	Aspergillus nidulans	-	
14	Aspergillus calidoustu	-	
15	Cryptococcus neoformans	-	
16	Cryptococcus gattii	-	
17	Histoplasma capsulatum	-	
18	Lichtheimia corymbifera	-	
19	Mucor pusillus	-	
20	Trichosporon asahii	-	
21	Pneumocystis jirovecii	-	
22	Penicillium marneffei	-	
23	Negative control	-	
24	Blank	-	

Results Suggest:

Candida albicans and Candida glabrata indexes were positive.

Detector__________ Auditor__________ Detection date 2021-01-30 20:22:52

Respiratory fungal nucleic acid test results

Affiliated Hospital Of Guangdong medical university

Basic sample information

Name		Sexuality	Female	Age	79 years old
Section	ICU	Ward		Bed Number	3203
ID		Clinical Diagnosis		Sample Types	sputum
Test Items		Chip Number	c010420166	Sample Number	2021012104

Detailed Results:

Serial Number	Test Items	Detected Results	Remark
1	Positive external control	+	
2	Positive internal control	+	
3	Candida albicans	+	
4	Candida tropicalis	–	
5	Candida parapsilosis	–	
6	Candida krusei	–	
7	Candida glabrata	–	
8	Candida auris	–	
9	Aspergillus fumigatus	–	
10	Aspergillus flavus	–	
11	Aspergillus niger	–	
12	Aspergillus terreus	–	
13	Aspergillus nidulans	–	
14	Aspergillus calidoustu	–	
15	Cryptococcus neoformans	–	
16	Cryptococcus gattii	–	
17	Histoplasma capsulatum	–	
18	Lichtheimia corymbifera	–	
19	Mucor pusillus	–	
20	Trichosporon asahii	–	
21	Pneumocystis jirovecii	–	
22	Penicillium marneffei	–	
23	Negative control	–	
24	Blank	–	

Results Suggest:

Candida albicans index was positive.

Detector ____________ Auditor ____________ Detection date 2021-01-30 23:46:16

Respiratory fungal nucleic acid test results

Affiliated Hospital Of Guangdong medical university

Basic sample information

Name		Sexuality	Male	Age	56 years old
Section	ICU	Ward		Bed Number	206
ID		Clinical Diagnosis		Sample Types	sputum
Test Items		Chip Number	c020420001	Sample Number	2021012105

Detailed Results:

Serial Number	Test Items	Detected Results	Remark
1	Positive external control	+	
2	Positive internal control	+	
3	Candida albicans	–	
4	Candida tropicalis	–	
5	Candida parapsilosis	–	
6	Candida krusei	–	
7	Candida glabrata	–	
8	Candida auris	–	
9	Aspergillus fumigatus	–	
10	Aspergillus flavus	–	
11	Aspergillus niger	–	
12	Aspergillus terreus	–	
13	Aspergillus nidulans	–	
14	Aspergillus calidoustu	–	
15	Cryptococcus neoformans	–	
16	Cryptococcus gattii	–	
17	Histoplasma capsulatum	–	
18	Lichtheimia corymbifera	–	
19	Mucor pusillus	–	
20	Trichosporon asahii	–	
21	Pneumocystis jirovecii	–	
22	Penicillium marneffei	–	
23	Negative control	–	
24	Blank	–	

Results Suggest:

No abnormality was found after detection.

Detector ____________ Auditor ____________ Detection date 2021-01-30 21:34:37

Respiratory fungal nucleic acid test results

Affiliated Hospital Of Guangdong medical university

Basic sample information

Name		Sexuality	Male	Age	36 years old
Section	EICU	Ward		Bed Number	1
ID		Clinical Diagnosis		Sample Types	sputum
Test Items		Chip Number	C020420002	Sample Number	20210201001

Detailed Results:

Serial Number	Test Items	Detected Results	Remark
1	Positive external control	+	
2	Positive internal control	+	
3	Candida albicans	+	
4	Candida tropicalis	+	
5	Candida parapsilosis	−	
6	Candida krusei	−	
7	Candida glabrata	−	
8	Candida auris	−	
9	Aspergillus fumigatus	−	
10	Aspergillus flavus	−	
11	Aspergillus niger	−	
12	Aspergillus terreus	+	
13	Aspergillus nidulans	−	
14	Aspergillus calidoustu	−	
15	Cryptococcus neoformans	−	
16	Cryptococcus gattii	−	
17	Histoplasma capsulatum	−	
18	Lichtheimia corymbifera	−	
19	Mucor pusillus	−	
20	Trichosporon asahii	−	
21	Pneumocystis jirovecii	−	
22	Penicillium marneffei	−	
23	Negative control	−	
24	Blank	−	

Results Suggest:

Candida albicans, Candida tropicalis and Aspergillus terreus indexes were positive.

Detector ________ Auditor ________ Detection date 2021-02-01 16:19:43

Respiratory fungal nucleic acid test results

Affiliated Hospital Of Guangdong medical university

Basic sample information

Name		Sexuality	Female	Age	14 years old
Section	EICU	Ward		Bed Number	2
ID		Clinical Diagnosis		Sample Types	sputum
Test Items		Chip Number	C020420003	Sample Number	20210201002

Detailed Results:

Serial Number	Test Items	Detected Results	Remark
1	Positive external control	+	
2	Positive internal control	+	
3	Candida albicans	−	
4	Candida tropicalis	+	
5	Candida parapsilosis	−	
6	Candida krusei	−	
7	Candida glabrata	−	
8	Candida auris	−	
9	Aspergillus fumigatus	−	
10	Aspergillus flavus	−	
11	Aspergillus niger	−	
12	Aspergillus terreus	−	
13	Aspergillus nidulans	−	
14	Aspergillus calidoustu	−	
15	Cryptococcus neoformans	−	
16	Cryptococcus gattii	−	
17	Histoplasma capsulatum	−	
18	Lichtheimia corymbifera	−	
19	Mucor pusillus	−	
20	Trichosporon asahii	−	
21	Pneumocystis jirovecii	−	
22	Penicillium marneffei	−	
23	Negative control	−	
24	Blank	−	

Results Suggest:

Candida tropicalis index was positive.

Detector ____________ Auditor ____________ Detection date 2021-02-01 17:17:13

Respiratory fungal nucleic acid test results

Affiliated Hospital Of Guangdong medical university

Basic sample information

Name		Sexuality	Male	Age	63 years old
Section	EICU	Ward		Bed Number	3
ID		Clinical Diagnosis		Sample Types	sputum
Test Items		Chip Number	C020420004	Sample Number	20210201003

Detailed Results:

Serial Number	Test Items	Detected Results	Remark
1	Positive external control	+	
2	Positive internal control	+	
3	Candida albicans	+	
4	Candida tropicalis	+	
5	Candida parapsilosis	-	
6	Candida krusei	-	
7	Candida glabrata	-	
8	Candida auris	-	
9	Aspergillus fumigatus	-	
10	Aspergillus flavus	-	
11	Aspergillus niger	-	
12	Aspergillus terreus	-	
13	Aspergillus nidulans	-	
14	Aspergillus calidoustu	-	
15	Cryptococcus neoformans	-	
16	Cryptococcus gattii	-	
17	Histoplasma capsulatum	-	
18	Lichtheimia corymbifera	-	
19	Mucor pusillus	-	
20	Trichosporon asahii	-	
21	Pneumocystis jirovecii	-	
22	Penicillium marneffei	-	
23	Negative control	-	
24	Blank	-	

Results Suggest:

Candida albicans and Candida tropicalis indexes were positive.

Detector ____________ Auditor ____________ Detection date 2021-02-01 18:18:09

Respiratory fungal nucleic acid test results

Affiliated Hospital Of Guangdong medical university

Basic sample information

Name		Sexuality	Male	Age	39 years old
Section	EICU	Ward		Bed Number	4
ID		Clinical Diagnosis		Sample Types	sputum
Test Items		Chip Number	ZJ-202011010239	Sample Number	2021020104

Detailed Results:

Serial Number	Test Items	Detected Results	Remark
1	Positive external control	+	
2	Positive internal control	+	
3	Candida albicans	-	
4	Candida tropicalis	-	
5	Candida parapsilosis	-	
6	Candida krusei	-	
7	Candida glabrata	+	
8	Candida auris	-	
9	Aspergillus fumigatus	-	
10	Aspergillus flavus	-	
11	Aspergillus niger	-	
12	Aspergillus terreus	-	
13	Aspergillus nidulans	-	
14	Aspergillus calidoustu	-	
15	Cryptococcus neoformans	-	
16	Cryptococcus gattii	-	
17	Histoplasma capsulatum	-	
18	Lichtheimia corymbifera	-	
19	Mucor pusillus	-	
20	Trichosporon asahii	-	
21	Pneumocystis jirovecii	-	
22	Penicillium marneffei	-	
23	Negative control	-	
24	Blank	-	

Results Suggest:

Candida glabrata index was positive.

Detector ____________ Auditor ____________ Detection date 2021-02-02 09:22:35

Respiratory fungal nucleic acid test results

Affiliated Hospital Of Guangdong medical university

Basic sample information

Name		Sexuality	Male	Age	53 years old
Section	ICU	Ward		Bed Number	6
ID		Clinical Diagnosis		Sample Types	sputum
Test Items		Chip Number	ZJ-202011010227	Sample Number	2021020108

Detailed Results:

Serial Number	Test Items	Detected Results	Remark
1	Positive external control	+	
2	Positive internal control	+	
3	Candida albicans	+	
4	Candida tropicalis	-	
5	Candida parapsilosis	-	
6	Candida krusei	-	
7	Candida glabrata	-	
8	Candida auris	-	
9	Aspergillus fumigatus	-	
10	Aspergillus flavus	-	
11	Aspergillus niger	-	
12	Aspergillus terreus	-	
13	Aspergillus nidulans	-	
14	Aspergillus calidoustu	-	
15	Cryptococcus neoformans	-	
16	Cryptococcus gattii	-	
17	Histoplasma capsulatum	-	
18	Lichtheimia corymbifera	-	
19	Mucor pusillus	-	
20	Trichosporon asahii	-	
21	Pneumocystis jirovecii	-	
22	Penicillium marneffei	-	
23	Negative control	-	
24	Blank	-	

Results Suggest:

Candida albicans index was positive.

Detector ____________ Auditor ____________ Detection date 2021-02-02 10:17:23

Respiratory fungal nucleic acid test results

Affiliated Hospital Of Guangdong medical university

Basic sample information

Name		Sexuality	Male	Age	64 years old
Section	ICU	Ward		Bed Number	12
ID		Clinical Diagnosis		Sample Types	sputum
Test Items		Chip Number	ZJ-202011010232	Sample Number	2021020107

Detailed Results:

Serial Number	Test Items	Detected Results	Remark
1	Positive external control	+	
2	Positive internal control	+	
3	Candida albicans	-	
4	Candida tropicalis	-	
5	Candida parapsilosis	-	
6	Candida krusei	-	
7	Candida glabrata	-	
8	Candida auris	-	
9	Aspergillus fumigatus	-	
10	Aspergillus flavus	-	
11	Aspergillus niger	-	
12	Aspergillus terreus	-	
13	Aspergillus nidulans	-	
14	Aspergillus calidoustu	-	
15	Cryptococcus neoformans	-	
16	Cryptococcus gattii	-	
17	Histoplasma capsulatum	-	
18	Lichtheimia corymbifera	-	
19	Mucor pusillus	-	
20	Trichosporon asahii	-	
21	Pneumocystis jirovecii	-	
22	Penicillium marneffei	-	
23	Negative control	-	
24	Blank	-	

Results Suggest:

No abnormality was found after detection.

Detector ____________ Auditor ____________ Detection date 2021-02-02 16:08:55

Respiratory fungal nucleic acid test results

Affiliated Hospital Of Guangdong medical university

Basic sample information

Name		Sexuality	Female	Age	55 years old
Section	ICU	Ward		Bed Number	208
ID		Clinical Diagnosis		Sample Types	sputum
Test Items		Chip Number	ZJ-202011010229	Sample Number	20210202004

Detailed Results:

Serial Number	Test Items	Detected Results	Remark
1	Positive external control	+	
2	Positive internal control	+	
3	Candida albicans	+	
4	Candida tropicalis	-	
5	Candida parapsilosis	-	
6	Candida krusei	-	
7	Candida glabrata	-	
8	Candida auris	-	
9	Aspergillus fumigatus	-	
10	Aspergillus flavus	-	
11	Aspergillus niger	-	
12	Aspergillus terreus	-	
13	Aspergillus nidulans	-	
14	Aspergillus calidoustu	-	
15	Cryptococcus neoformans	-	
16	Cryptococcus gattii	-	
17	Histoplasma capsulatum	-	
18	Lichtheimia corymbifera	-	
19	Mucor pusillus	-	
20	Trichosporon asahii	-	
21	Pneumocystis jirovecii	-	
22	Penicillium marneffei	-	
23	Negative control	-	
24	Blank	-	

Results Suggest:

Candida albicans index was positive.

Detector ______ Auditor ______ Detection date 2021-02-02 12:57:22

Respiratory fungal nucleic acid test results

Affiliated Hospital Of Guangdong medical university

Basic sample information

Name		Sexuality	Male	Age	47 years old
Section	ICU	Ward		Bed Number	302
ID		Clinical Diagnosis		Sample Types	sputum
Test Items		Chip Number	ZJ-202011010230	Sample Number	20210202005

Detailed Results:

Serial Number	Test Items	Detected Results	Remark
1	Positive external control	+	
2	Positive internal control	+	
3	Candida albicans	-	
4	Candida tropicalis	-	
5	Candida parapsilosis	-	
6	Candida krusei	-	
7	Candida glabrata	-	
8	Candida auris	-	
9	Aspergillus fumigatus	-	
10	Aspergillus flavus	-	
11	Aspergillus niger	-	
12	Aspergillus terreus	-	
13	Aspergillus nidulans	-	
14	Aspergillus calidoustu	-	
15	Cryptococcus neoformans	-	
16	Cryptococcus gattii	-	
17	Histoplasma capsulatum	-	
18	Lichtheimia corymbifera	-	
19	Mucor pusillus	-	
20	Trichosporon asahii	-	
21	Pneumocystis jirovecii	-	
22	Penicillium marneffei	-	
23	Negative control	-	
24	Blank	-	

Results Suggest:

No abnormality was found after detection.

Detector ____________ Auditor ____________ Detection date 2021-02-02 14:15:20

Respiratory fungal nucleic acid test results

Affiliated Hospital Of Guangdong medical university

Basic sample information

Name		Sexuality	Female	Age	69 years old
Section	ICU	Ward		Bed Number	308
ID		Clinical Diagnosis		Sample Types	sputum
Test Items		Chip Number	ZJ-202011010231	Sample Number	2021020105

Detailed Results:

Serial Number	Test Items	Detected Results	Remark
1	Positive external control	+	
2	Positive internal control	+	
3	Candida albicans	+	
4	Candida tropicalis	-	
5	Candida parapsilosis	-	
6	Candida krusei	-	
7	Candida glabrata	-	
8	Candida auris	-	
9	Aspergillus fumigatus	-	
10	Aspergillus flavus	-	
11	Aspergillus niger	-	
12	Aspergillus terreus	-	
13	Aspergillus nidulans	-	
14	Aspergillus calidoustu	-	
15	Cryptococcus neoformans	-	
16	Cryptococcus gattii	-	
17	Histoplasma capsulatum	-	
18	Lichtheimia corymbifera	-	
19	Mucor pusillus	-	
20	Trichosporon asahii	-	
21	Pneumocystis jirovecii	-	
22	Penicillium marneffei	-	
23	Negative control	-	
24	Blank	-	

Results Suggest:

Candida albicans index was positive.

Detector ____________ Auditor ____________ Detection date 2021-02-02 15:13:00

Respiratory fungal nucleic acid test results

Affiliated Hospital Of Guangdong medical university

Basic sample information

Name		Sexuality	Male	Age	69 years old
Section	department of respiratory medicine	Ward		Bed Number	3001
ID		Clinical Diagnosis		Sample Types	sputum
Test Items		Chip Number	ZJ-202011010234	Sample Number	2021020301

Detailed Results:

Serial Number	Test Items	Detected Results	Remark
1	Positive external control	+	
2	Positive internal control	+	
3	Candida albicans	+	
4	Candida tropicalis	–	
5	Candida parapsilosis	–	
6	Candida krusei	–	
7	Candida glabrata	–	
8	Candida auris	–	
9	Aspergillus fumigatus	–	
10	Aspergillus flavus	–	
11	Aspergillus niger	–	
12	Aspergillus terreus	–	
13	Aspergillus nidulans	–	
14	Aspergillus calidoustu	–	
15	Cryptococcus neoformans	–	
16	Cryptococcus gattii	–	
17	Histoplasma capsulatum	–	
18	Lichtheimia corymbifera	–	
19	Mucor pusillus	–	
20	Trichosporon asahii	–	
21	Pneumocystis jirovecii	–	
22	Penicillium marneffei	–	
23	Negative control	–	
24	Blank	–	

Results Suggest:

Candida albicans index was positive.

Detector ____________ Auditor ____________ Detection date 2021-02-03 17:35:56

Respiratory fungal nucleic acid test results

Affiliated Hospital Of Guangdong medical university

Basic sample information

Name		Sexuality	Male	Age	43 years old
Section	department of respiratory medicine	Ward		Bed Number	3009
ID		Clinical Diagnosis		Sample Types	sputum
Test Items		Chip Number	ZJ-202011010235	Sample Number	2021020302

Detailed Results:

Serial Number	Test Items	Detected Results	Remark
1	Positive external control	+	
2	Positive internal control	+	
3	Candida albicans	-	
4	Candida tropicalis	-	
5	Candida parapsilosis	-	
6	Candida krusei	-	
7	Candida glabrata	+	
8	Candida auris	-	
9	Aspergillus fumigatus	-	
10	Aspergillus flavus	-	
11	Aspergillus niger	-	
12	Aspergillus terreus	-	
13	Aspergillus nidulans	-	
14	Aspergillus calidoustu	-	
15	Cryptococcus neoformans	-	
16	Cryptococcus gattii	-	
17	Histoplasma capsulatum	-	
18	Lichtheimia corymbifera	-	
19	Mucor pusillus	-	
20	Trichosporon asahii	-	
21	Pneumocystis jirovecii	-	
22	Penicillium marneffei	-	
23	Negative control	-	
24	Blank	-	

Results Suggest:

Candida glabrata index was positive.

Detector ____________ Auditor ____________ Detection date 2021-02-03 17:43:43

Respiratory fungal nucleic acid test results

Affiliated Hospital Of Guangdong medical university

Basic sample information

Name		Sexuality	Female	Age	54 years old
Section	ICU	Ward		Bed Number	4
ID		Clinical Diagnosis		Sample Types	sputum
Test Items		Chip Number	ZJ-202011010238	Sample Number	2021020501

Detailed Results:

Serial Number	Test Items	Detected Results	Remark
1	Positive external control	+	
2	Positive internal control	+	
3	Candida albicans	-	
4	Candida tropicalis	-	
5	Candida parapsilosis	-	
6	Candida krusei	-	
7	Candida glabrata	-	
8	Candida auris	-	
9	Aspergillus fumigatus	-	
10	Aspergillus flavus	-	
11	Aspergillus niger	-	
12	Aspergillus terreus	-	
13	Aspergillus nidulans	-	
14	Aspergillus calidoustu	-	
15	Cryptococcus neoformans	-	
16	Cryptococcus gattii	-	
17	Histoplasma capsulatum	-	
18	Lichtheimia corymbifera	-	
19	Mucor pusillus	-	
20	Trichosporon asahii	-	
21	Pneumocystis jirovecii	-	
22	Penicillium marneffei	-	
23	Negative control	-	
24	Blank	-	

Results Suggest:

No abnormality was found after detection.

Detector ____________ Auditor ____________ Detection date 2021-02-07 09:05:38

Respiratory fungal nucleic acid test results

Affiliated Hospital Of Guangdong medical university

Basic sample information

Name		Sexuality	Male	Age	71 years old
Section	ICU			Bed Number	9
ID		Clinical Diagnosis		Sample Types	sputum
Test Items		Chip Number	ZJ-202011010237	Sample Number	2021020502

Detailed Results:

Serial Number	Test Items	Detected Results	Remark
1	Positive external control	+	
2	Positive internal control	+	
3	Candida albicans	+	
4	Candida tropicalis	-	
5	Candida parapsilosis	-	
6	Candida krusei	-	
7	Candida glabrata	+	
8	Candida auris	-	
9	Aspergillus fumigatus	-	
10	Aspergillus flavus	-	
11	Aspergillus niger	-	
12	Aspergillus terreus	-	
13	Aspergillus nidulans	-	
14	Aspergillus calidoustu	-	
15	Cryptococcus neoformans	-	
16	Cryptococcus gattii	-	
17	Histoplasma capsulatum	-	
18	Lichtheimia corymbifera	-	
19	Mucor pusillus	-	
20	Trichosporon asahii	-	
21	Pneumocystis jirovecii	-	
22	Penicillium marneffei	-	
23	Negative control	-	
24	Blank	-	

Results Suggest:

Candida albicans and Candida glabrata indexes were positive.

Detector ____________ Auditor ____________ Detection date 2021-02-05 16:24:45

Respiratory fungal nucleic acid test results

Affiliated Hospital Of Guangdong Medical University

Basic sample information

Name		Sexuality	Male	Age	79 years old
Section	ICU	Ward		Bed Number	10
ID		Clinical Diagnosis		Sample Types	sputum
Test Items		Chip Number	ZJ-202011010236	Sample Number	2021020503

Detailed Results:

Serial Number	Test Items	Detected Results	Remark
1	Positive external control	+	
2	Positive internal control	+	
3	Candida albicans	+	
4	Candida tropicalis	-	
5	Candida parapsilosis	-	
6	Candida krusei	-	
7	Candida glabrata	+	
8	Candida auris	-	
9	Aspergillus fumigatus	-	
10	Aspergillus flavus	-	
11	Aspergillus niger	-	
12	Aspergillus terreus	-	
13	Aspergillus nidulans	-	
14	Aspergillus calidoustu	-	
15	Cryptococcus neoformans	-	
16	Cryptococcus gattii	-	
17	Histoplasma capsulatum	-	
18	Lichtheimia corymbifera	-	
19	Mucor pusillus	-	
20	Trichosporon asahii	-	
21	Pneumocystis jirovecii	-	
22	Penicillium marneffei	-	
23	Negative control	-	
24	Blank	-	

Results Suggest:

Candida albicans and Candida glabrata indexes were positive.

Detector ____________ Auditor ____________ Detection date 2021-02-05 17:30:38

Respiratory fungal nucleic acid test results

Affiliated Hospital Of Guangdong Medical University

Basic sample information

Name		Sexuality	Female	Age	57 years old
Section	ICU	Ward		Bed Number	302
ID		Clinical Diagnosis		Sample Types	sputum
Test Items		Chip Number	ZJ-202011010449	Sample Number	2021020504

Detailed Results:

Serial Number	Test Items	Detected Results	Remark
1	Positive external control	+	
2	Positive internal control	+	
3	Candida albicans	-	
4	Candida tropicalis	-	
5	Candida parapsilosis	-	
6	Candida krusei	-	
7	Candida glabrata	-	
8	Candida auris	-	
9	Aspergillus fumigatus	-	
10	Aspergillus flavus	-	
11	Aspergillus niger	-	
12	Aspergillus terreus	-	
13	Aspergillus nidulans	-	
14	Aspergillus calidoustu	-	
15	Cryptococcus neoformans	-	
16	Cryptococcus gattii	-	
17	Histoplasma capsulatum	-	
18	Lichtheimia corymbifera	-	
19	Mucor pusillus	-	
20	Trichosporon asahii	-	
21	Pneumocystis jirovecii	-	
22	Penicillium marneffei	-	
23	Negative control	-	
24	Blank	-	

Results Suggest:

No abnormality was found after detection.

Detector ________ Auditor ________ Detection date 2021-02-05 18:28:02

Respiratory fungal nucleic acid test results

Affiliated Hospital Of Guangdong Medical University

Basic sample information

Name		Sexuality	Male	Age	59 years old
Section	ICU	Ward		Bed Number	307
ID		Clinical Diagnosis		Sample Types	sputum
Test Items		Chip Number	ZJ-202011010450	Sample Number	2021020505

Detailed Results:

Serial Number	Test Items	Detected Results	Remark
1	Positive external control	+	
2	Positive internal control	+	
3	Candida albicans	-	
4	Candida tropicalis	-	
5	Candida parapsilosis	-	
6	Candida krusei	-	
7	Candida glabrata	-	
8	Candida auris	-	
9	Aspergillus fumigatus	-	
10	Aspergillus flavus	-	
11	Aspergillus niger	-	
12	Aspergillus terreus	-	
13	Aspergillus nidulans	-	
14	Aspergillus calidoustu	-	
15	Cryptococcus neoformans	-	
16	Cryptococcus gattii	-	
17	Histoplasma capsulatum	-	
18	Lichtheimia corymbifera	-	
19	Mucor pusillus	-	
20	Trichosporon asahii	-	
21	Pneumocystis jirovecii	-	
22	Penicillium marneffei	-	
23	Negative control	-	
24	Blank	-	

Results Suggest:

No abnormality was found after detection.

Detector ____________ Auditor ____________ Detection date 2021-02-05 19:28:02

Respiratory fungal nucleic acid test results

Affiliated Hospital Of Guangdong Medical University

Basic sample information

Name		Sexuality	Male	Age	66 years old
Section	ICU	Ward		Bed Number	1
ID		Clinical Diagnosis		Sample Types	sputum
Test Items		Chip Number	ZJ-202011010451	Sample Number	2021020801

Detailed Results:

Serial Number	Test Items	Detected Results	Remark
1	Positive external control	+	
2	Positive internal control	+	
3	Candida albicans	+	
4	Candida tropicalis	–	
5	Candida parapsilosis	–	
6	Candida krusei	–	
7	Candida glabrata	+	
8	Candida auris	–	
9	Aspergillus fumigatus	–	
10	Aspergillus flavus	–	
11	Aspergillus niger	–	
12	Aspergillus terreus	–	
13	Aspergillus nidulans	–	
14	Aspergillus calidoustu	–	
15	Cryptococcus neoformans	–	
16	Cryptococcus gattii	–	
17	Histoplasma capsulatum	–	
18	Lichtheimia corymbifera	–	
19	Mucor pusillus	–	
20	Trichosporon asahii	–	
21	Pneumocystis jirovecii	–	
22	Penicillium marneffei	–	
23	Negative control	–	
24	Blank	–	

Results Suggest:

Candida albicans and Candida glabrata indexes were positive.

Detector ____________ Auditor ____________ Detection date 2021-02-09 19:58:14

Respiratory fungal nucleic acid test results

Affiliated Hospital Of Guangdong Medical University

Basic sample information

Name		Sexuality	Male	Age	52 years old
Section	ICU	Ward		Bed Number	2
ID		Clinical Diagnosis		Sample Types	sputum
Test Items		Chip Number	ZJ-202011010452	Sample Number	2021020802

Detailed Results:

Serial Number	Test Items	Detected Results	Remark
1	Positive external control	+	
2	Positive internal control	+	
3	Candida albicans	-	
4	Candida tropicalis	-	
5	Candida parapsilosis	-	
6	Candida krusei	-	
7	Candida glabrata	+	
8	Candida auris	-	
9	Aspergillus fumigatus	-	
10	Aspergillus flavus	-	
11	Aspergillus niger	-	
12	Aspergillus terreus	-	
13	Aspergillus nidulans	-	
14	Aspergillus calidoustu	-	
15	Cryptococcus neoformans	-	
16	Cryptococcus gattii	-	
17	Histoplasma capsulatum	-	
18	Lichtheimia corymbifera	-	
19	Mucor pusillus	-	
20	Trichosporon asahii	-	
21	Pneumocystis jirovecii	-	
22	Penicillium marneffei	-	
23	Negative control	-	
24	Blank	-	

Results Suggest:

Candida glabrata index was positive.

Detector ____________ Auditor ____________ Detection date 2021-02-09 20:02:10

Respiratory fungal nucleic acid test results

Affiliated Hospital Of Guangdong Medical University

Basic sample information

Name		Sexuality	Female	Age	54 years old
Section	ICU	Ward		Bed Number	6
ID		Clinical Diagnosis		Sample Types	sputum
Test Items		Chip Number	ZJ-202011010453	Sample Number	2021020803

Detailed Results:

Serial Number	Test Items	Detected Results	Remark
1	Positive external control	+	
2	Positive internal control	+	
3	Candida albicans	-	
4	Candida tropicalis	-	
5	Candida parapsilosis	-	
6	Candida krusei	-	
7	Candida glabrata	-	
8	Candida auris	-	
9	Aspergillus fumigatus	-	
10	Aspergillus flavus	-	
11	Aspergillus niger	-	
12	Aspergillus terreus	-	
13	Aspergillus nidulans	-	
14	Aspergillus calidoustu	-	
15	Cryptococcus neoformans	-	
16	Cryptococcus gattii	-	
17	Histoplasma capsulatum	-	
18	Lichtheimia corymbifera	-	
19	Mucor pusillus	-	
20	Trichosporon asahii	-	
21	Pneumocystis jirovecii	-	
22	Penicillium marneffei	-	
23	Negative control	-	
24	Blank	-	

Results Suggest:

No abnormality was found after detection.

Detector ________ Auditor ________ Detection date 2021-02-09 17:41:19

Respiratory fungal nucleic acid test results

Affiliated Hospital Of Guangdong Medical University

Basic sample information

Name		Sexuality	Female	Age	67 years old
Section	ICU	Ward		Bed Number	7
ID		Clinical Diagnosis		Sample Types	sputum
Test Items		Chip Number	ZJ-202011010454	Sample Number	2021020804

Detailed Results:

Serial Number	Test Items	Detected Results	Remark
1	Positive external control	+	
2	Positive internal control	+	
3	Candida albicans	-	
4	Candida tropicalis	-	
5	Candida parapsilosis	-	
6	Candida krusei	-	
7	Candida glabrata	-	
8	Candida auris	-	
9	Aspergillus fumigatus	+	
10	Aspergillus flavus	-	
11	Aspergillus niger	-	
12	Aspergillus terreus	-	
13	Aspergillus nidulans	-	
14	Aspergillus calidoustu	-	
15	Cryptococcus neoformans	-	
16	Cryptococcus gattii	-	
17	Histoplasma capsulatum	-	
18	Lichtheimia corymbifera	-	
19	Mucor pusillus	-	
20	Trichosporon asahii	-	
21	Pneumocystis jirovecii	-	
22	Penicillium marneffei	-	
23	Negative control	-	
24	Blank	-	

Results Suggest:

Aspergillus fumigatus index was positive.

Detector ____________ Auditor ____________ Detection date 2021-02-09 19:23:13

Respiratory fungal nucleic acid test results

Affiliated Hospital Of Guangdong Medical University

Basic sample information

Name		Sexuality	Male	Age	62 years old
Section	ICU	Ward		Bed Number	3
ID		Clinical Diagnosis		Sample Types	bronchoalveolar lavage fluid
Test Items		Chip Number	ZJ-202011010455	Sample Number	2021022601

Detailed Results:

Serial Number	Test Items	Detected Results	Remark
1	Positive external control	+	
2	Positive internal control	+	
3	Candida albicans	-	
4	Candida tropicalis	-	
5	Candida parapsilosis	-	
6	Candida krusei	-	
7	Candida glabrata	-	
8	Candida auris	-	
9	Aspergillus fumigatus	-	
10	Aspergillus flavus	-	
11	Aspergillus niger	-	
12	Aspergillus terreus	-	
13	Aspergillus nidulans	-	
14	Aspergillus calidoustu	-	
15	Cryptococcus neoformans	-	
16	Cryptococcus gattii	-	
17	Histoplasma capsulatum	-	
18	Lichtheimia corymbifera	-	
19	Mucor pusillus	-	
20	Trichosporon asahii	-	
21	Pneumocystis jirovecii	-	
22	Penicillium marneffei	-	
23	Negative control	-	
24	Blank	-	

Results Suggest:

No abnormality was found after detection.

Detector ________ Auditor ________ Detection date 2021-02-27 15:58:44

Respiratory fungal nucleic acid test results

Affiliated Hospital Of Guangdong Medical University

Basic sample information

Name		Sexuality	Male	Age	53 years old
Section	ICU	Ward		Bed Number	10
ID		Clinical Diagnosis		Sample Types	sputum
Test Items		Chip Number	ZJ-202011010456	Sample Number	2021022602

Detailed Results:

Serial Number	Test Items	Detected Results	Remark
1	Positive external control	+	
2	Positive internal control	+	
3	Candida albicans	+	
4	Candida tropicalis	-	
5	Candida parapsilosis	-	
6	Candida krusei	-	
7	Candida glabrata	-	
8	Candida auris	-	
9	Aspergillus fumigatus	-	
10	Aspergillus flavus	-	
11	Aspergillus niger	-	
12	Aspergillus terreus	-	
13	Aspergillus nidulans	-	
14	Aspergillus calidoustu	-	
15	Cryptococcus neoformans	-	
16	Cryptococcus gattii	-	
17	Histoplasma capsulatum	-	
18	Lichtheimia corymbifera	-	
19	Mucor pusillus	-	
20	Trichosporon asahii	-	
21	Pneumocystis jirovecii	-	
22	Penicillium marneffei	-	
23	Negative control	-	
24	Blank	-	

Results Suggest:

Candida albicans index was positive.

Detector ____________ Auditor ____________ Detection date 2021-02-27 17:04:48

Respiratory fungal nucleic acid test results

Affiliated Hospital Of Guangdong Medical University

Basic sample information

Name		Sexuality	Male	Age	26 years old
Section	ICU	Ward		Bed Number	203
ID		Clinical Diagnosis		Sample Types	sputum
Test Items		Chip Number	ZJ-202011010457	Sample Number	2021022603

Detailed Results:

Serial Number	Test Items	Detected Results	Remark
1	Positive external control	+	
2	Positive internal control	+	
3	Candida albicans	+	
4	Candida tropicalis	+	
5	Candida parapsilosis	-	
6	Candida krusei	-	
7	Candida glabrata	-	
8	Candida auris	-	
9	Aspergillus fumigatus	-	
10	Aspergillus flavus	-	
11	Aspergillus niger	-	
12	Aspergillus terreus	-	
13	Aspergillus nidulans	-	
14	Aspergillus calidoustu	-	
15	Cryptococcus neoformans	-	
16	Cryptococcus gattii	-	
17	Histoplasma capsulatum	-	
18	Lichtheimia corymbifera	-	
19	Mucor pusillus	-	
20	Trichosporon asahii	-	
21	Pneumocystis jirovecii	-	
22	Penicillium marneffei	-	
23	Negative control	-	
24	Blank	-	

Results Suggest:

Candida albicans and Candida tropicalis indexes were positive.

Detector ________ Auditor ________ Detection date 2021-02-27 18:11:08

Respiratory fungal nucleic acid test results

Affiliated Hospital Of Guangdong Medical University

Basic sample information

Name		Sexuality	Female	Age	53 years old
Section	ICU	Ward		Bed Number	205
ID		Clinical Diagnosis		Sample Types	sputum
Test Items		Chip Number	ZJ-202011010458	Sample Number	2021022604

Detailed Results:

Serial Number	Test Items	Detected Results	Remark
1	Positive external control	+	
2	Positive internal control	+	
3	Candida albicans	-	
4	Candida tropicalis	-	
5	Candida parapsilosis	-	
6	Candida krusei	-	
7	Candida glabrata	-	
8	Candida auris	-	
9	Aspergillus fumigatus	-	
10	Aspergillus flavus	-	
11	Aspergillus niger	-	
12	Aspergillus terreus	-	
13	Aspergillus nidulans	-	
14	Aspergillus calidoustu	-	
15	Cryptococcus neoformans	-	
16	Cryptococcus gattii	-	
17	Histoplasma capsulatum	-	
18	Lichtheimia corymbifera	-	
19	Mucor pusillus	-	
20	Trichosporon asahii	-	
21	Pneumocystis jirovecii	-	
22	Penicillium marneffei	-	
23	Negative control	-	
24	Blank	-	

Results Suggest:

No abnormality was found after detection.

Detector ______ Auditor ______ Detection date 2021-02-27 21:02:22

Respiratory fungal nucleic acid test results

Affiliated Hospital Of Guangdong Medical University

Basic sample information

Name		Sexuality	Male	Age	87 years old
Section	ICU	Ward		Bed Number	301
ID		Clinical Diagnosis		Sample Types	sputum
Test Items		Chip Number	ZJ-202011010459	Sample Number	2021022605

Detailed Results:

Serial Number	Test Items	Detected Results	Remark
1	Positive external control	+	
2	Positive internal control	+	
3	Candida albicans	+	
4	Candida tropicalis	-	
5	Candida parapsilosis	-	
6	Candida krusei	-	
7	Candida glabrata	-	
8	Candida auris	-	
9	Aspergillus fumigatus	+	
10	Aspergillus flavus	-	
11	Aspergillus niger	-	
12	Aspergillus terreus	-	
13	Aspergillus nidulans	-	
14	Aspergillus calidoustu	-	
15	Cryptococcus neoformans	-	
16	Cryptococcus gattii	-	
17	Histoplasma capsulatum	-	
18	Lichtheimia corymbifera	-	
19	Mucor pusillus	-	
20	Trichosporon asahii	-	
21	Pneumocystis jirovecii	-	
22	Penicillium marneffei	-	
23	Negative control	-	
24	Blank	-	

Results Suggest:

Candida albicans and Aspergillus fumigatus indexes were positive.

Detector ________ Auditor ________ Detection date 2021-02-27 20:02:08

Respiratory fungal nucleic acid test results

Affiliated Hospital Of Guangdong Medical University

Basic sample information

Name		Sexuality	Male	Age	72 years old
Section	ICU	Ward		Bed Number	312
ID		Clinical Diagnosis		Sample Types	sputum
Test Items		Chip Number	ZJ-202011010460	Sample Number	2021022606

Detailed Results:

Serial Number	Test Items	Detected Results	Remark
1	Positive external control	+	
2	Positive internal control	+	
3	Candida albicans	–	
4	Candida tropicalis	–	
5	Candida parapsilosis	–	
6	Candida krusei	–	
7	Candida glabrata	–	
8	Candida auris	–	
9	Aspergillus fumigatus	–	
10	Aspergillus flavus	–	
11	Aspergillus niger	–	
12	Aspergillus terreus	–	
13	Aspergillus nidulans	–	
14	Aspergillus calidoustu	–	
15	Cryptococcus neoformans	–	
16	Cryptococcus gattii	–	
17	Histoplasma capsulatum	–	
18	Lichtheimia corymbifera	–	
19	Mucor pusillus	–	
20	Trichosporon asahii	–	
21	Pneumocystis jirovecii	–	
22	Penicillium marneffei	–	
23	Negative control	–	
24	Blank	–	

Results Suggest:

No abnormality was found after detection.

Detector ____________ Auditor ____________ Detection date 2021-02-27 20:56:58

Respiratory fungal nucleic acid test results

Affiliated Hospital Of Guangdong Medical University

Basic sample information

Name		Sexuality	Male	Age	78 years old
Section	ICU	Ward		Bed Number	3001
ID		Clinical Diagnosis		Sample Types	sputum
Test Items		Chip Number	ZJ-202011010278	Sample Number	2021030402

Detailed Results:

Serial Number	Test Items	Detected Results	Remark
1	Positive external control	+	
2	Positive internal control	+	
3	Candida albicans	-	
4	Candida tropicalis	-	
5	Candida parapsilosis	-	
6	Candida krusei	-	
7	Candida glabrata	-	
8	Candida auris	-	
9	Aspergillus fumigatus	-	
10	Aspergillus flavus	-	
11	Aspergillus niger	-	
12	Aspergillus terreus	-	
13	Aspergillus nidulans	-	
14	Aspergillus calidoustu	-	
15	Cryptococcus neoformans	-	
16	Cryptococcus gattii	-	
17	Histoplasma capsulatum	-	
18	Lichtheimia corymbifera	-	
19	Mucor pusillus	-	
20	Trichosporon asahii	-	
21	Pneumocystis jirovecii	-	
22	Penicillium marneffei	-	
23	Negative control	-	
24	Blank	-	

Results Suggest:

No abnormality was found after detection.

Detector ____________ Auditor ____________ Detection date 2021-03-05 09:04:23

Respiratory fungal nucleic acid test results

Affiliated Hospital Of Guangdong Medical University

Basic sample information

Name		Sexuality	Male	Age	30 years old
Section	ICU	Ward		Bed Number	204
ID		Clinical Diagnosis		Sample Types	sputum
Test Items		Chip Number	ZJ-202011010279	Sample Number	2021030401

Detailed Results:

Serial Number	Test Items	Detected Results	Remark
1	Positive external control	+	
2	Positive internal control	+	
3	Candida albicans	-	
4	Candida tropicalis	-	
5	Candida parapsilosis	-	
6	Candida krusei	-	
7	Candida glabrata	-	
8	Candida auris	-	
9	Aspergillus fumigatus	-	
10	Aspergillus flavus	-	
11	Aspergillus niger	-	
12	Aspergillus terreus	-	
13	Aspergillus nidulans	-	
14	Aspergillus calidoustu	-	
15	Cryptococcus neoformans	-	
16	Cryptococcus gattii	-	
17	Histoplasma capsulatum	-	
18	Lichtheimia corymbifera	-	
19	Mucor pusillus	-	
20	Trichosporon asahii	-	
21	Pneumocystis jirovecii	-	
22	Penicillium marneffei	-	
23	Negative control	-	
24	Blank	-	

Results Suggest:

No abnormality was found after detection.

Detector ____________ Auditor ____________ Detection date 2021-03-05 09:59:17

Respiratory fungal nucleic acid test results

Affiliated Hospital Of Guangdong Medical University

Basic sample information

Name		Sexuality	Female	Age	39 years old
Section	ICU	Ward		Bed Number	4
ID		Clinical Diagnosis		Sample Types	pleuroperitoneal fluid
Test Items		Chip Number	ZJ-202011010280	Sample Number	2021030404

Detailed Results:

Serial Number	Test Items	Detected Results	Remark
1	Positive external control	+	
2	Positive internal control	+	
3	Candida albicans	-	
4	Candida tropicalis	-	
5	Candida parapsilosis	-	
6	Candida krusei	-	
7	Candida glabrata	-	
8	Candida auris	-	
9	Aspergillus fumigatus	-	
10	Aspergillus flavus	-	
11	Aspergillus niger	-	
12	Aspergillus terreus	-	
13	Aspergillus nidulans	-	
14	Aspergillus calidoustu	-	
15	Cryptococcus neoformans	-	
16	Cryptococcus gattii	-	
17	Histoplasma capsulatum	-	
18	Lichtheimia corymbifera	-	
19	Mucor pusillus	-	
20	Trichosporon asahii	-	
21	Pneumocystis jirovecii	-	
22	Penicillium marneffei	-	
23	Negative control	-	
24	Blank	-	

Results Suggest:

No abnormality was found after detection.

Detector ____________ Auditor ____________ Detection date 2021-03-05 10:55:55

Respiratory fungal nucleic acid test results

Affiliated Hospital Of Guangdong Medical University

Basic sample information

Name		Sexuality	Male	Age	51 years old
Section	EICU	Ward		Bed Number	1
ID		Clinical Diagnosis		Sample Types	sputum
Test Items		Chip Number	ZJ-202011010281	Sample Number	2021030901

Detailed Results:

Serial Number	Test Items	Detected Results	Remark
1	Positive external control	+	
2	Positive internal control	+	
3	Candida albicans	-	
4	Candida tropicalis	-	
5	Candida parapsilosis	-	
6	Candida krusei	-	
7	Candida glabrata	-	
8	Candida auris	-	
9	Aspergillus fumigatus	-	
10	Aspergillus flavus	-	
11	Aspergillus niger	-	
12	Aspergillus terreus	-	
13	Aspergillus nidulans	-	
14	Aspergillus calidoustu	-	
15	Cryptococcus neoformans	-	
16	Cryptococcus gattii	-	
17	Histoplasma capsulatum	-	
18	Lichtheimia corymbifera	-	
19	Mucor pusillus	-	
20	Trichosporon asahii	-	
21	Pneumocystis jirovecii	-	
22	Penicillium marneffei	-	
23	Negative control	-	
24	Blank	-	

Results Suggest:

No abnormality was found after detection.

Detector ____________ Auditor ____________ Detection date 2021-03-10 10:15:32

Respiratory fungal nucleic acid test results

Affiliated Hospital Of Guangdong Medical University

Basic sample information

Name		Sexuality	Female	Age	80 years old
Section	EICU	Ward		Bed Number	3
ID		Clinical Diagnosis		Sample Types	sputum
Test Items		Chip Number	ZJ-202011010282	Sample Number	2020030902

Detailed Results:

Serial Number	Test Items	Detected Results	Remark
1	Positive external control	+	
2	Positive internal control	+	
3	Candida albicans	+	
4	Candida tropicalis	+	
5	Candida parapsilosis	-	
6	Candida krusei	-	
7	Candida glabrata	+	
8	Candida auris	-	
9	Aspergillus fumigatus	-	
10	Aspergillus flavus	-	
11	Aspergillus niger	-	
12	Aspergillus terreus	-	
13	Aspergillus nidulans	-	
14	Aspergillus calidoustu	-	
15	Cryptococcus neoformans	-	
16	Cryptococcus gattii	-	
17	Histoplasma capsulatum	-	
18	Lichtheimia corymbifera	-	
19	Mucor pusillus	-	
20	Trichosporon asahii	-	
21	Pneumocystis jirovecii	-	
22	Penicillium marneffei	-	
23	Negative control	-	
24	Blank	-	

Results Suggest:

Candida albicans,Candida tropicalis and Candida glabrata indexes were positive.

Detector ____________ Auditor ____________ Detection date 2021-03-10 11:10:40

Respiratory fungal nucleic acid test results

Affiliated Hospital Of Guangdong Medical University

Basic sample information

Name		Sexuality	男	Age	58 years old
Section	EICU	Ward		Bed Number	4
ID		Clinical Diagnosis		Sample Types	sputum
Test Items		Chip Number	ZJ-202011010283	Sample Number	2021030903

Detailed Results:

Serial Number	Test Items	Detected Results	Remark
1	Positive external control	+	
2	Positive internal control	+	
3	Candida albicans	-	
4	Candida tropicalis	-	
5	Candida parapsilosis	-	
6	Candida krusei	-	
7	Candida glabrata	-	
8	Candida auris	-	
9	Aspergillus fumigatus	-	
10	Aspergillus flavus	-	
11	Aspergillus niger	-	
12	Aspergillus terreus	-	
13	Aspergillus nidulans	-	
14	Aspergillus calidoustu	-	
15	Cryptococcus neoformans	-	
16	Cryptococcus gattii	-	
17	Histoplasma capsulatum	-	
18	Lichtheimia corymbifera	-	
19	Mucor pusillus	-	
20	Trichosporon asahii	-	
21	Pneumocystis jirovecii	-	
22	Penicillium marneffei	-	
23	Negative control	-	
24	Blank	-	

Results Suggest:

No abnormality was found after detection.

Detector ____________ Auditor ____________ Detection date 2021-03-10 14:19:01

Respiratory fungal nucleic acid test results

Affiliated Hospital Of Guangdong Medical University

Basic sample information

Name		Sexuality	女	Age	51 years old
Section	ICU	Ward		Bed Number	12
ID		Clinical Diagnosis		Sample Types	sputum
Test Items		Chip Number	ZJ-202011010284	Sample Number	2021031501

Detailed Results:

Serial Number	Test Items	Detected Results	Remark
1	Positive external control	+	
2	Positive internal control	+	
3	Candida albicans	+	
4	Candida tropicalis	-	
5	Candida parapsilosis	-	
6	Candida krusei	-	
7	Candida glabrata	-	
8	Candida auris	-	
9	Aspergillus fumigatus	-	
10	Aspergillus flavus	-	
11	Aspergillus niger	-	
12	Aspergillus terreus	-	
13	Aspergillus nidulans	-	
14	Aspergillus calidoustu	-	
15	Cryptococcus neoformans	-	
16	Cryptococcus gattii	-	
17	Histoplasma capsulatum	-	
18	Lichtheimia corymbifera	-	
19	Mucor pusillus	-	
20	Trichosporon asahii	-	
21	Pneumocystis jirovecii	-	
22	Penicillium marneffei	-	
23	Negative control	-	
24	Blank	-	

Results Suggest:

Candida albicans index was positive.

Detector ____________ Auditor ____________ Detection date 2021-03-16 17:25:38

Respiratory fungal nucleic acid test results

Affiliated Hospital Of Guangdong Medical University

Basic sample information

Name		Sexuality	女	Age	69 years old
Section	ICU	Ward		Bed Number	206
ID		Clinical Diagnosis		Sample Types	sputum
Test Items		Chip Number	ZJ-202011010285	Sample Number	2021031502

Detailed Results:

Serial Number	Test Items	Detected Results	Remark
1	Positive external control	+	
2	Positive internal control	+	
3	Candida albicans	-	
4	Candida tropicalis	-	
5	Candida parapsilosis	-	
6	Candida krusei	-	
7	Candida glabrata	-	
8	Candida auris	-	
9	Aspergillus fumigatus	-	
10	Aspergillus flavus	-	
11	Aspergillus niger	-	
12	Aspergillus terreus	-	
13	Aspergillus nidulans	-	
14	Aspergillus calidoustu	-	
15	Cryptococcus neoformans	-	
16	Cryptococcus gattii	-	
17	Histoplasma capsulatum	-	
18	Lichtheimia corymbifera	-	
19	Mucor pusillus	-	
20	Trichosporon asahii	-	
21	Pneumocystis jirovecii	-	
22	Penicillium marneffei	-	
23	Negative control	-	
24	Blank	-	

Results Suggest:

No abnormality was found after detection.

Detector ____________ Auditor ____________ Detection date 2021-03-16 18:28:42

Respiratory fungal nucleic acid test results

Affiliated Hospital Of Guangdong Medical University

Basic sample information

Name		Sexuality	男	Age	49 years old
Section	ICU	Ward		Bed Number	307
ID		Clinical Diagnosis		Sample Types	sputum
Test Items		Chip Number	ZJ-202011010286	Sample Number	2021031504

Detailed Results:

Serial Number	Test Items	Detected Results	Remark
1	Positive external control	+	
2	Positive internal control	+	
3	Candida albicans	-	
4	Candida tropicalis	-	
5	Candida parapsilosis	-	
6	Candida krusei	-	
7	Candida glabrata	+	
8	Candida auris	-	
9	Aspergillus fumigatus	-	
10	Aspergillus flavus	-	
11	Aspergillus niger	-	
12	Aspergillus terreus	-	
13	Aspergillus nidulans	-	
14	Aspergillus calidoustu	-	
15	Cryptococcus neoformans	-	
16	Cryptococcus gattii	-	
17	Histoplasma capsulatum	-	
18	Lichtheimia corymbifera	-	
19	Mucor pusillus	-	
20	Trichosporon asahii	-	
21	Pneumocystis jirovecii	-	
22	Penicillium marneffei	-	
23	Negative control	-	
24	Blank	-	

Results Suggest:

Candida glabrata index was positive.

Detector ____________ Auditor ____________ Detection date 2021-03-16 19:23:05

Respiratory fungal nucleic acid test results

Affiliated Hospital Of Guangdong Medical University

Basic sample information

Name		Sexuality	男	Age	46 years old
Section	ICU	Ward		Bed Number	310
ID		Clinical Diagnosis		Sample Types	sputum
Test Items		Chip Number	ZJ-202011010287	Sample Number	2021031505

Detailed Results:

Serial Number	Test Items	Detected Results	Remark
1	Positive external control	+	
2	Positive internal control	+	
3	Candida albicans	-	
4	Candida tropicalis	-	
5	Candida parapsilosis	-	
6	Candida krusei	-	
7	Candida glabrata	-	
8	Candida auris	-	
9	Aspergillus fumigatus	-	
10	Aspergillus flavus	-	
11	Aspergillus niger	-	
12	Aspergillus terreus	-	
13	Aspergillus nidulans	-	
14	Aspergillus calidoustu	-	
15	Cryptococcus neoformans	-	
16	Cryptococcus gattii	-	
17	Histoplasma capsulatum	-	
18	Lichtheimia corymbifera	-	
19	Mucor pusillus	-	
20	Trichosporon asahii	-	
21	Pneumocystis jirovecii	-	
22	Penicillium marneffei	-	
23	Negative control	-	
24	Blank	-	

Results Suggest:

No abnormality was found after detection.

Detector ____________ Auditor ____________ Detection date 2021-03-17 08:49:46

Respiratory fungal nucleic acid test results

Affiliated Hospital Of Guangdong Medical University

Basic sample information

Name		Sexuality	男	Age	37 years old
Section	department of respiratory medicine	Ward		Bed Number	3001
ID		Clinical Diagnosis		Sample Types	bronchoalveolar lavage fluid
Test Items		Chip Number	ZJ-202011010289	Sample Number	2021060101

Detailed Results:

Serial Number	Test Items	Detected Results	Remark
1	Positive external control	+	
2	Positive internal control	+	
3	Candida albicans	+	
4	Candida tropicalis	-	
5	Candida parapsilosis	-	
6	Candida krusei	-	
7	Candida glabrata	-	
8	Candida auris	-	
9	Aspergillus fumigatus	-	
10	Aspergillus flavus	-	
11	Aspergillus niger	-	
12	Aspergillus terreus	-	
13	Aspergillus nidulans	-	
14	Aspergillus calidoustu	-	
15	Cryptococcus neoformans	-	
16	Cryptococcus gattii	-	
17	Histoplasma capsulatum	-	
18	Lichtheimia corymbifera	-	
19	Mucor pusillus	-	
20	Trichosporon asahii	-	
21	Pneumocystis jirovecii	-	
22	Penicillium marneffei	-	
23	Negative control	-	
24	Blank	-	

Results Suggest:

Candida albicans index was positive.

Detector ____________ Auditor ____________ Detection date 2023-06-25 21:06:07

Algorithm Processing Curve

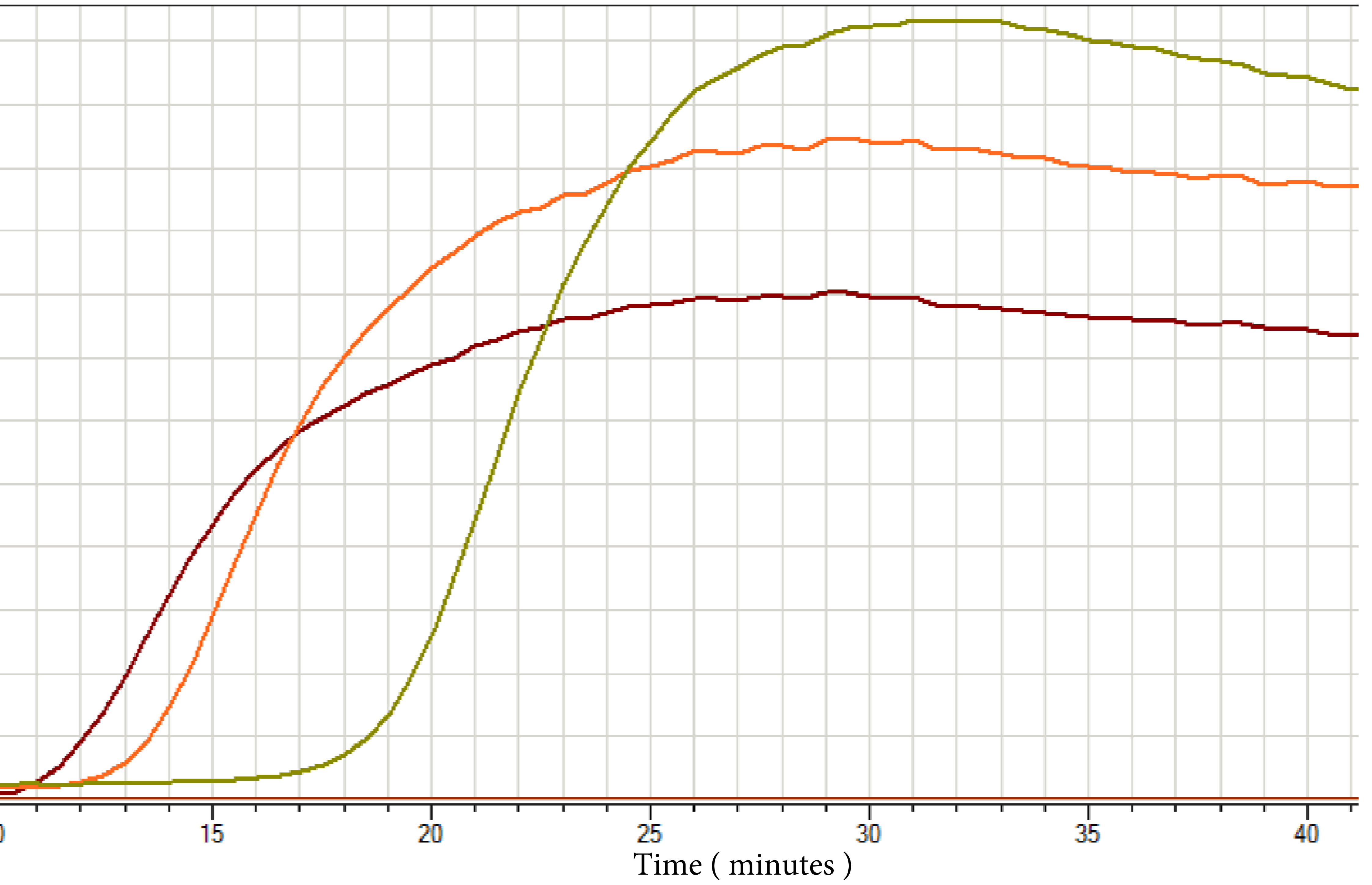

Algorithm Processing Curve

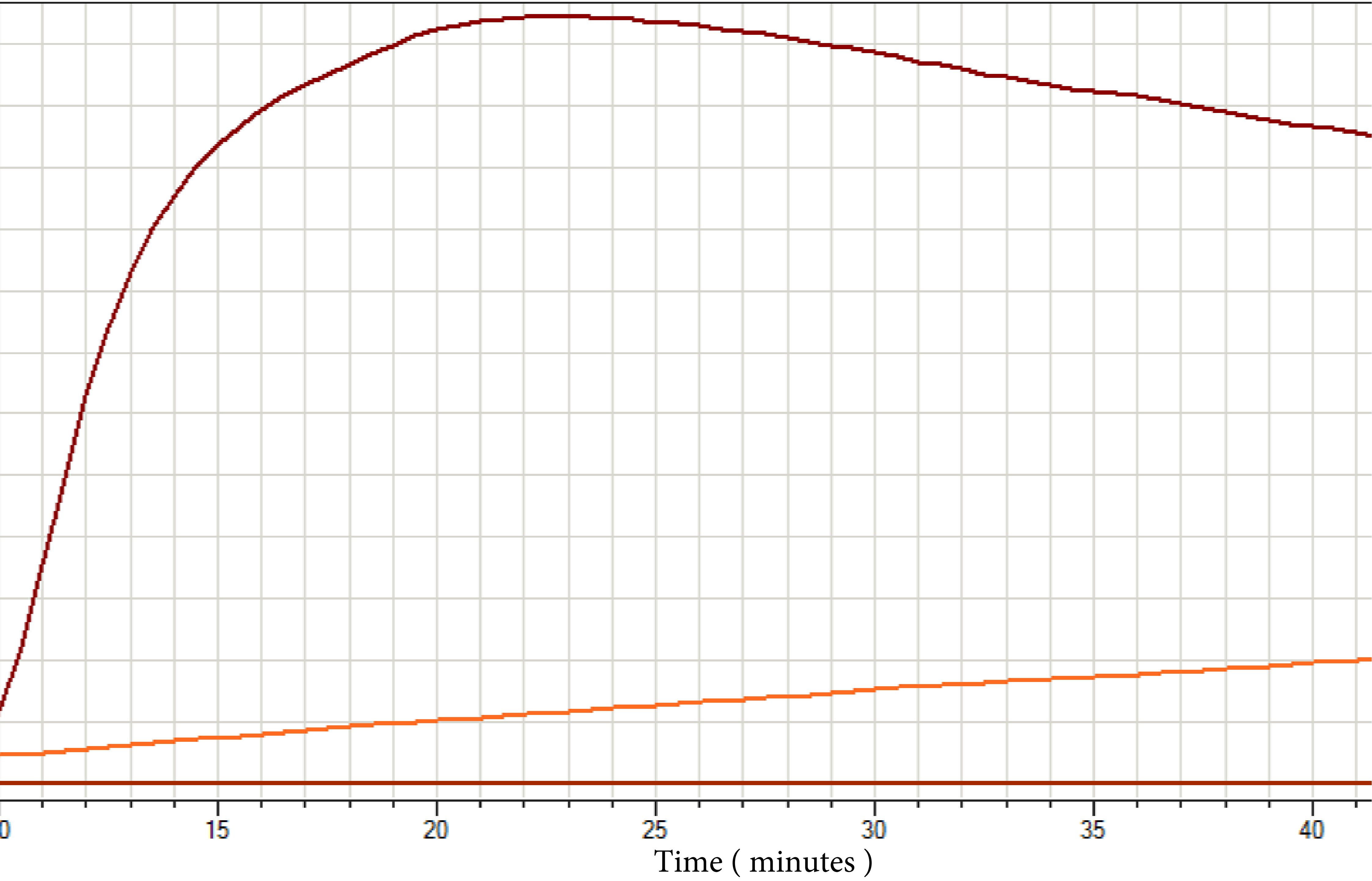

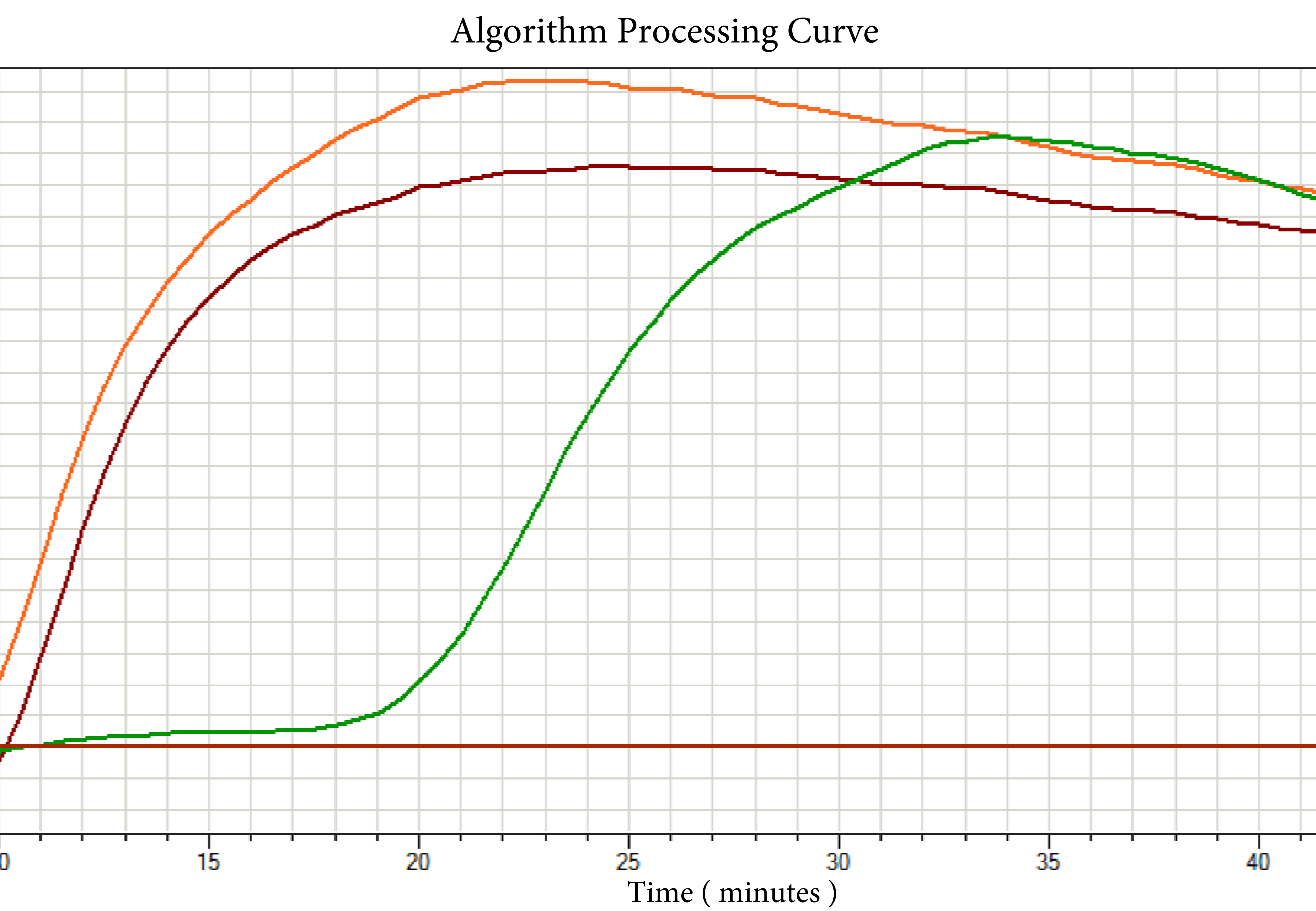
Algorithm Processing Curve
0
15
20
25
30
35
40
Time (minutes)

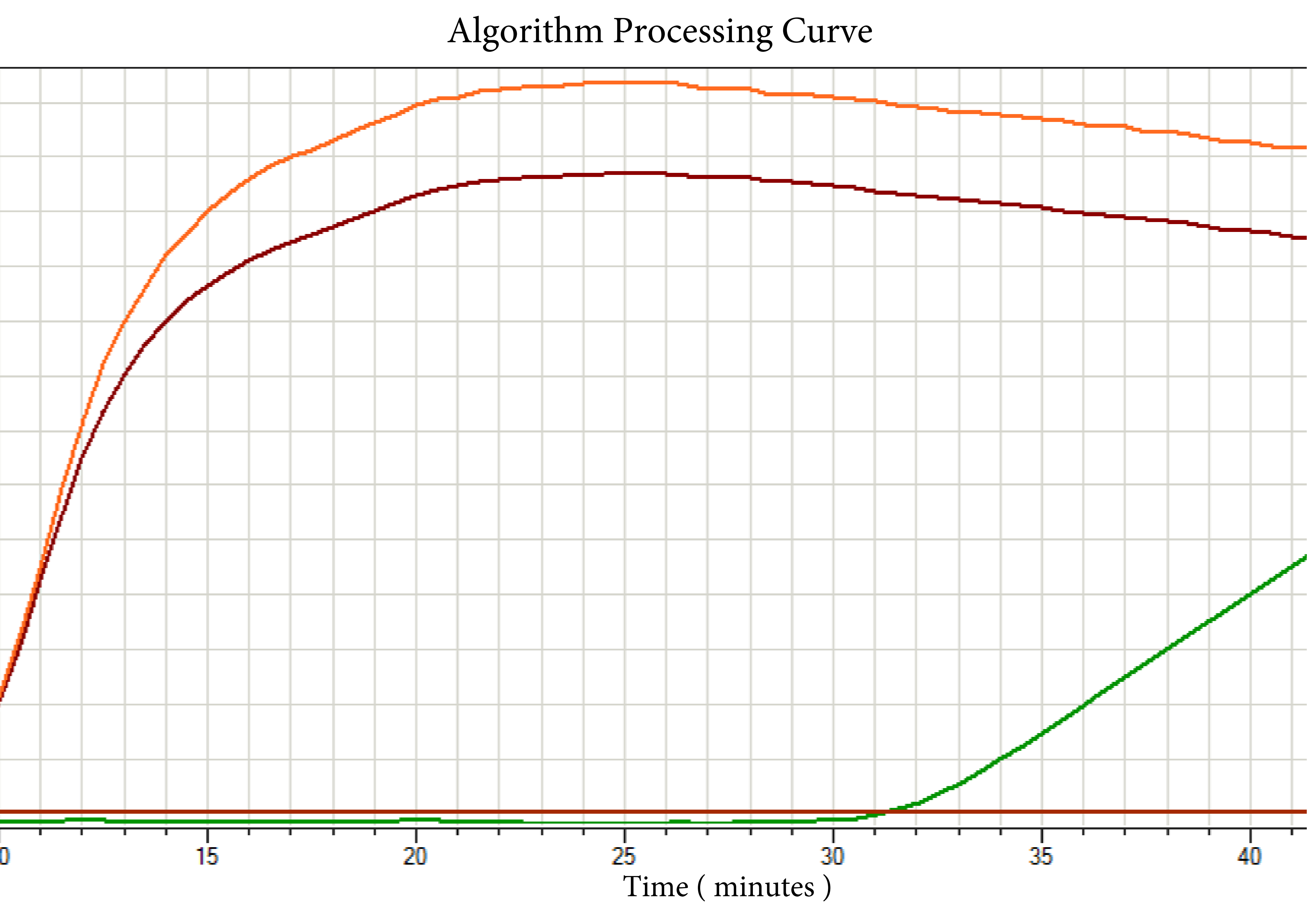
Algorithm Processing Curve
0
15
20
25
30
35
40
Time (minutes)

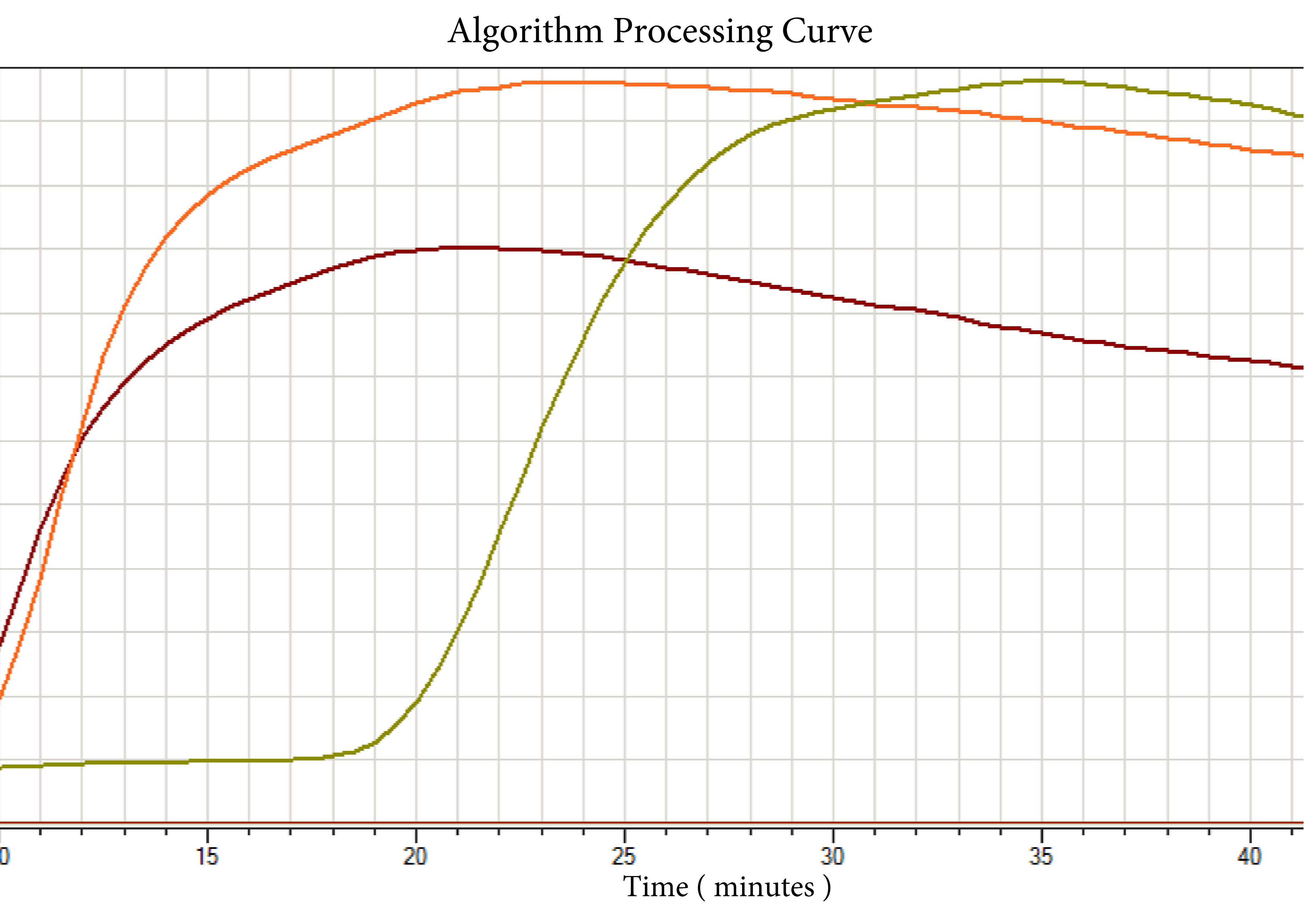

Algorithm Processing Curve
0
15
20
25
30
35
40
Time (minutes)

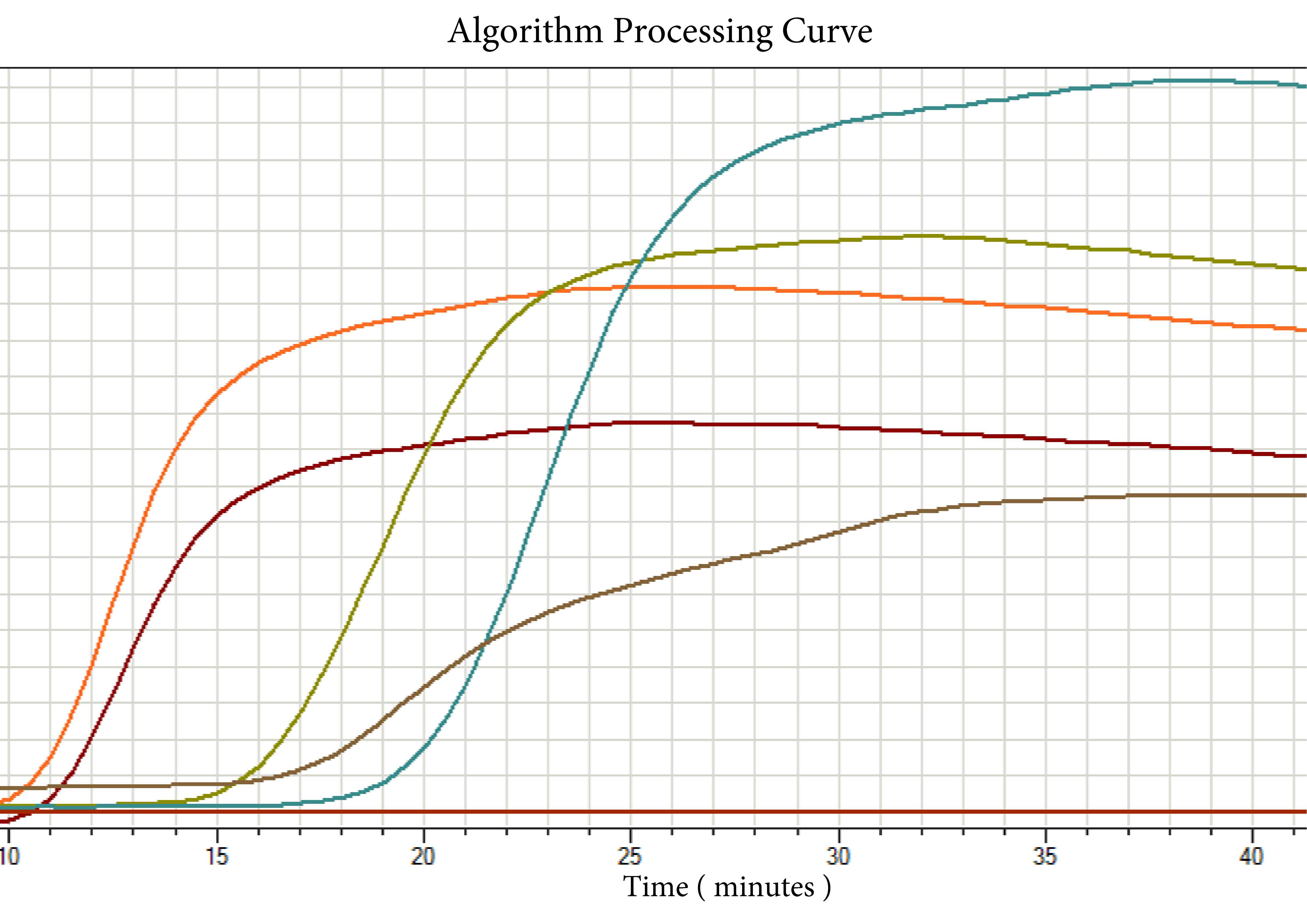

Algorithm Processing Curve
10
15
20
25
30
35
40
Time (minutes)

Algorithm Processing Curve

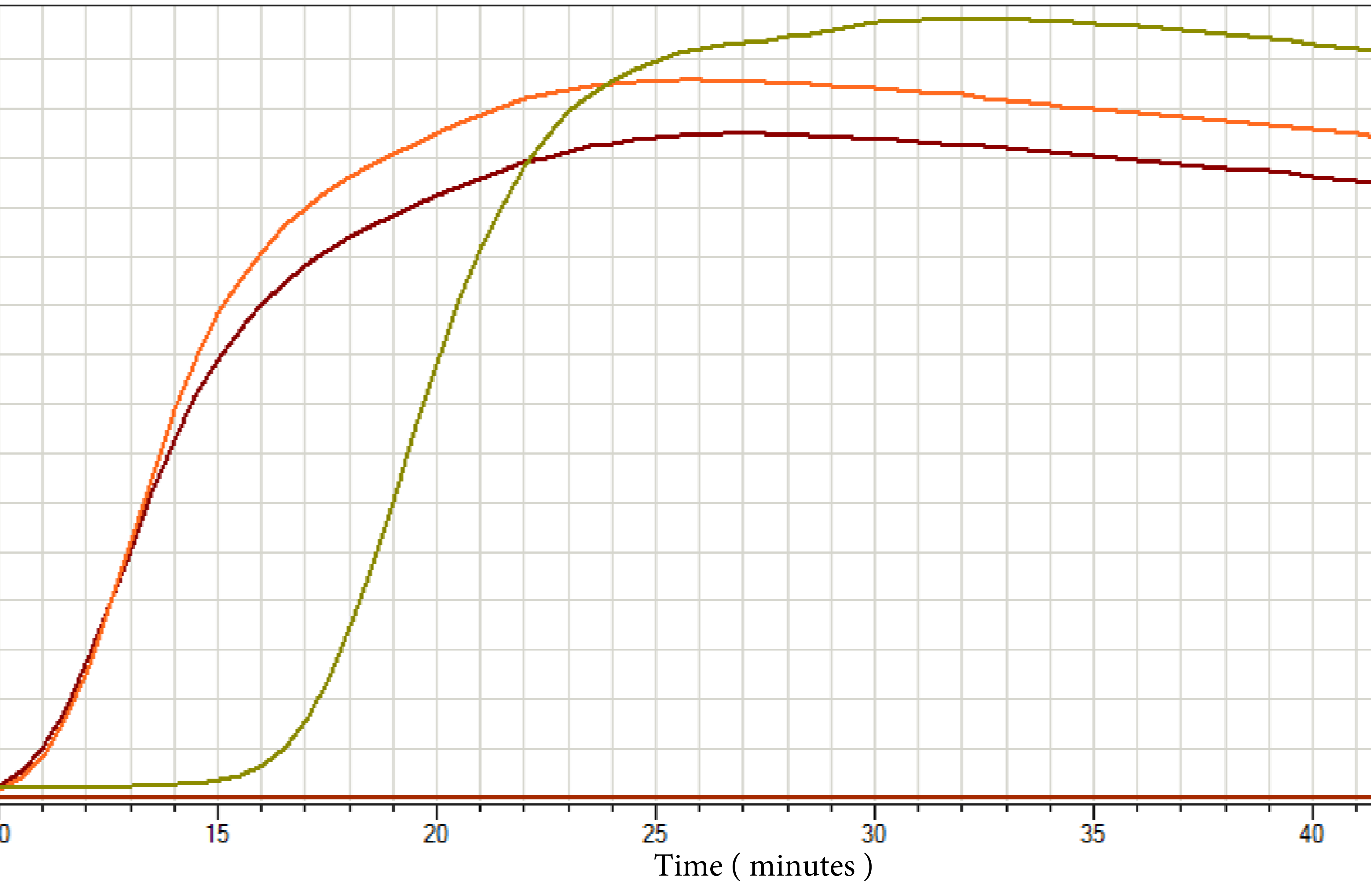

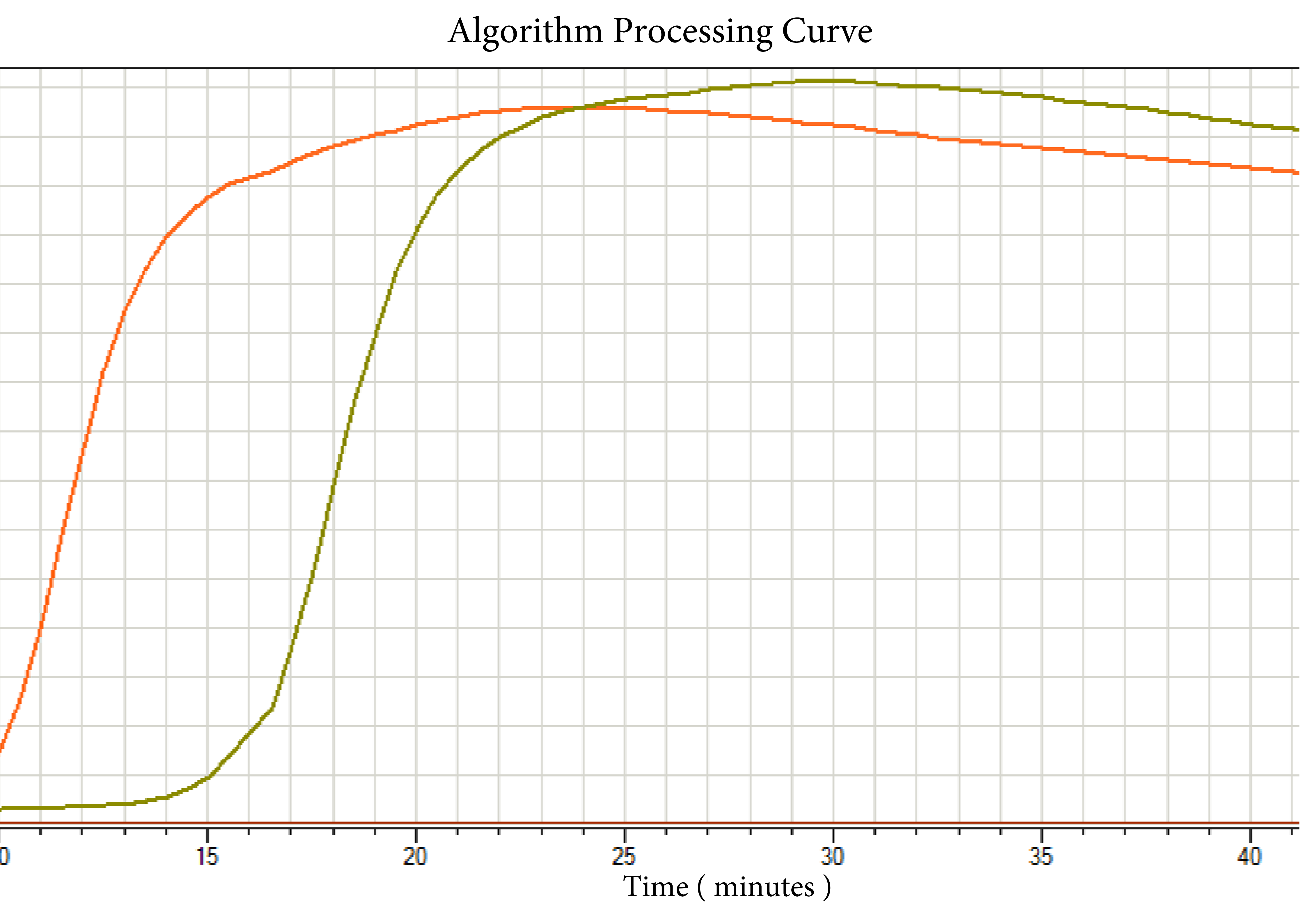

Algorithm Processing Curve
0
15
20
25
30
35
40
Time (minutes)

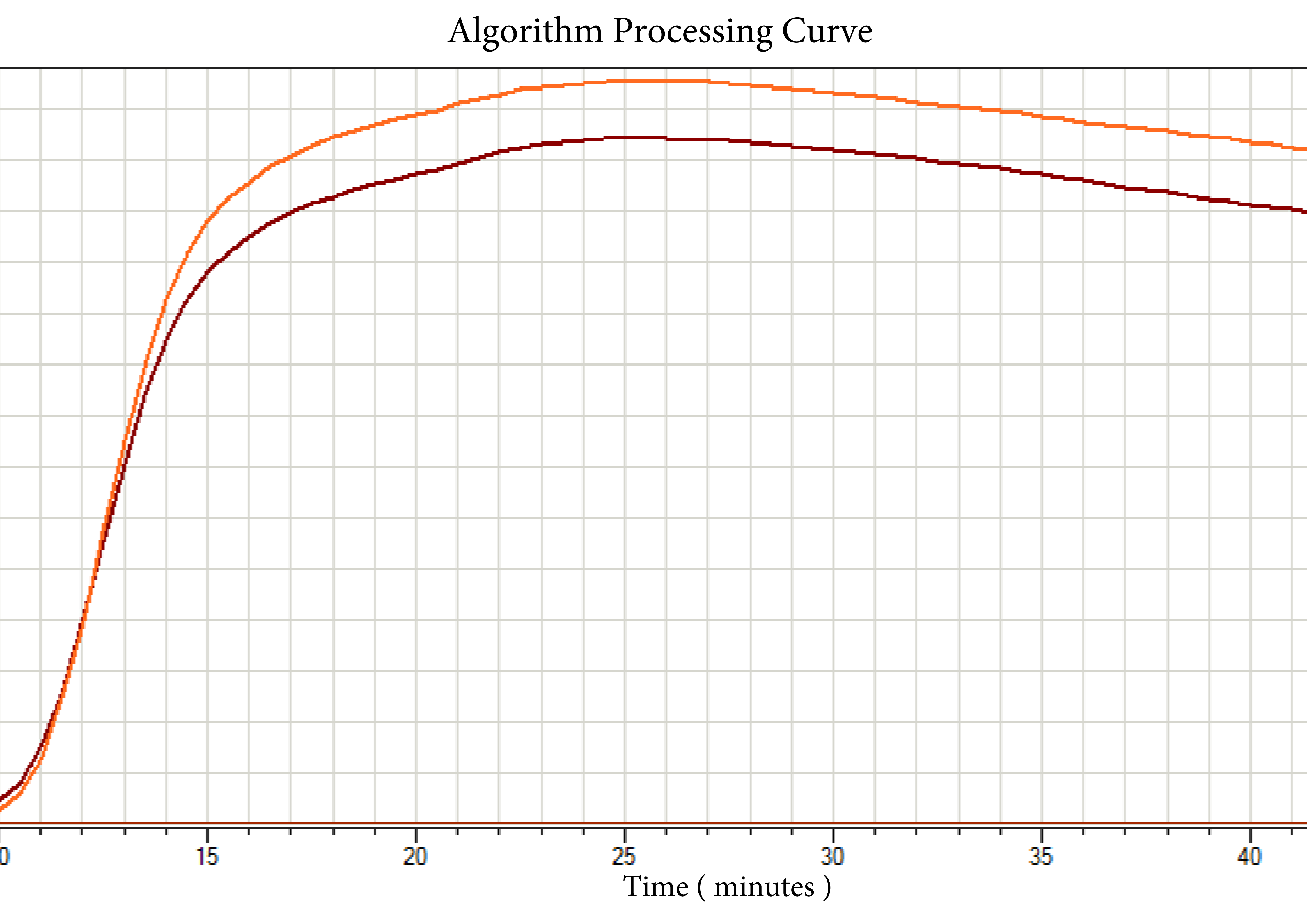

Algorithm Processing Curve
0
15
20
25
30
35
40
Time (minutes)

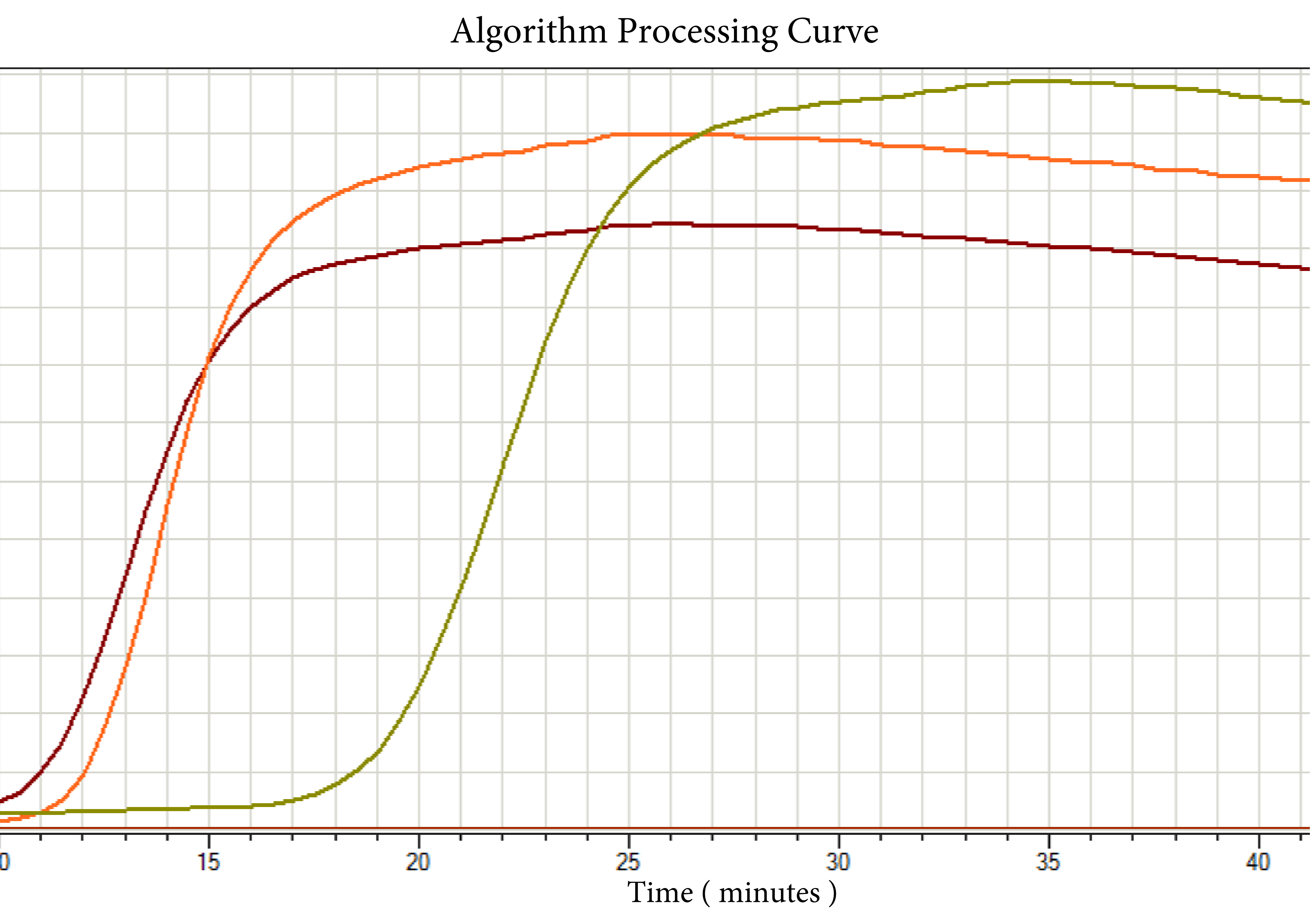
Algorithm Processing Curve
0
15
20
25
30
35
40
Time (minutes)

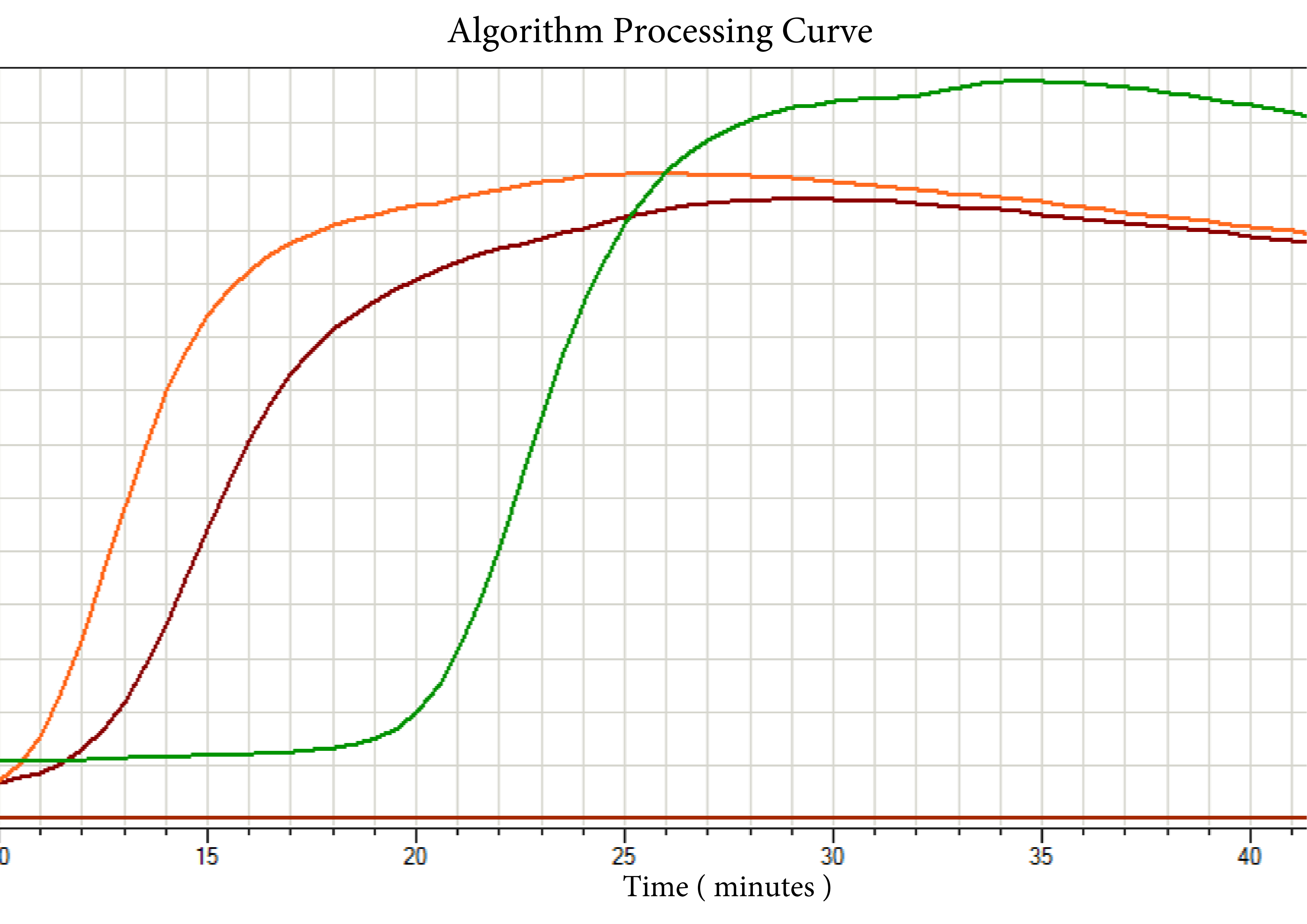
Algorithm Processing Curve
0
15
20
25
30
35
40
Time (minutes)

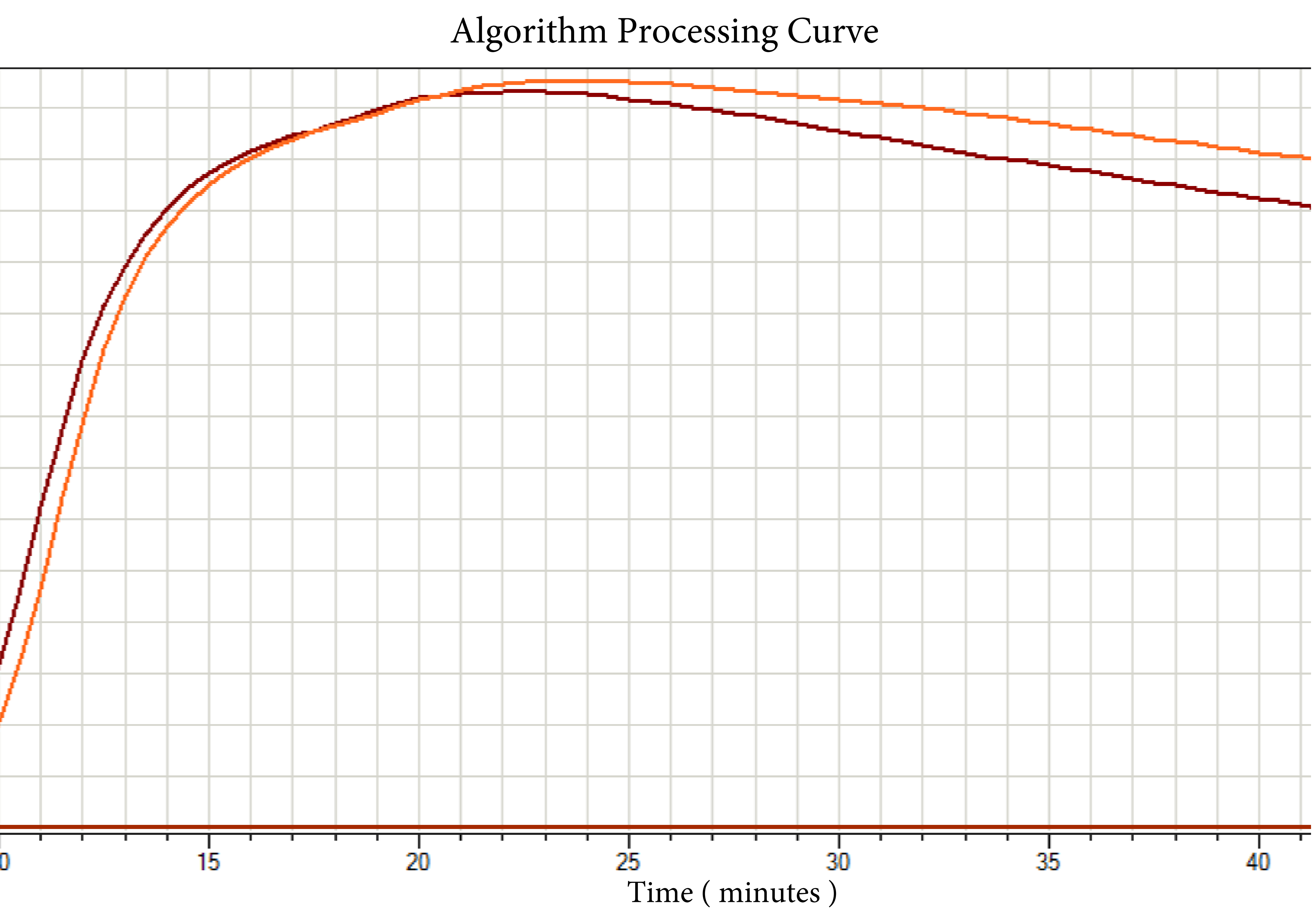
Algorithm Processing Curve
0
15
20
25
30
35
40
Time (minutes)

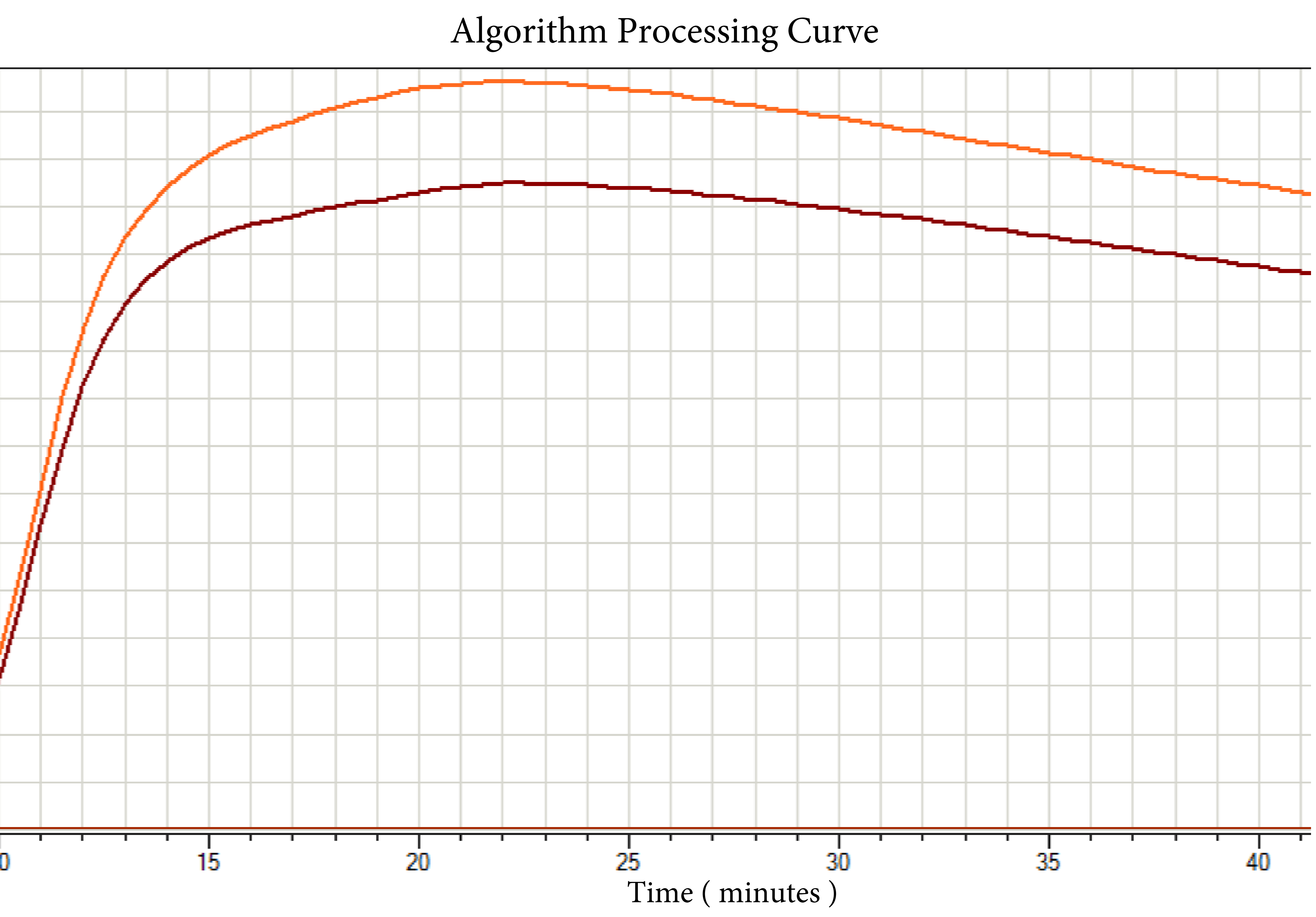

Algorithm Processing Curve
0
15
20
25
30
35
40
Time (minutes)

Algorithm Processing Curve
0
15
20
25
30
35
40
Time (minutes)
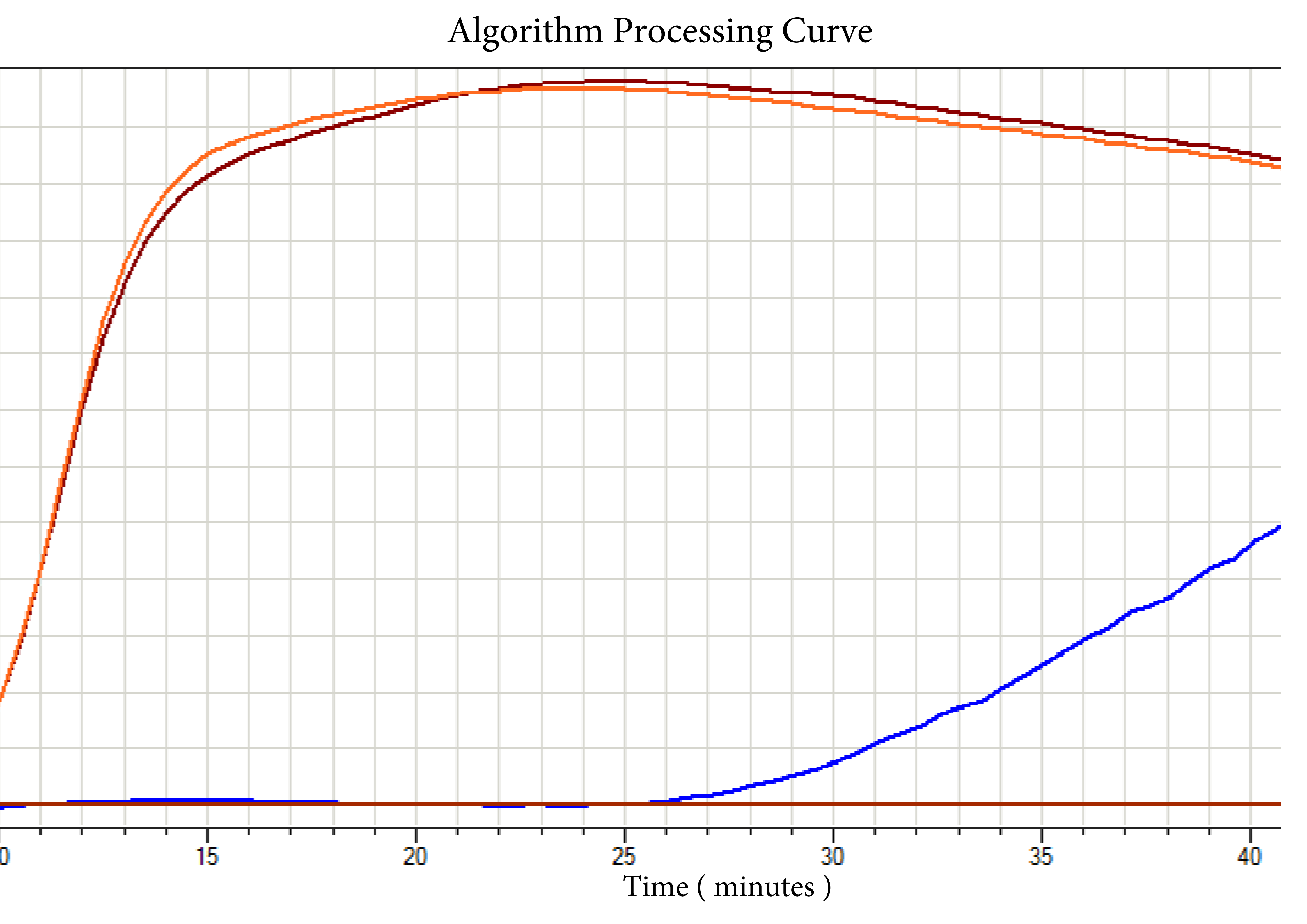

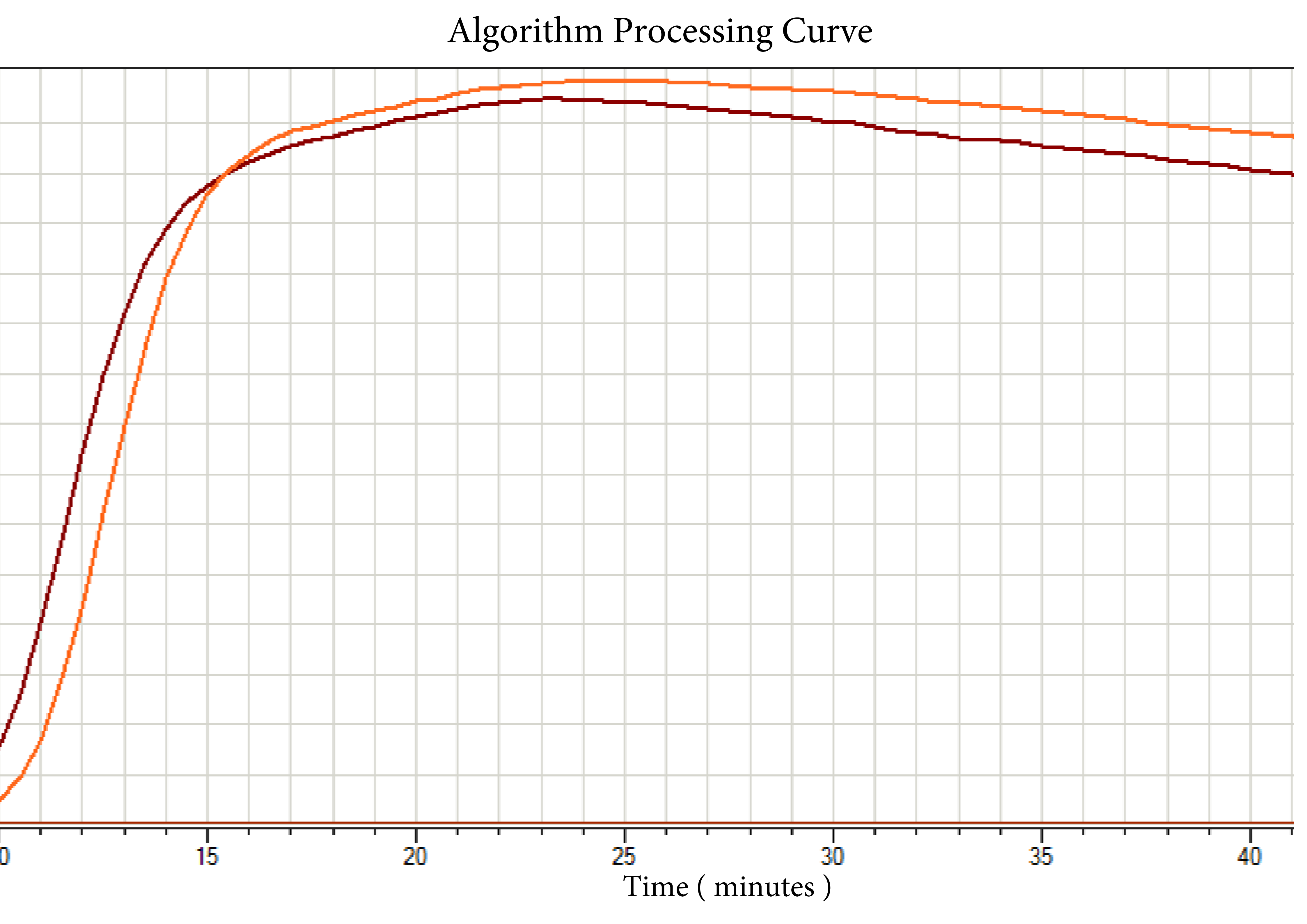
Algorithm Processing Curve
0
15
20
25
30
35
40
Time (minutes)

Algorithm Processing Curve

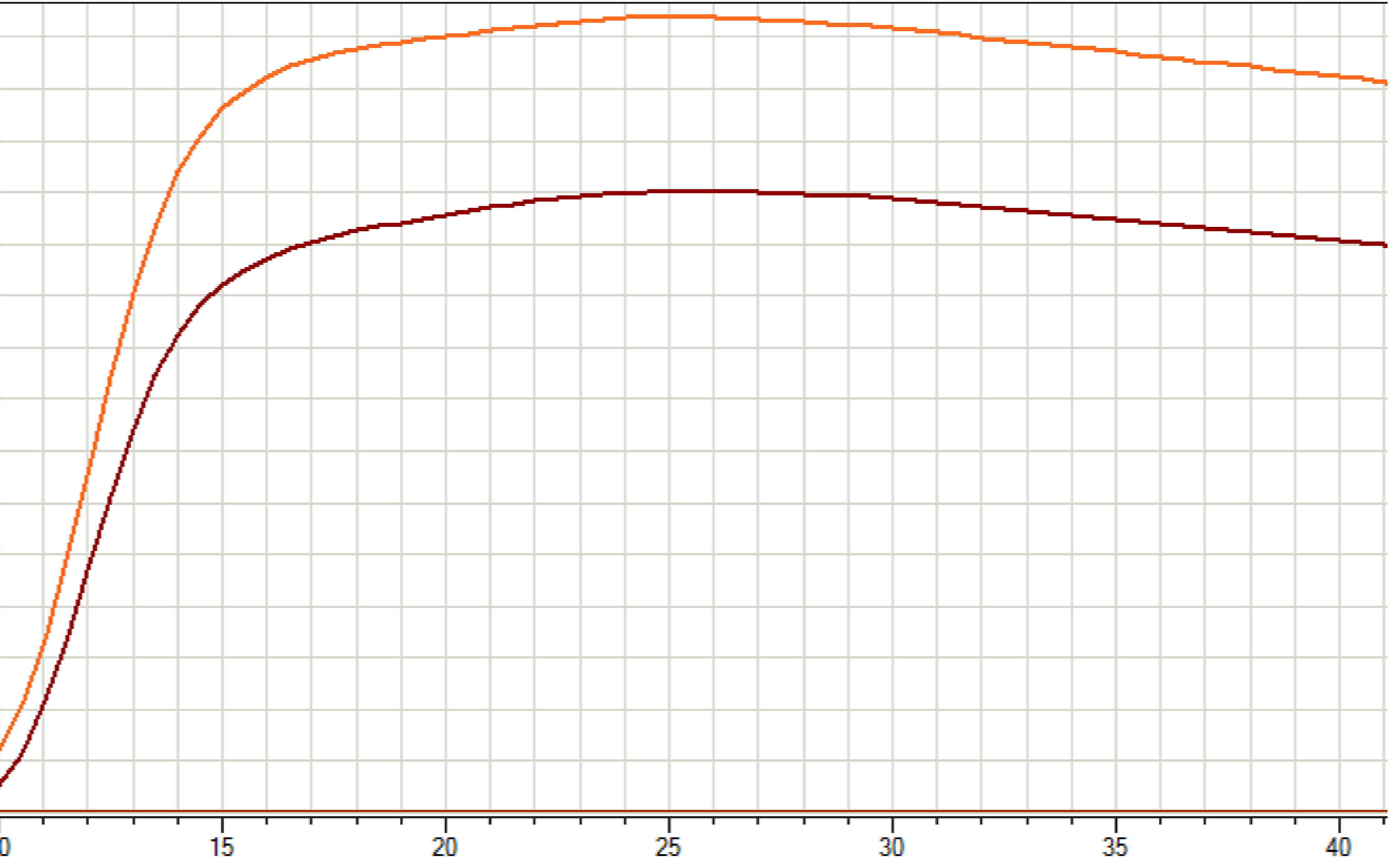

Algorithm Processing Curve

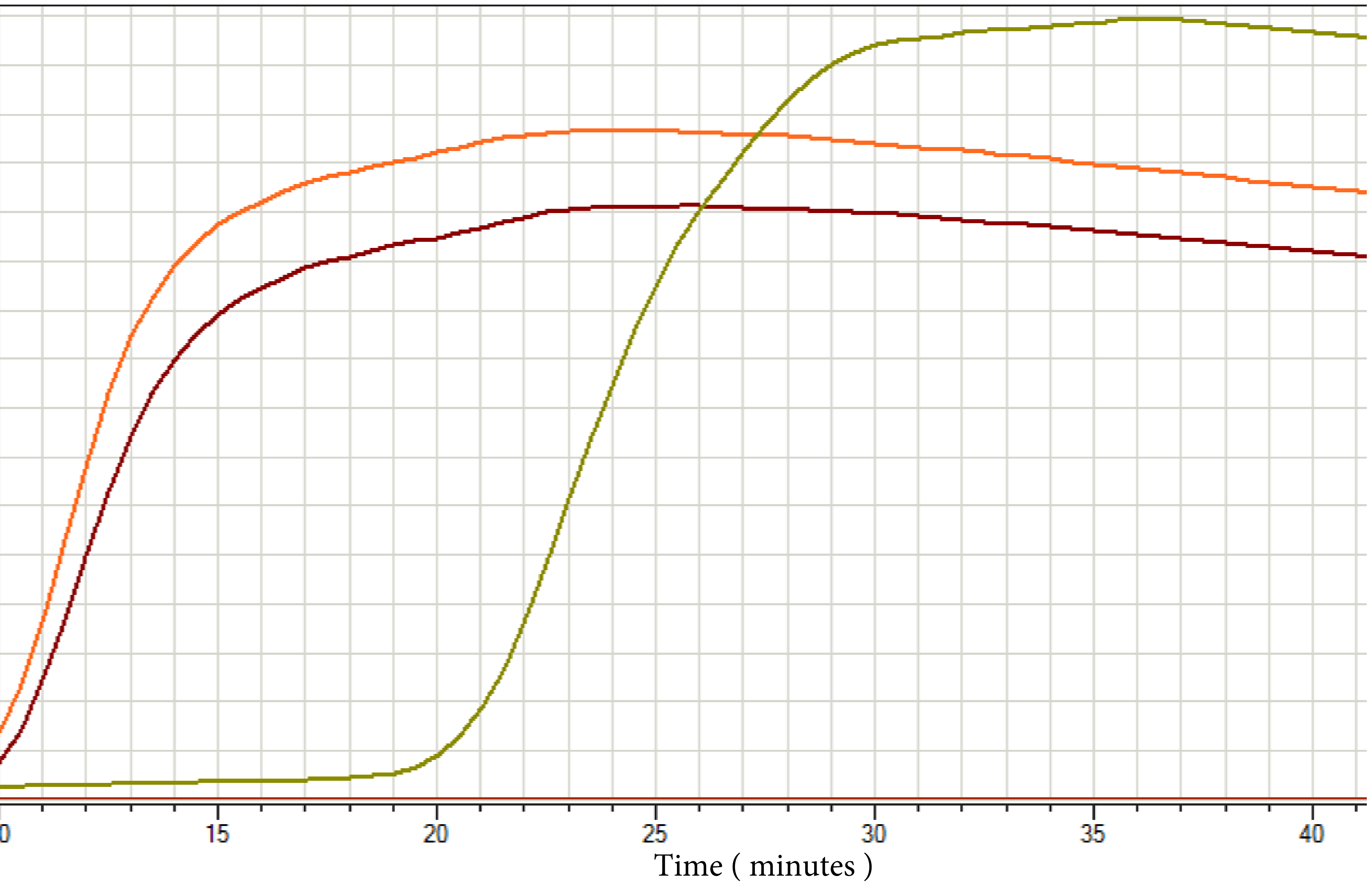

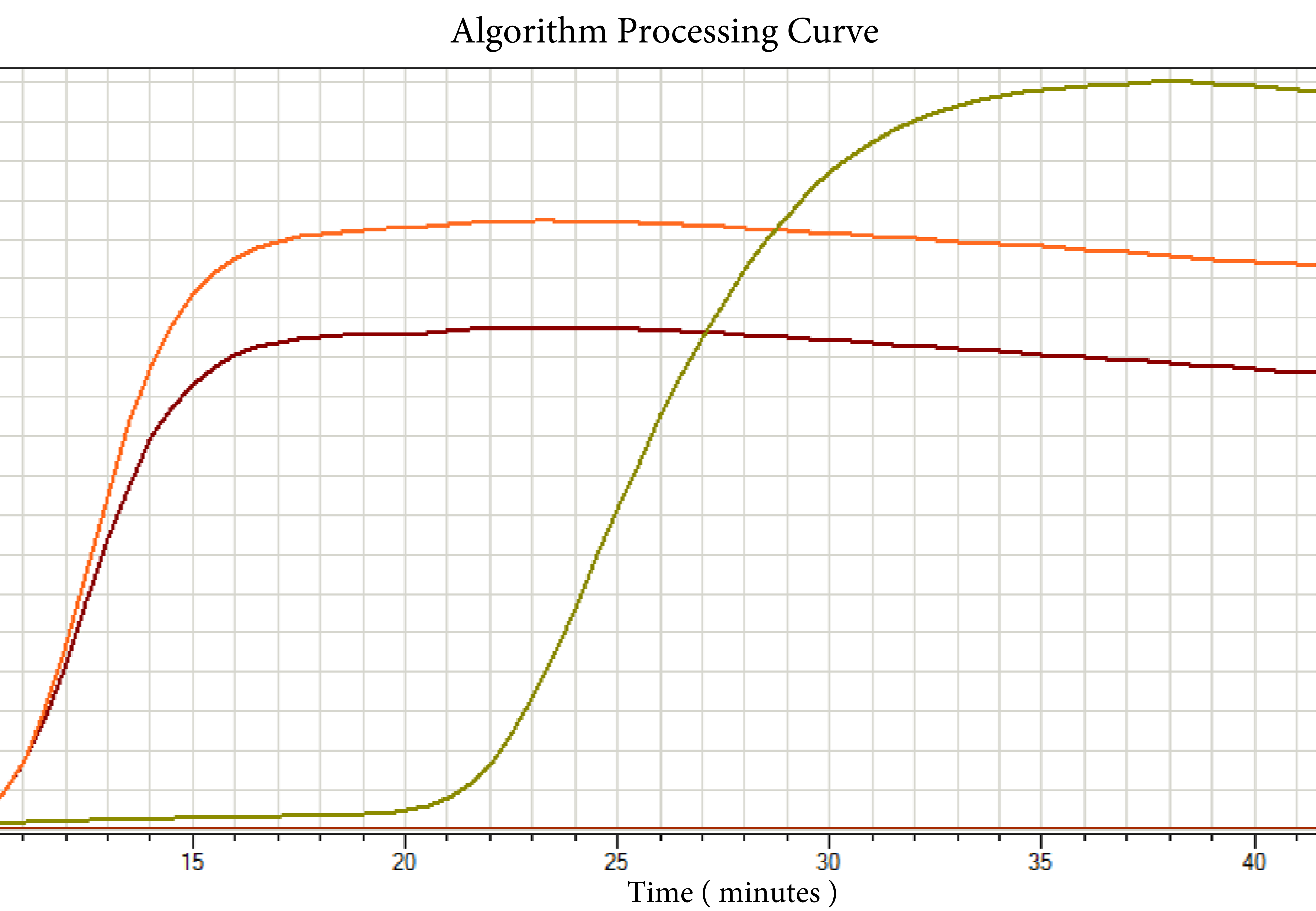
Algorithm Processing Curve
15
20
25
30
35
40
Time (minutes)

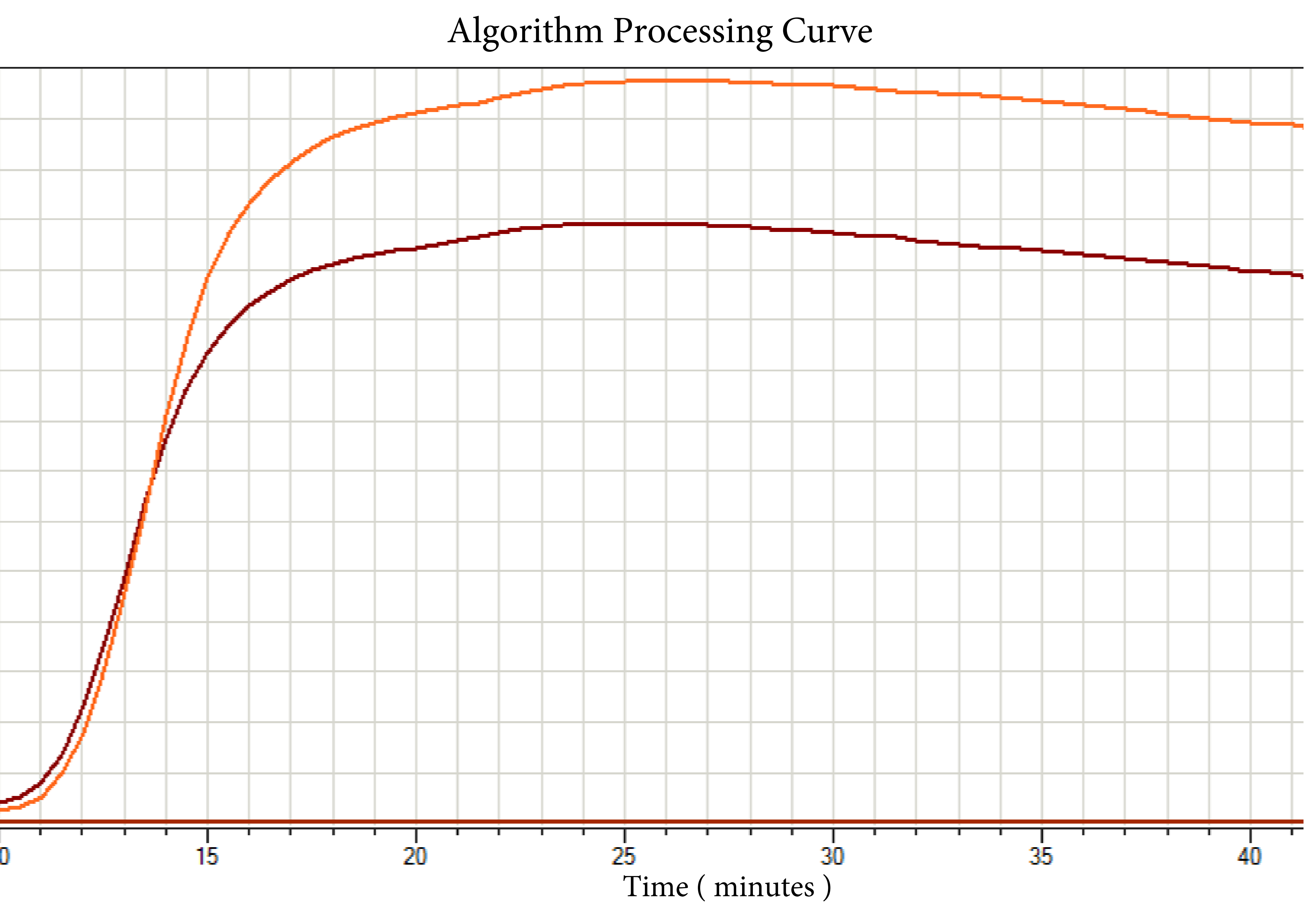
Algorithm Processing Curve
0
15
20
25
30
35
40
Time (minutes)

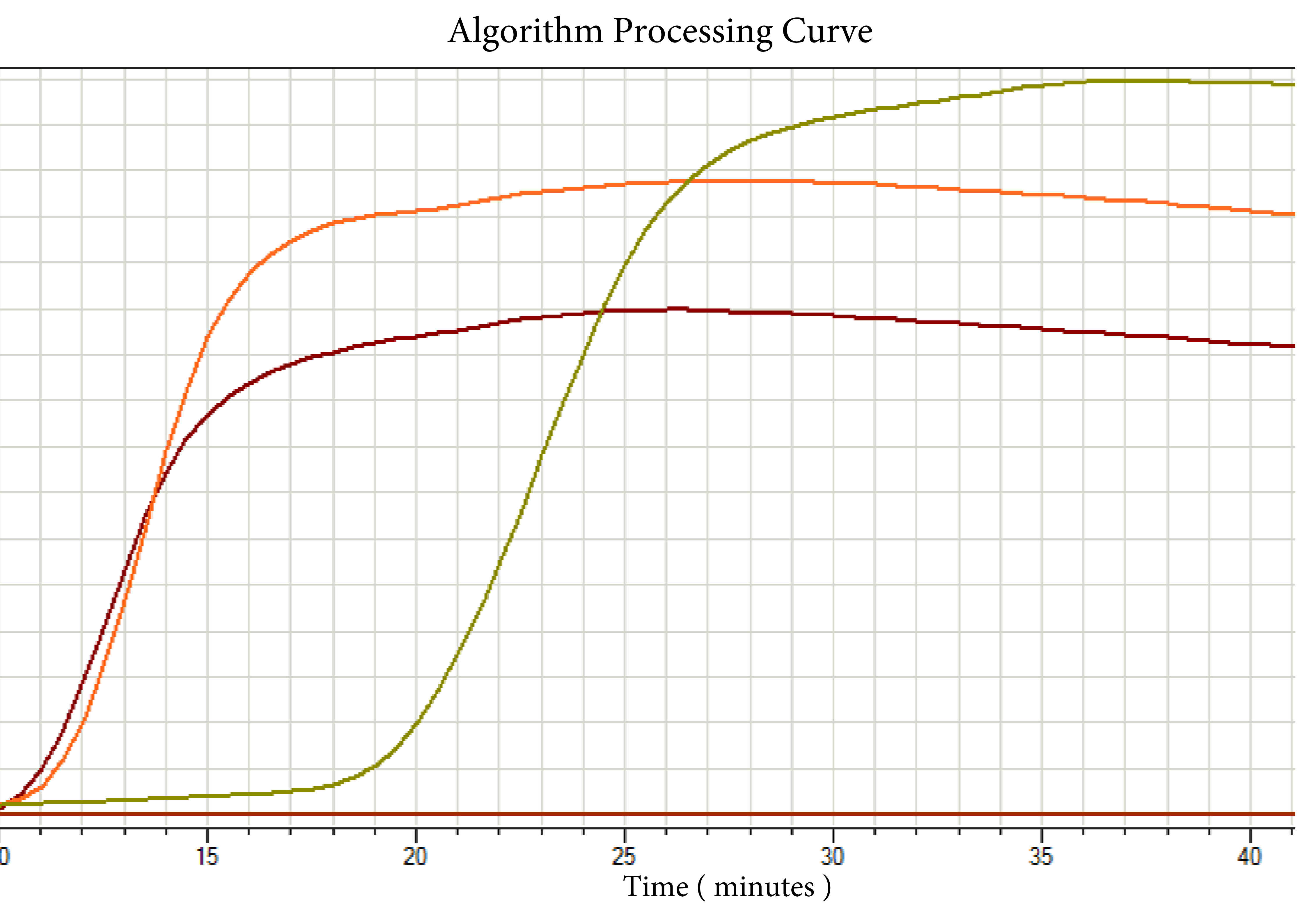
Algorithm Processing Curve
0
15
20
25
30
35
40
Time (minutes)

Algorithm Processing Curve

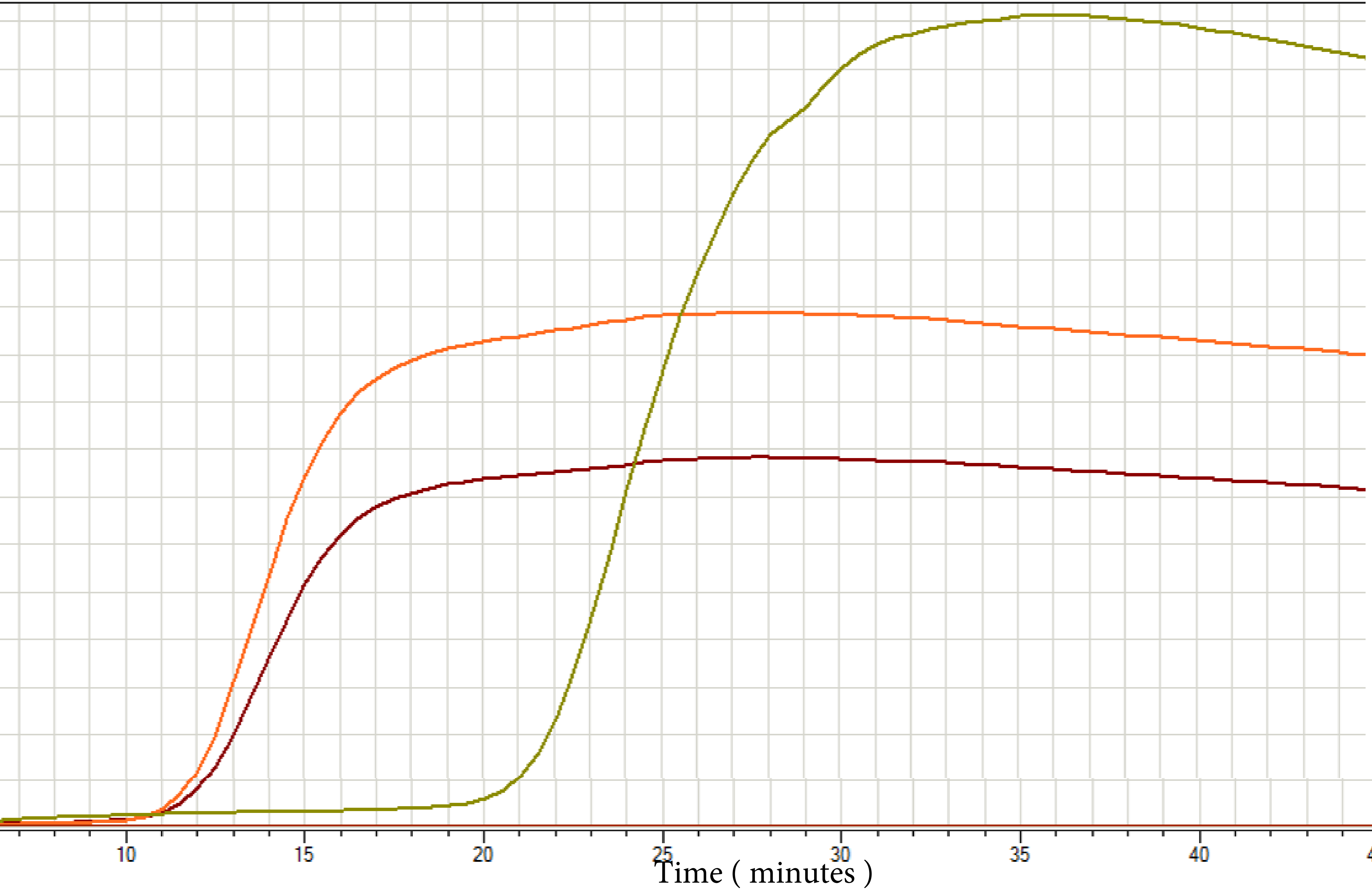

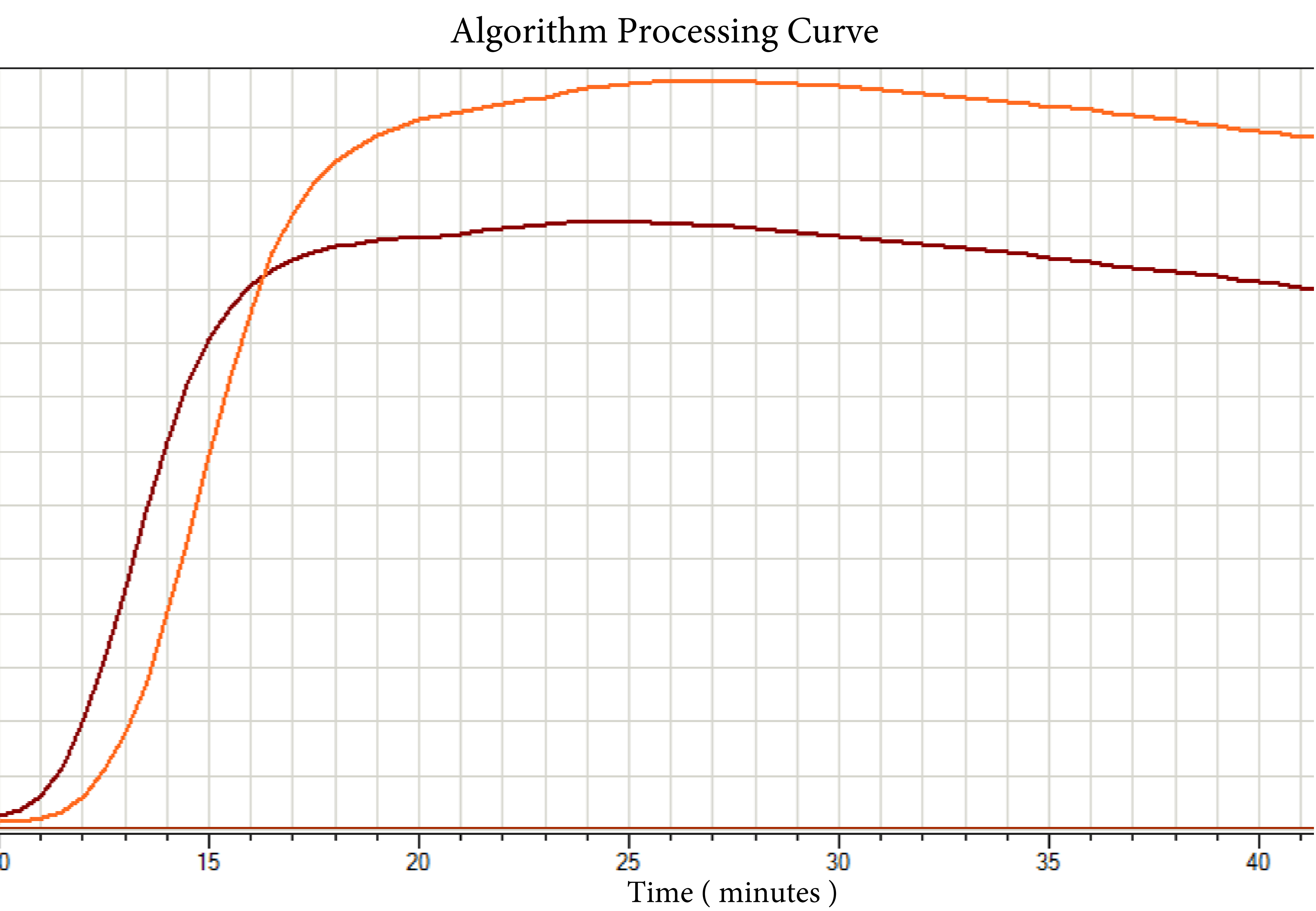
Algorithm Processing Curve
0
15
20
25
30
35
40
Time (minutes)

Algorithm Processing Curve

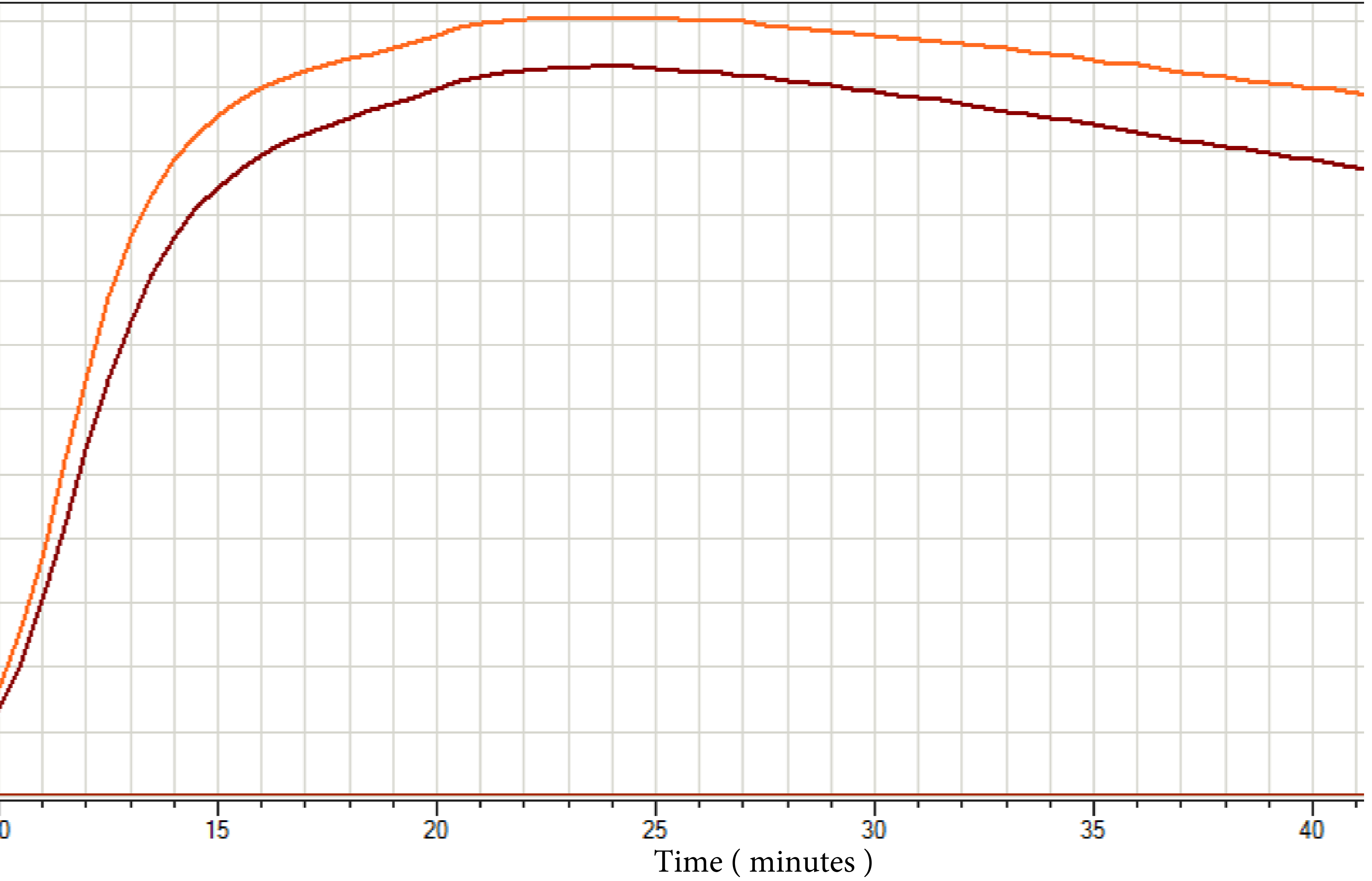

Algorithm Processing Curve

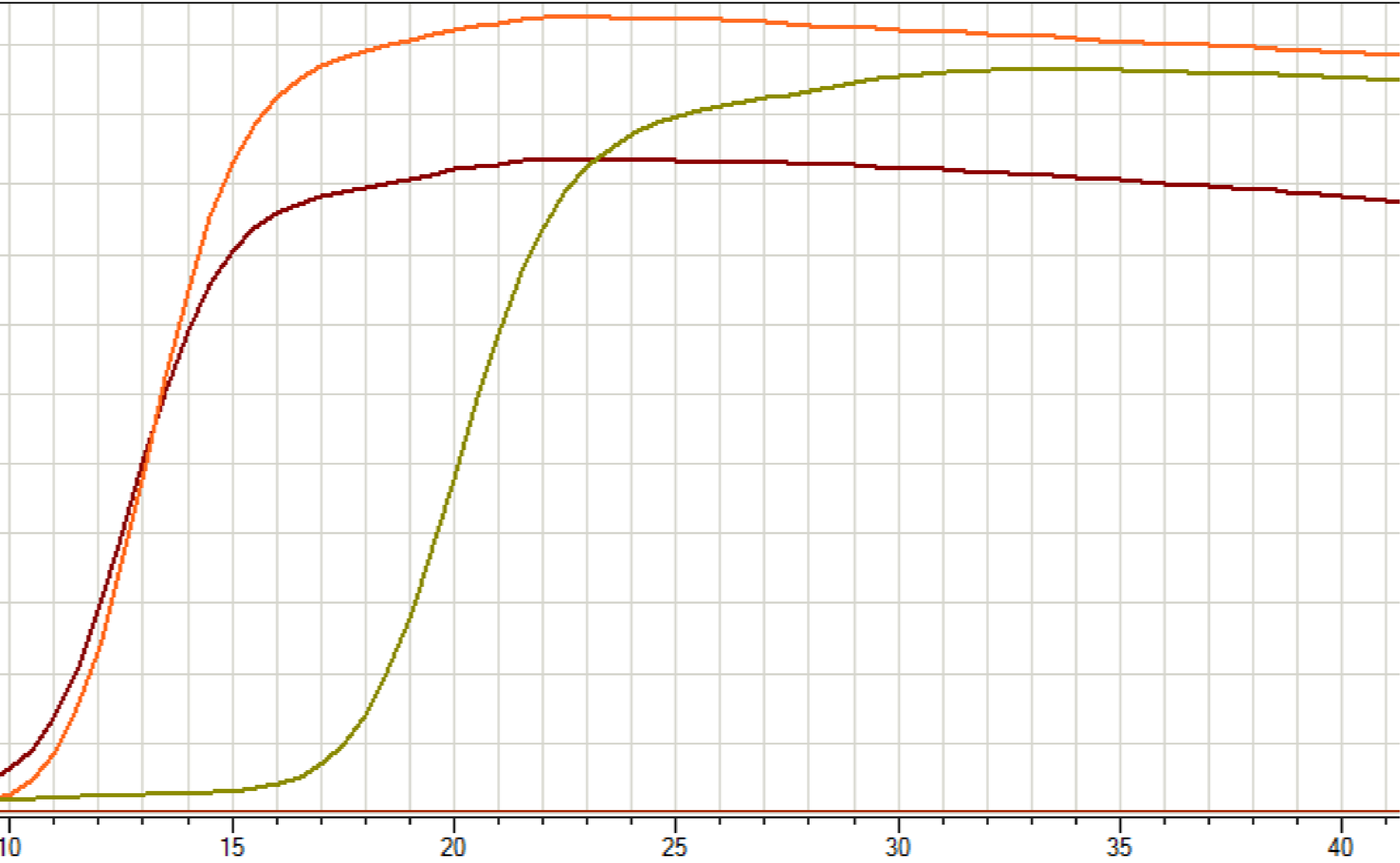

Algorithm Processing Curve

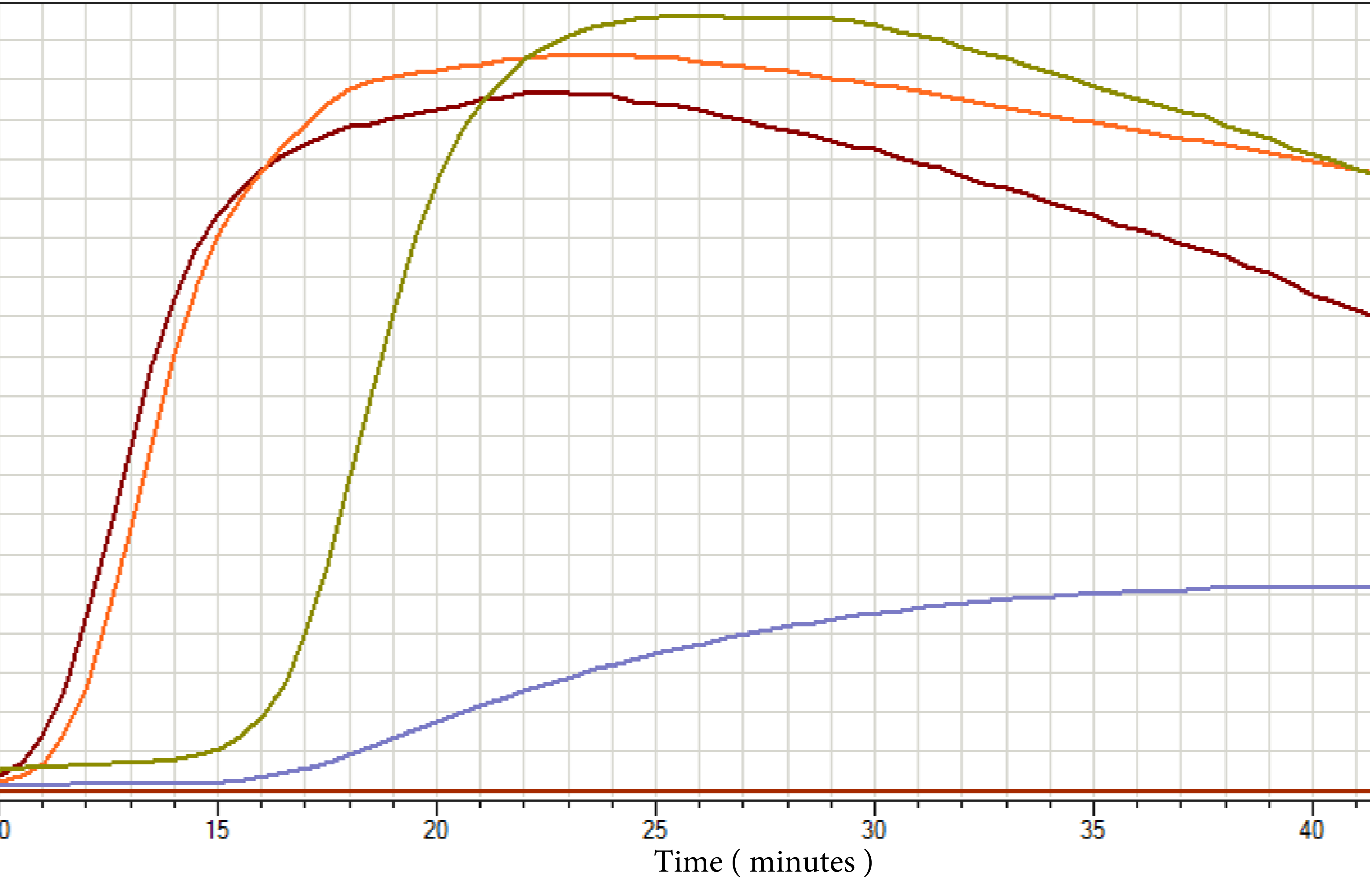

Algorithm Processing Curve

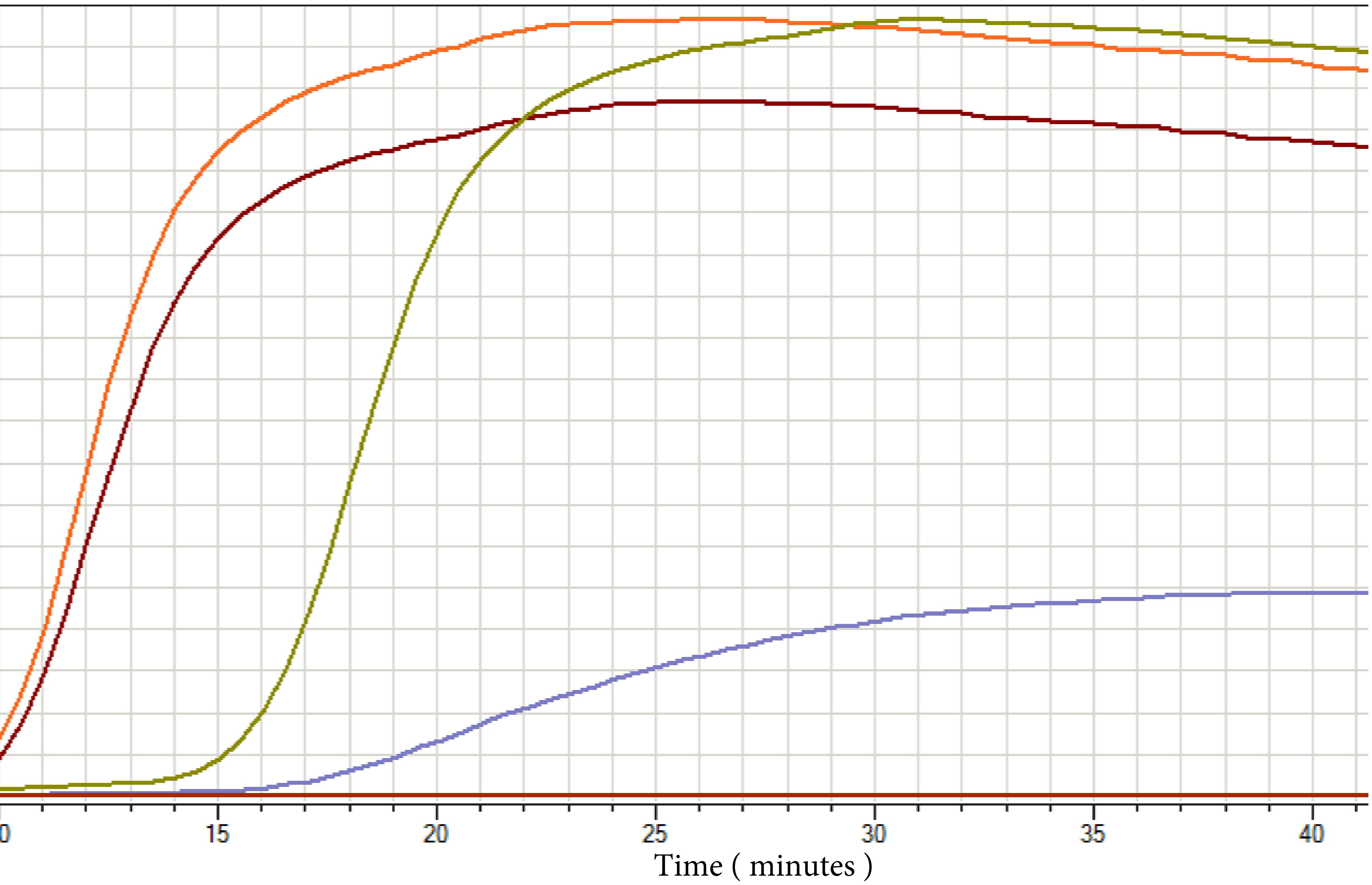

Algorithm Processing Curve

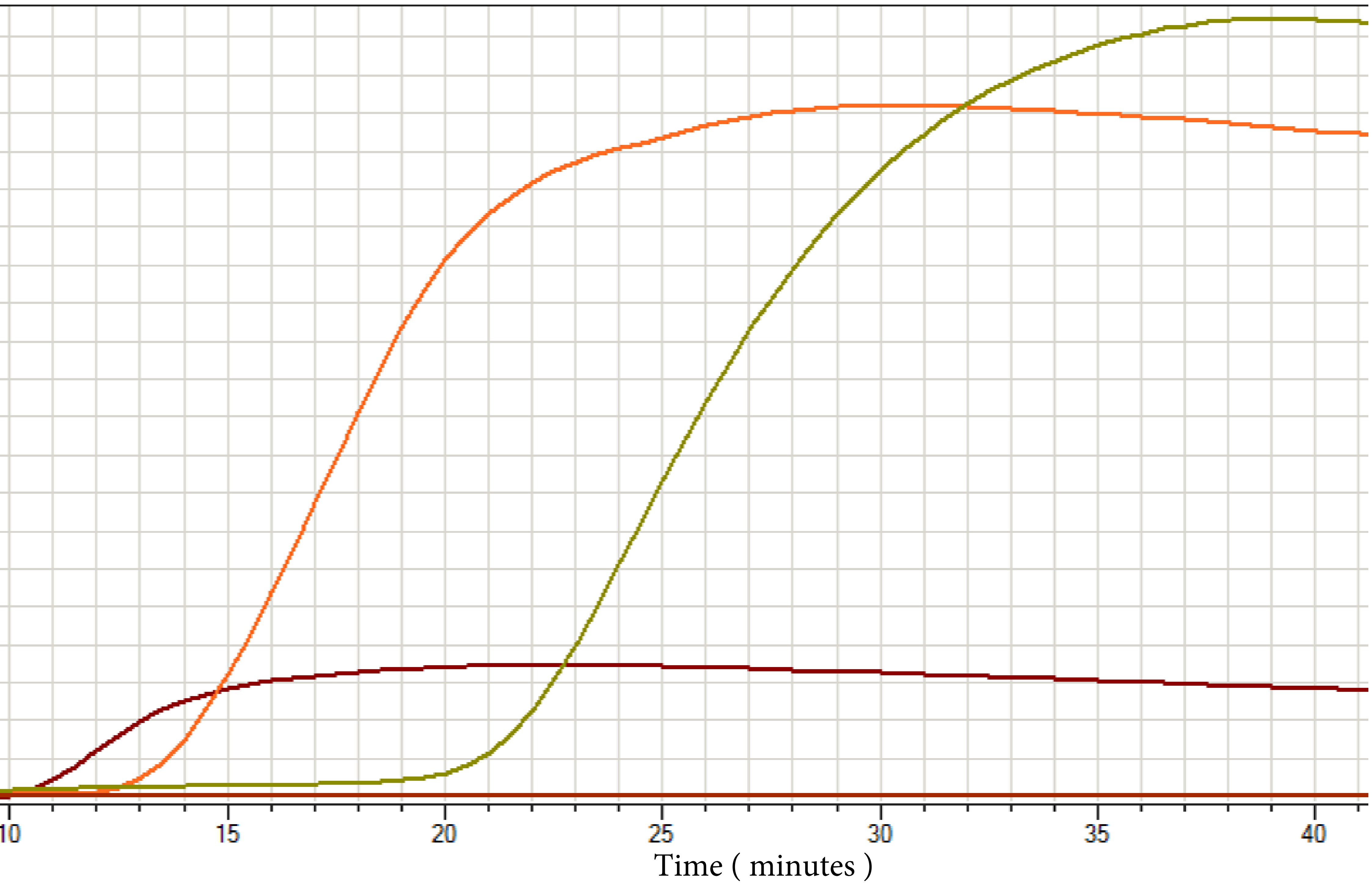

Algorithm Processing Curve

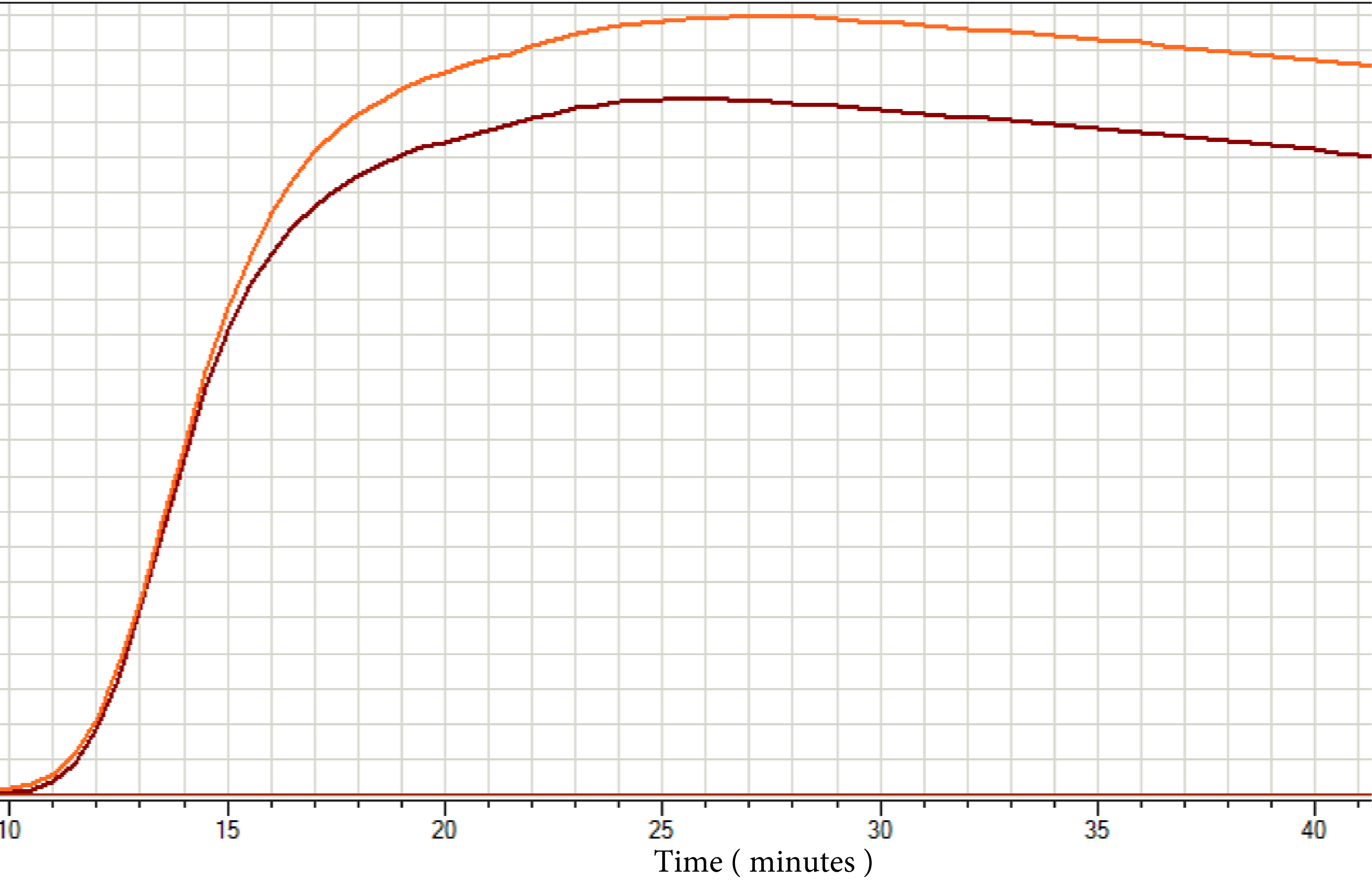

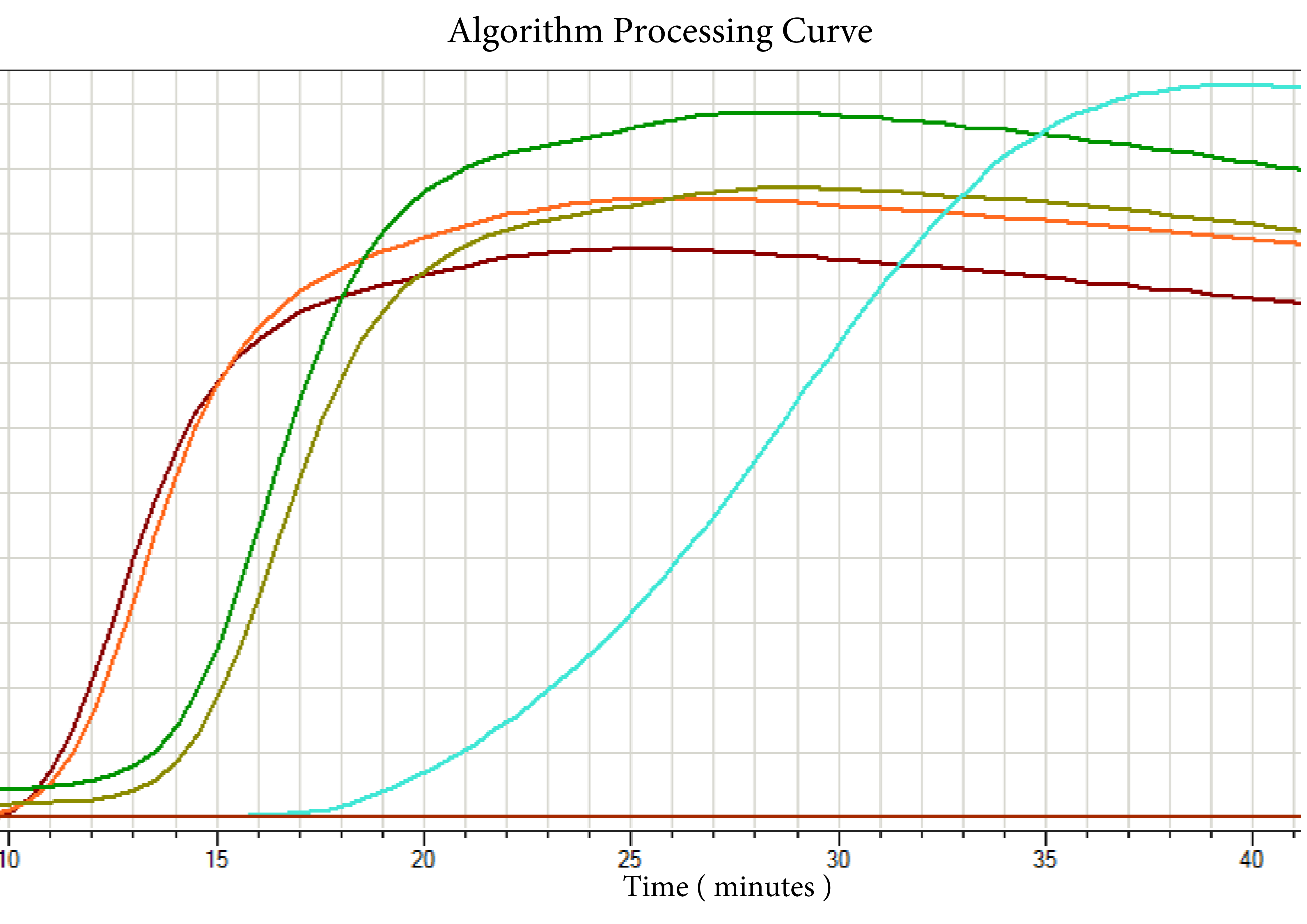

Algorithm Processing Curve
10
15
20
25
30
35
40
Time (minutes)

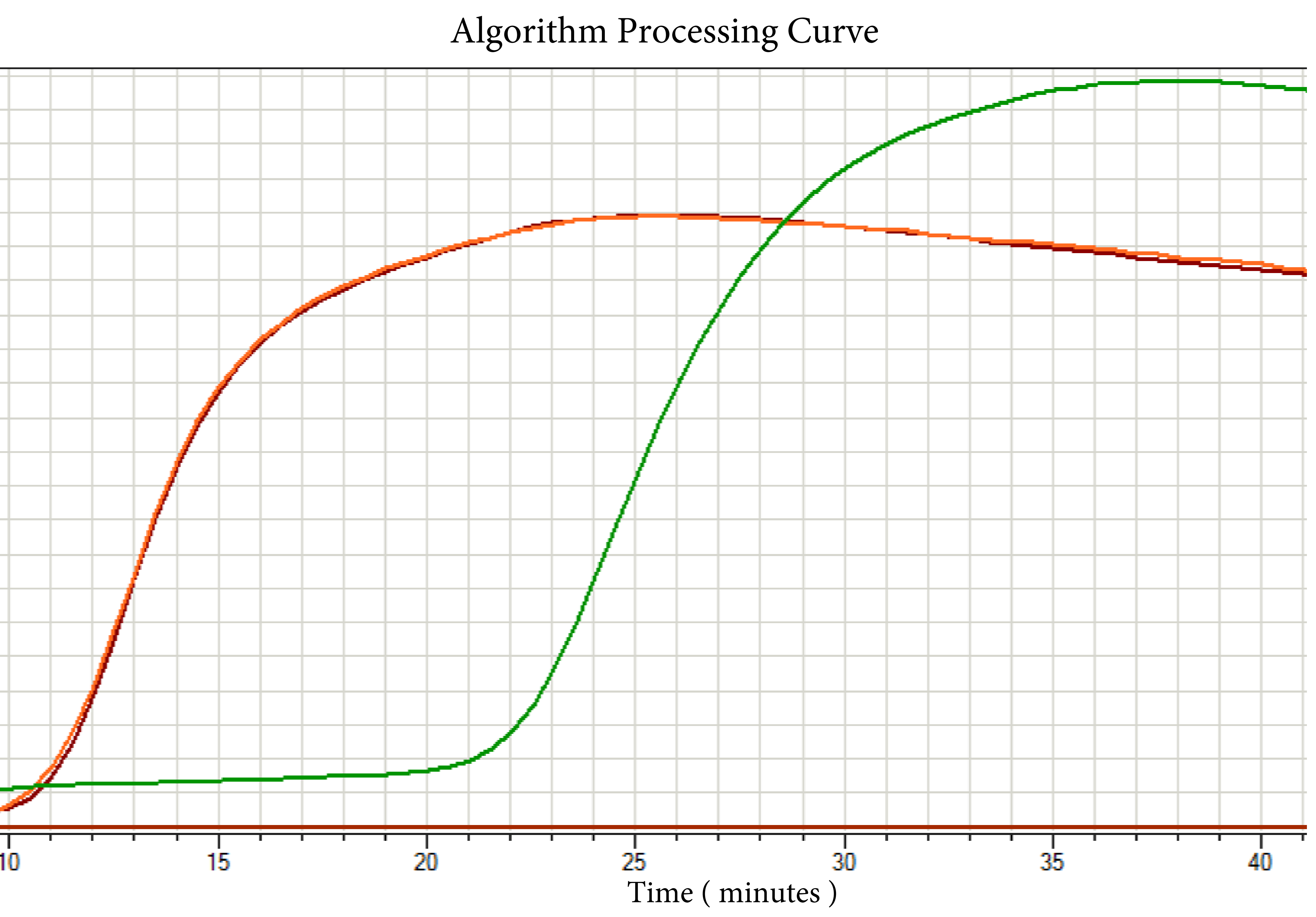
Algorithm Processing Curve
10
15
20
25
30
35
40
Time (minutes)

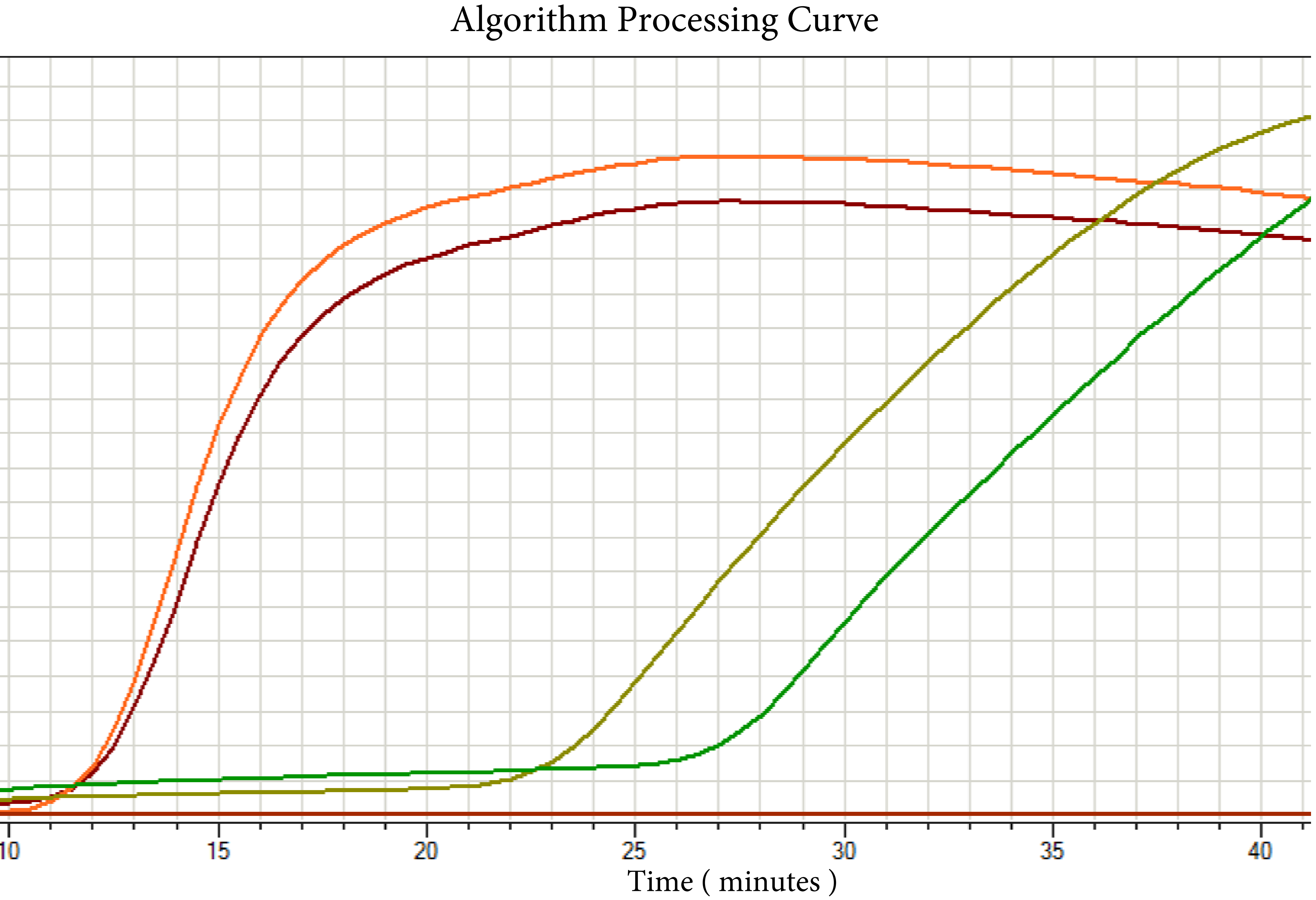

Algorithm Processing Curve
10
15
20
25
30
35
40
Time (minutes)

Algorithm Processing Curve

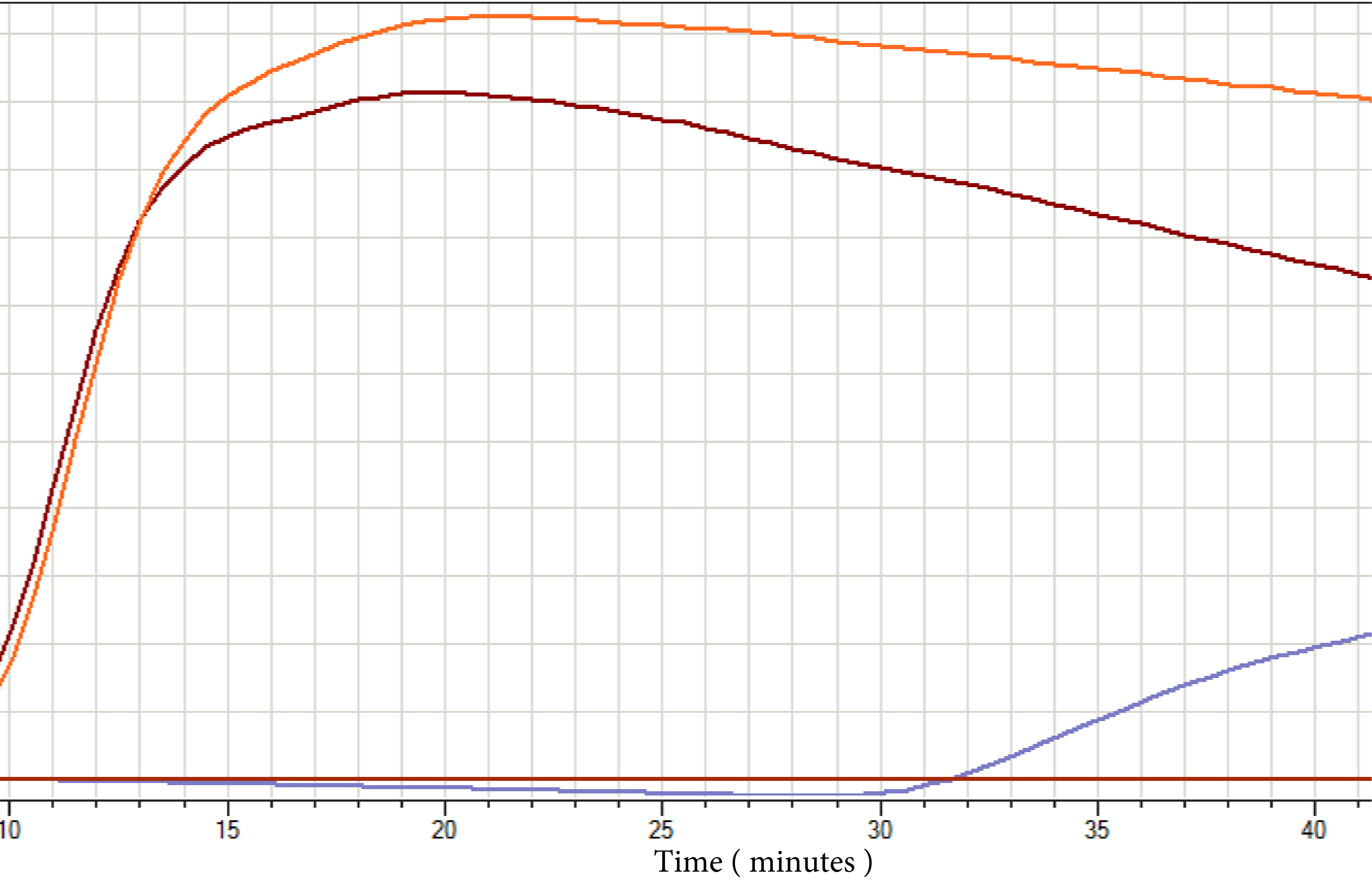

Algorithm Processing Curve

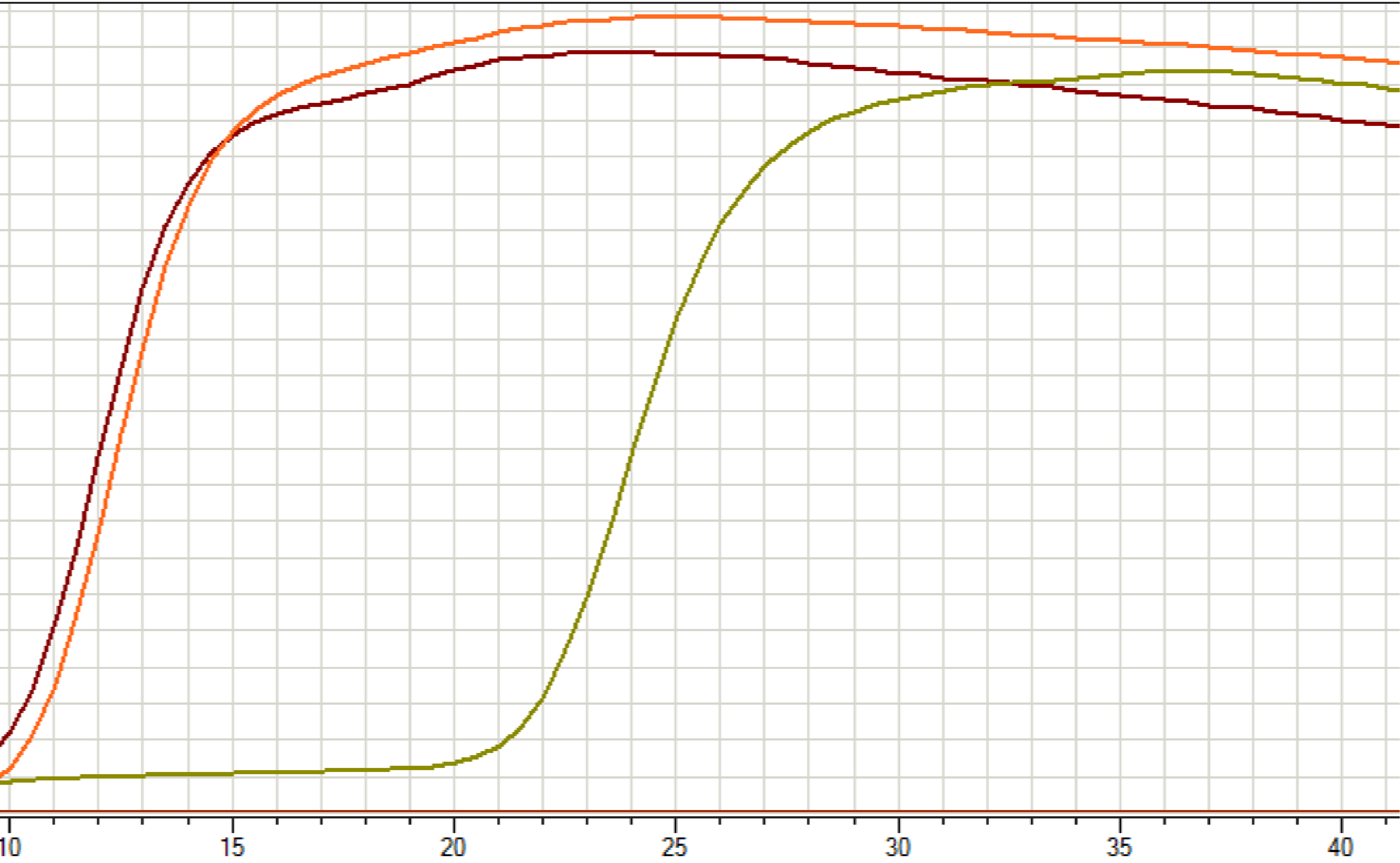

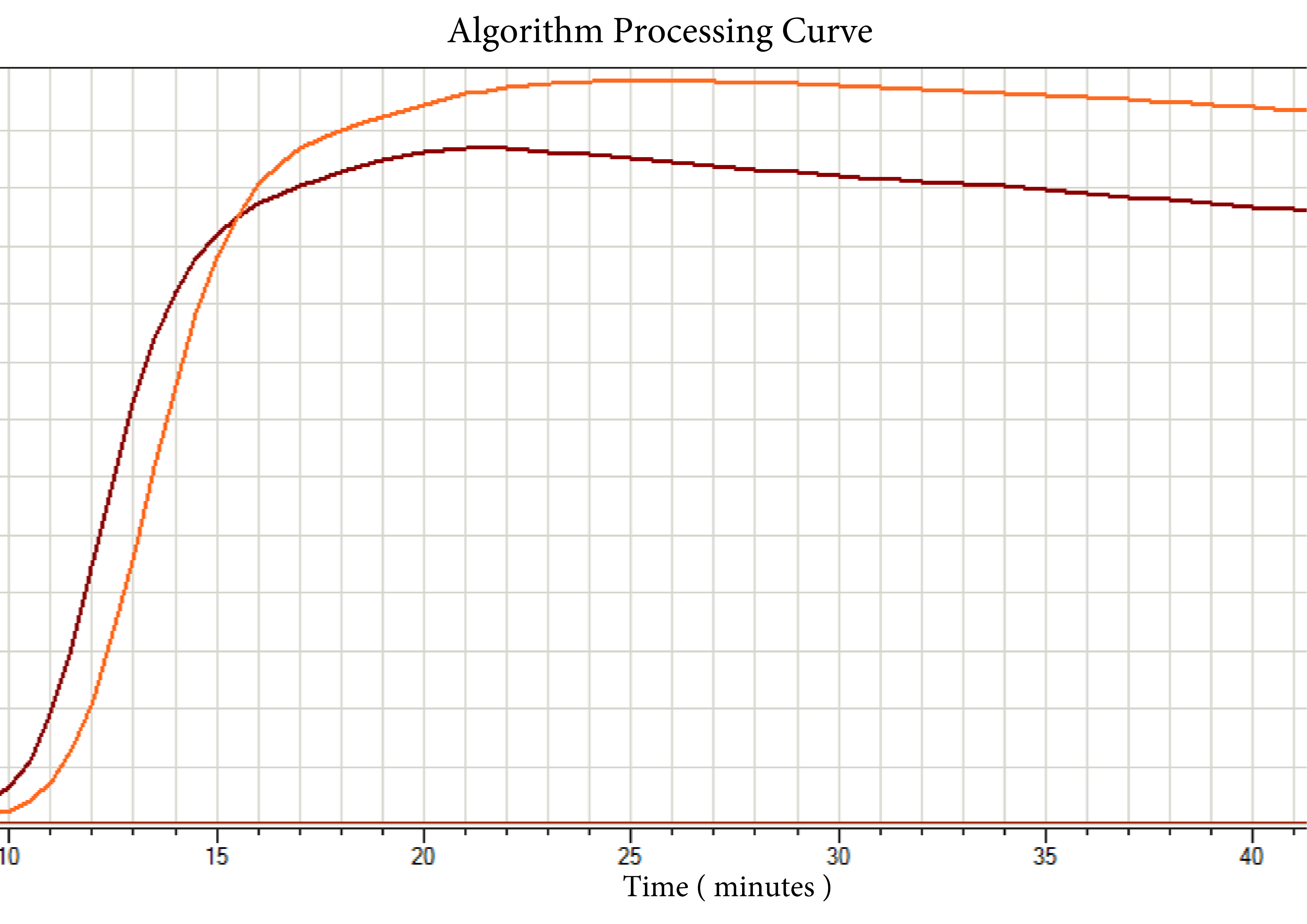

Algorithm Processing Curve
10
15
20
25
30
35
40
Time (minutes)

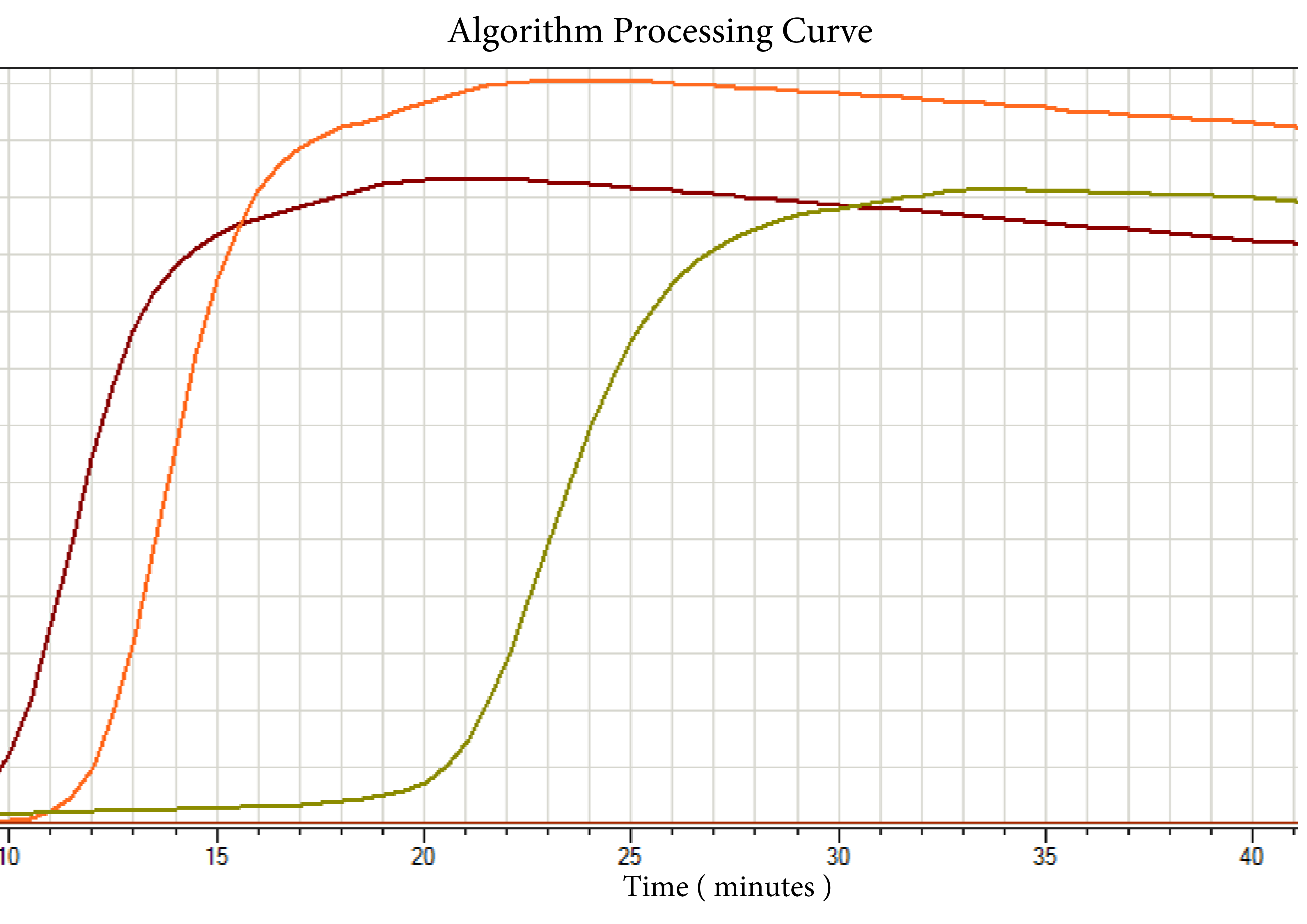
Algorithm Processing Curve
10
15
20
25
30
35
40
Time (minutes)

Algorithm Processing Curve

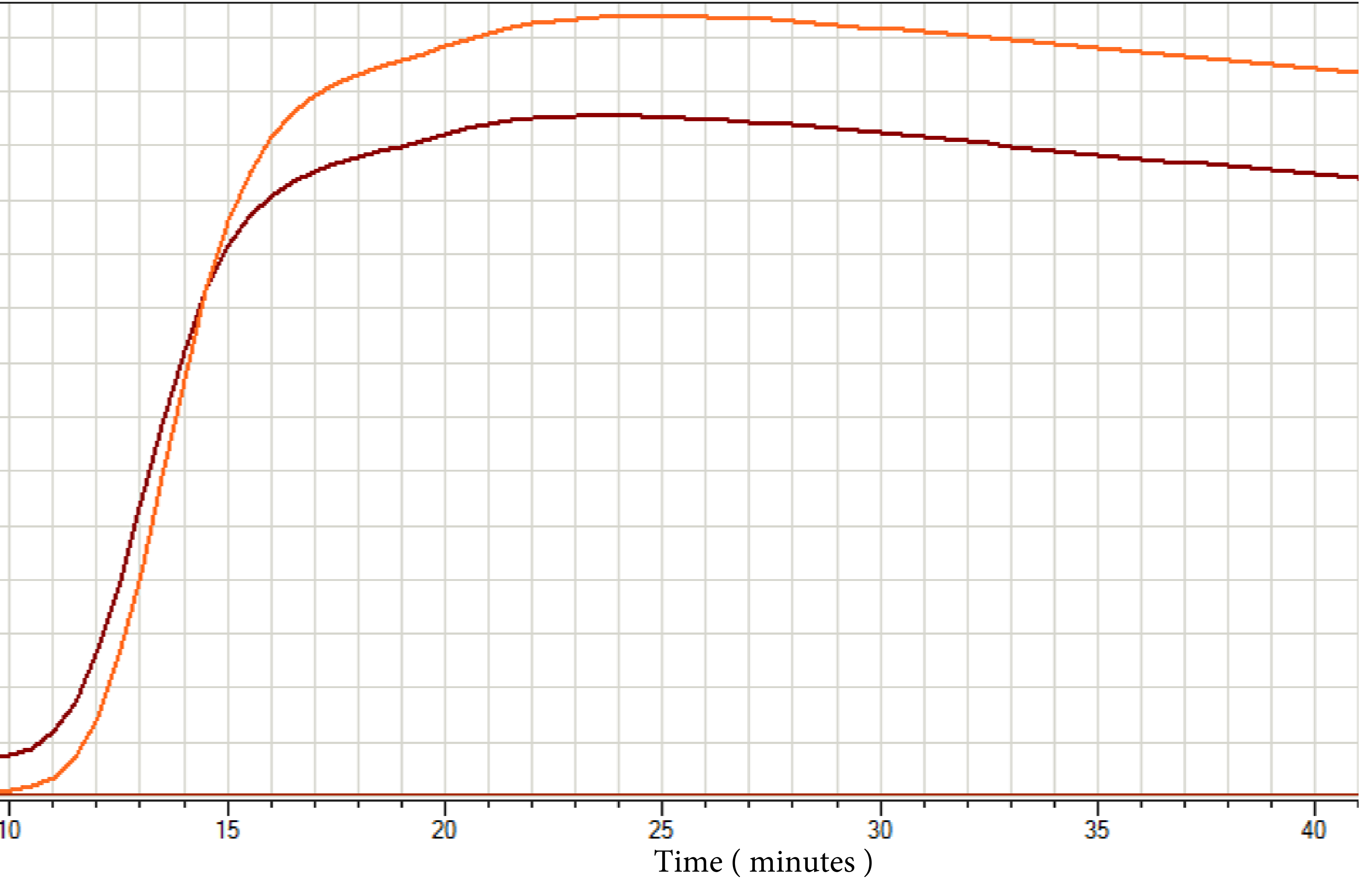

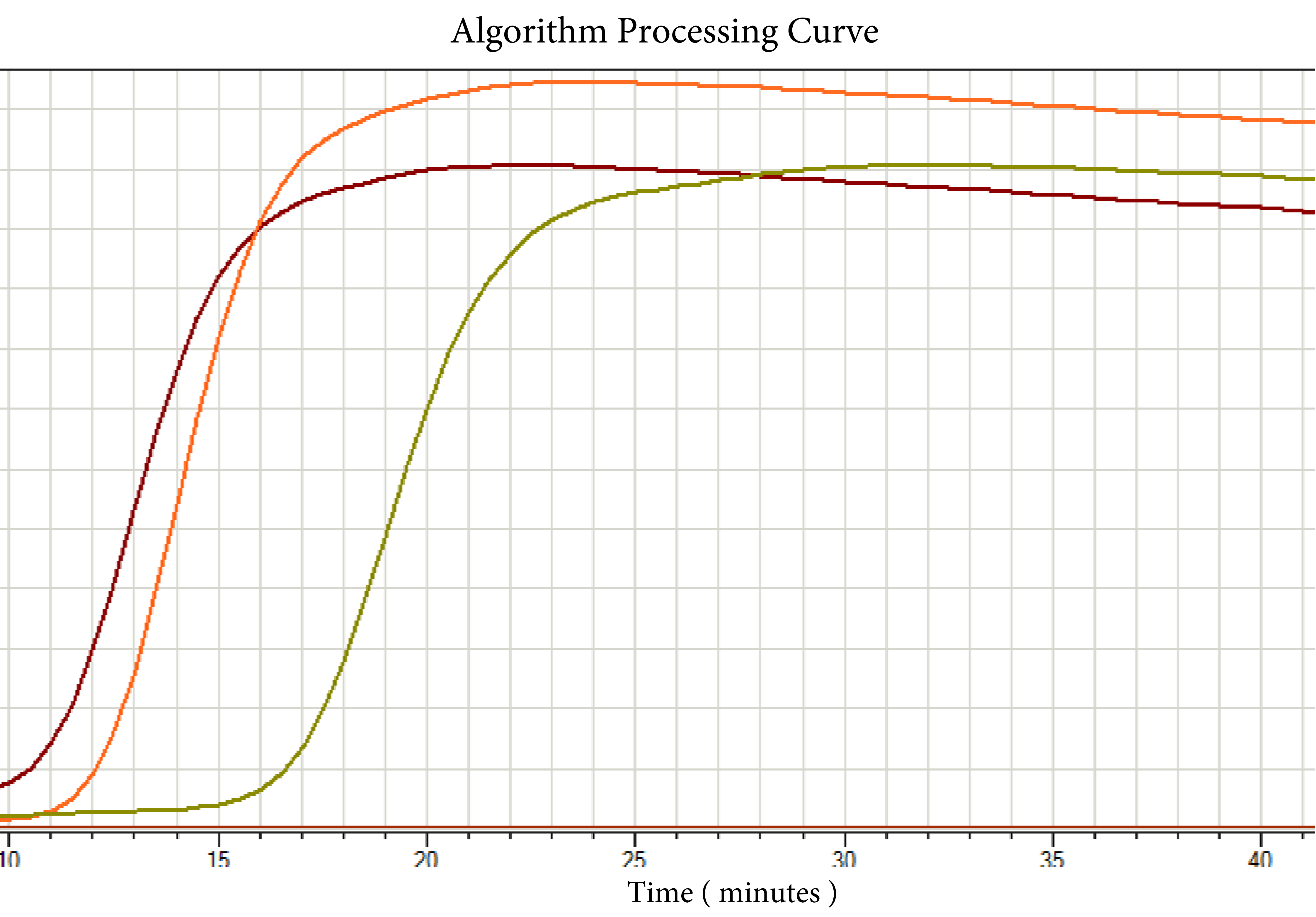
Algorithm Processing Curve
10
15
20
25
30
35
40
Time (minutes)

Real-time Fluorescence Curve

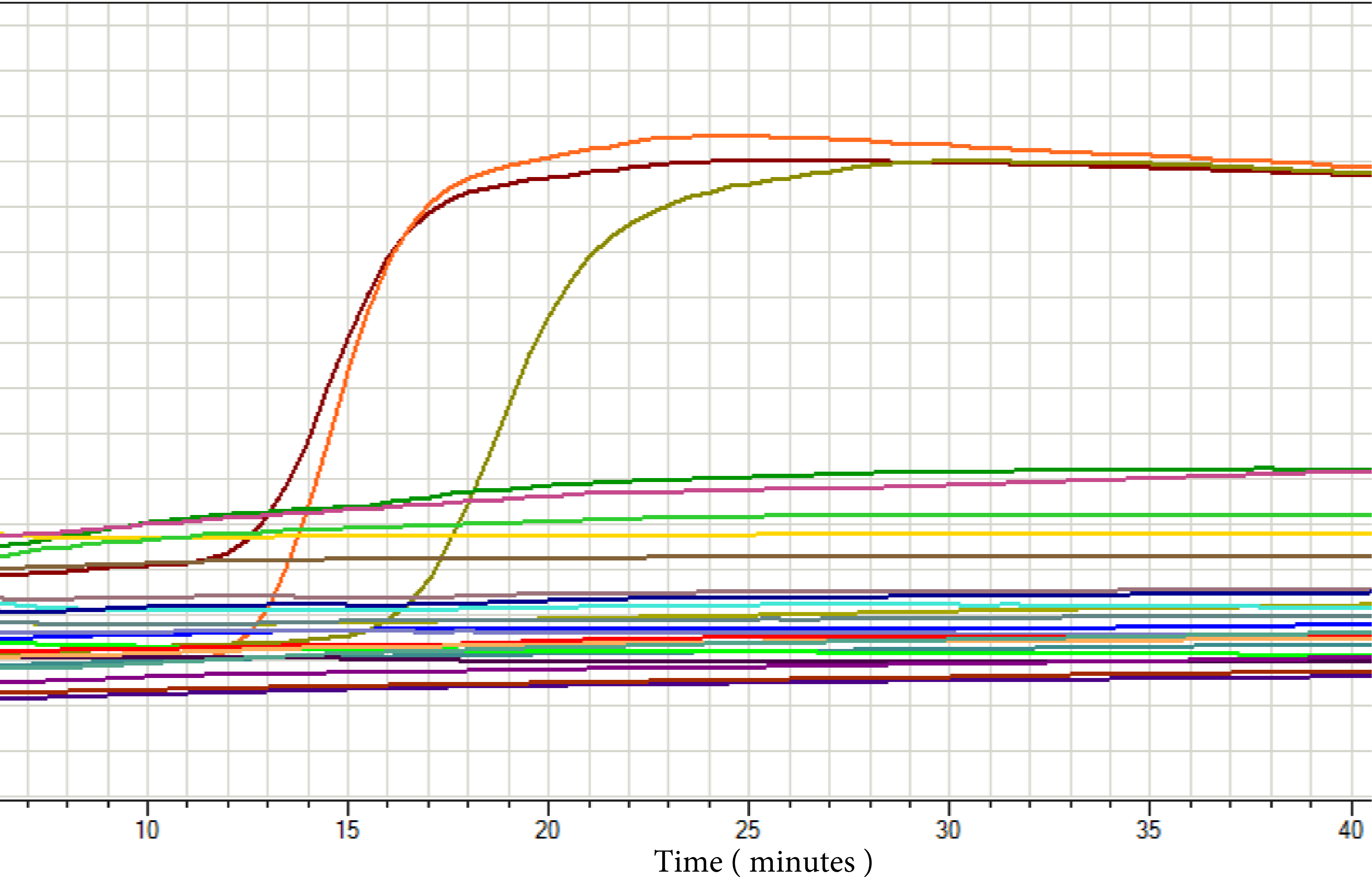

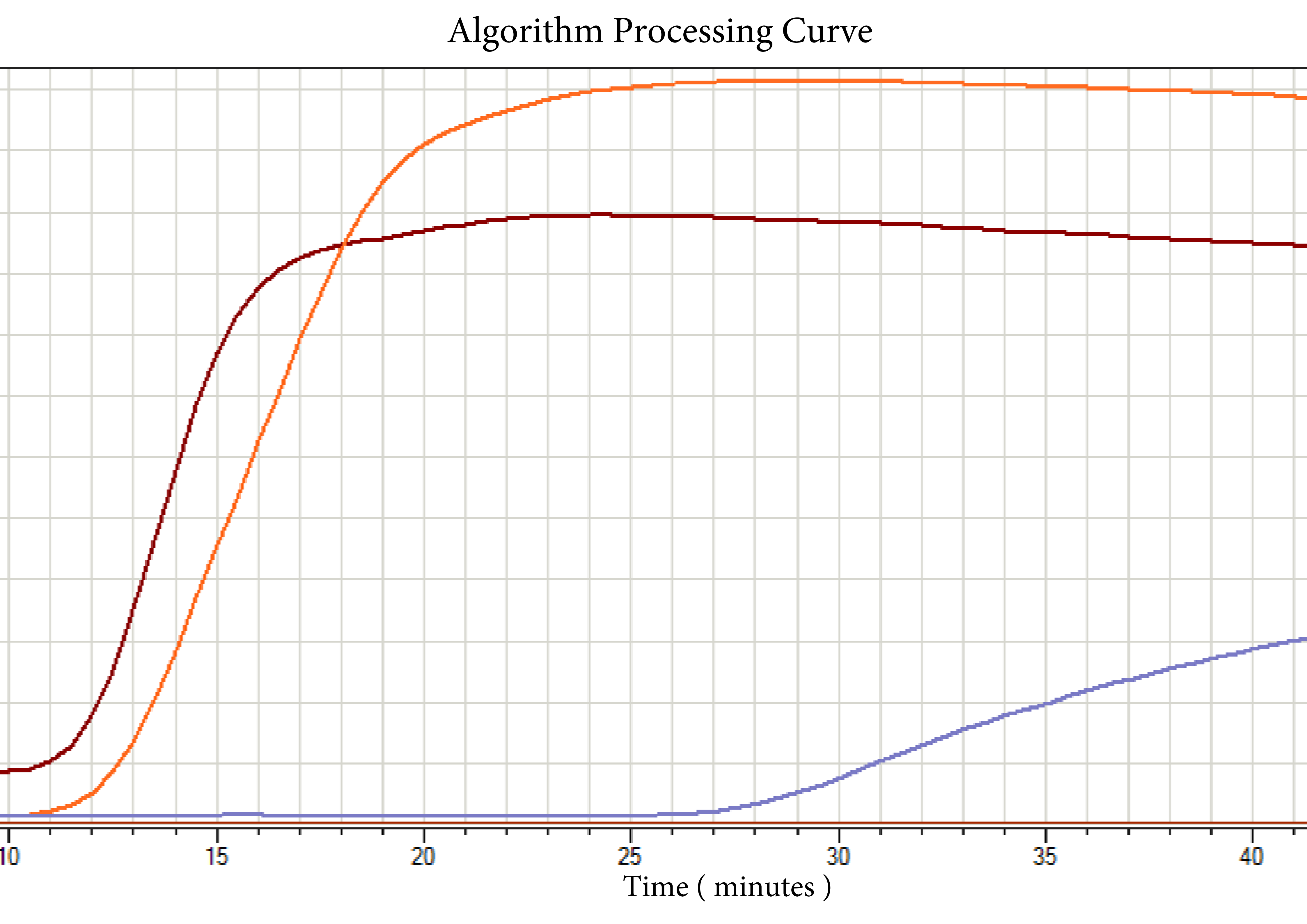
Algorithm Processing Curve
10
15
20
25
30
35
40
Time (minutes)

Algorithm Processing Curve

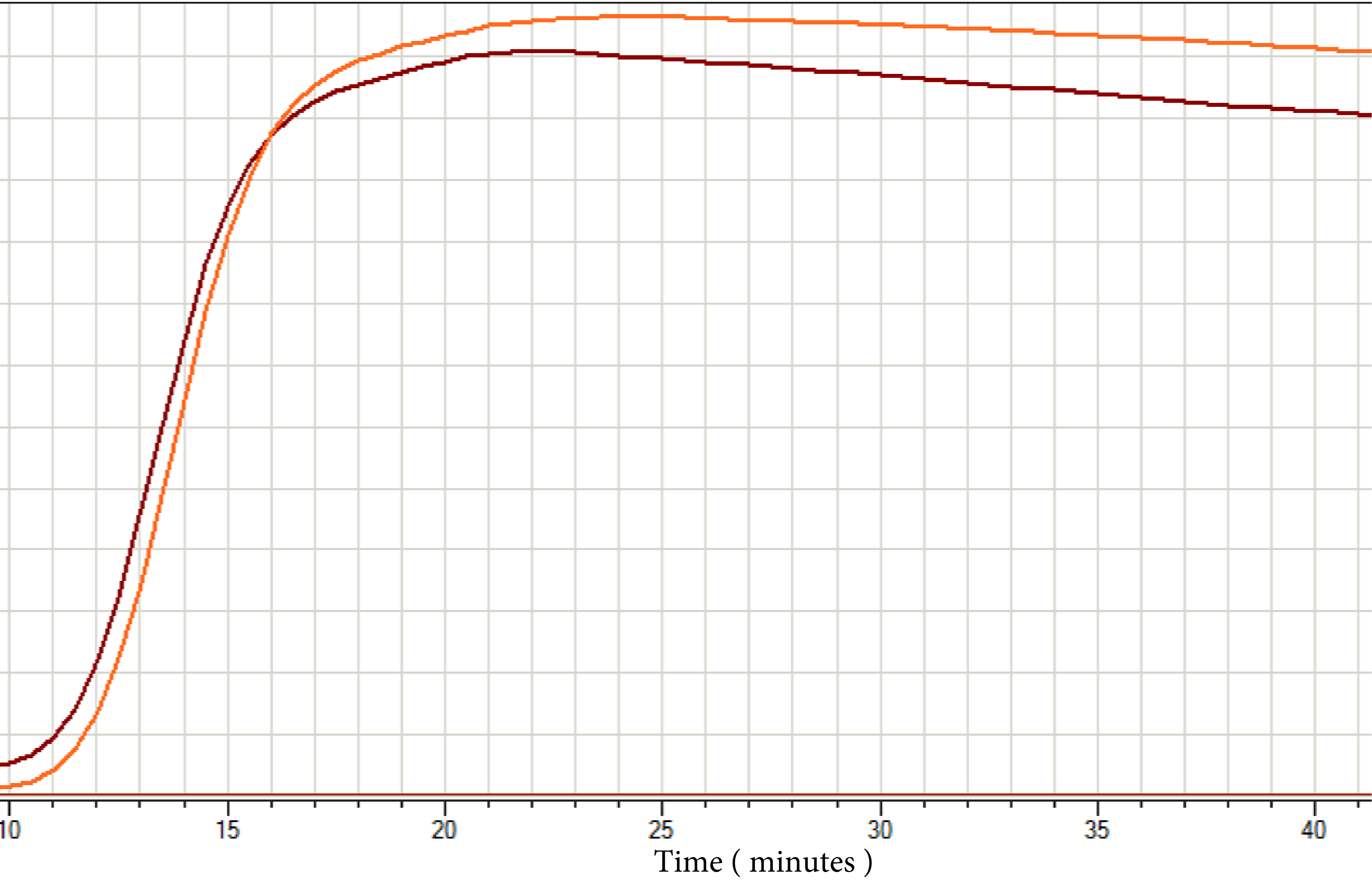

Algorithm Processing Curve

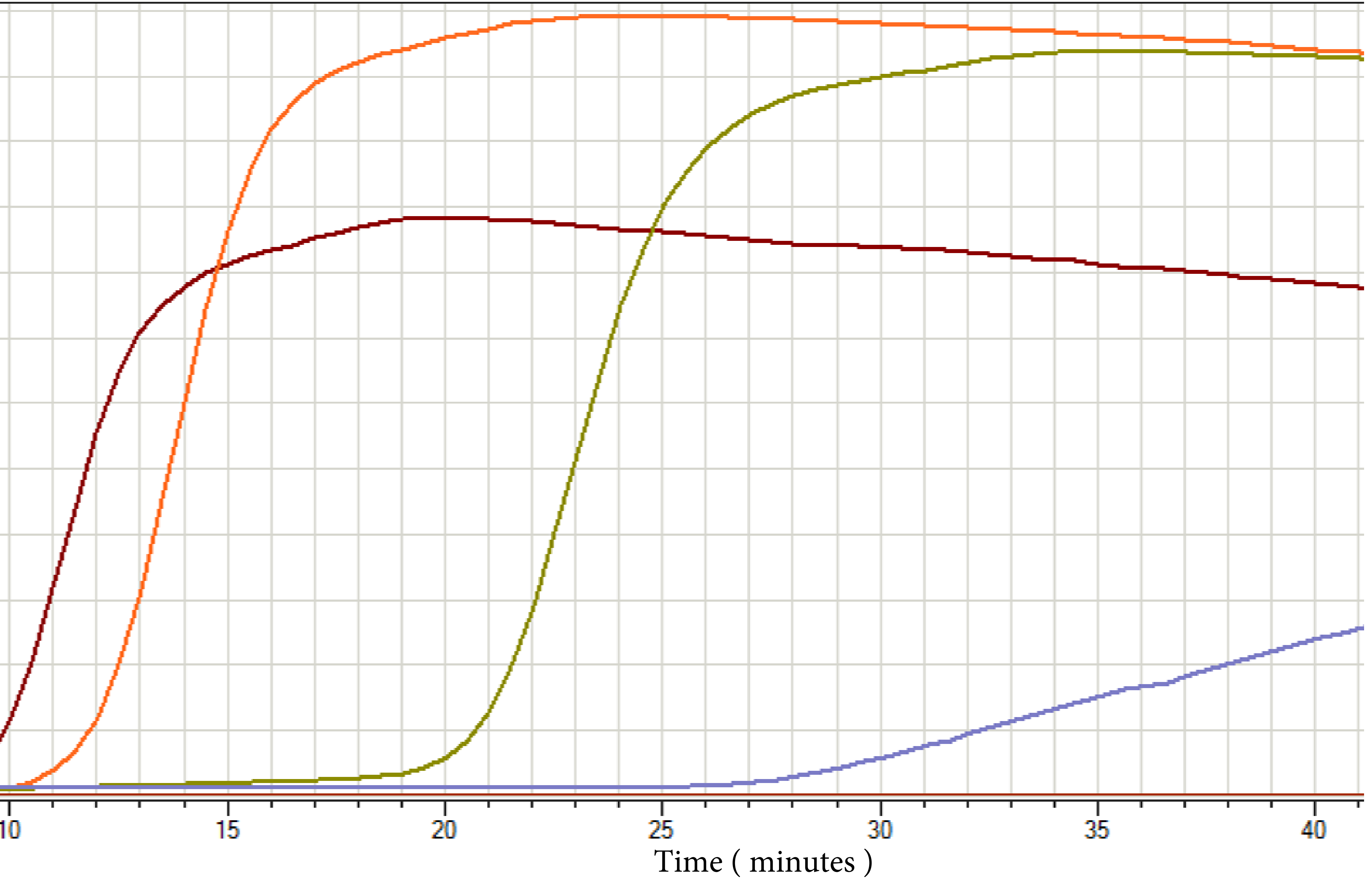

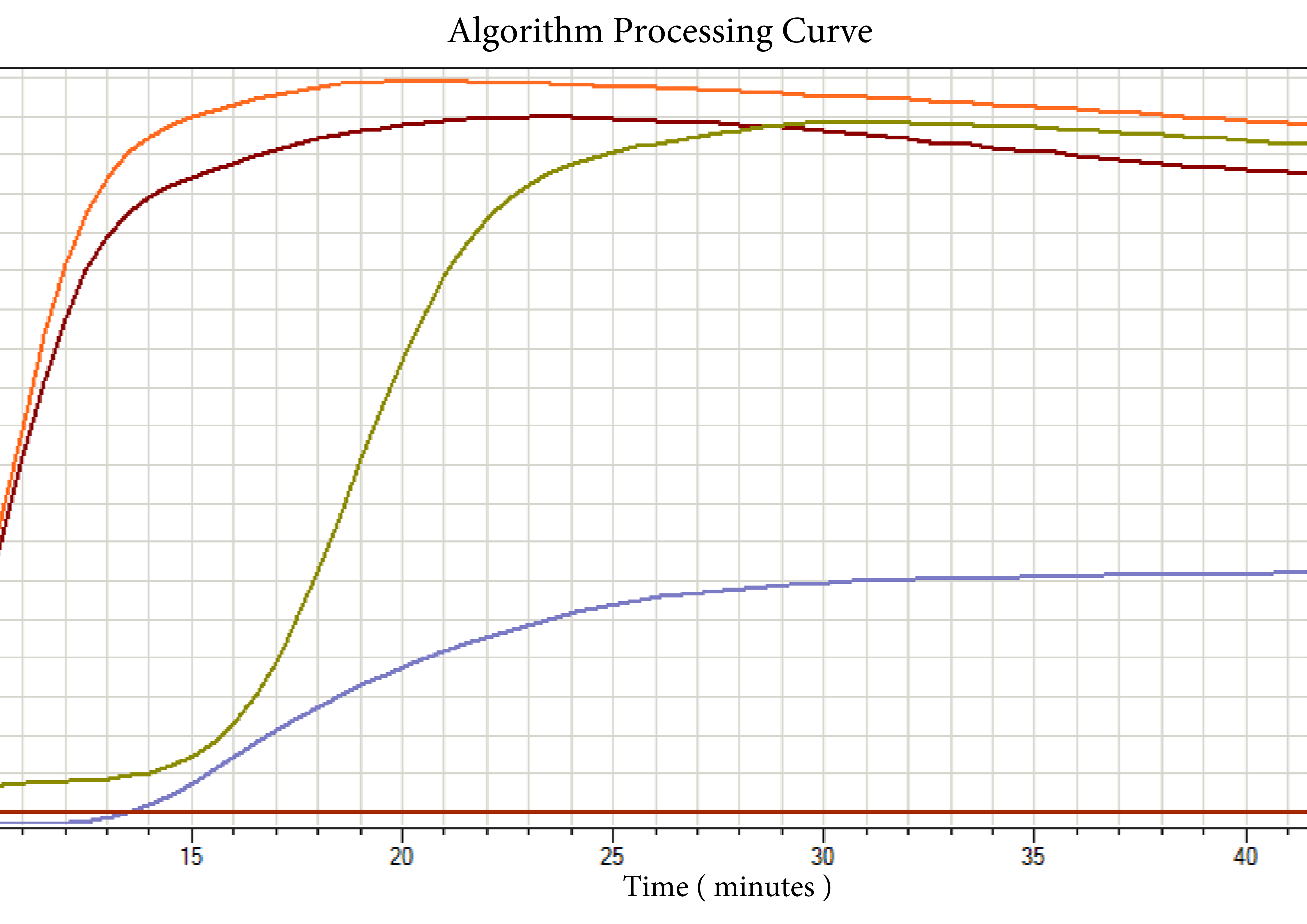
Algorithm Processing Curve
15
20
25
30
35
40
Time (minutes)

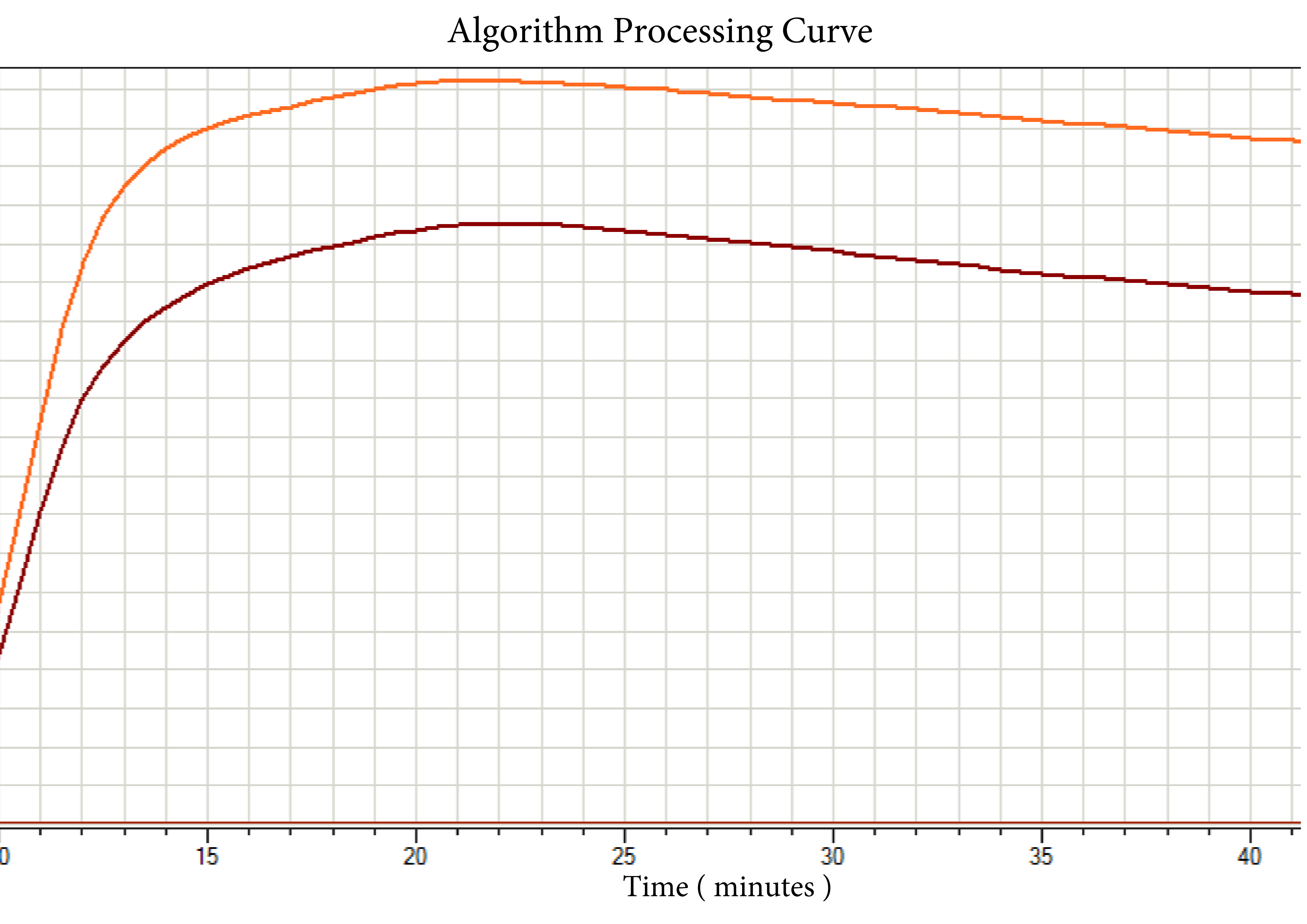
Algorithm Processing Curve
0
15
20
25
30
35
40
Time (minutes)

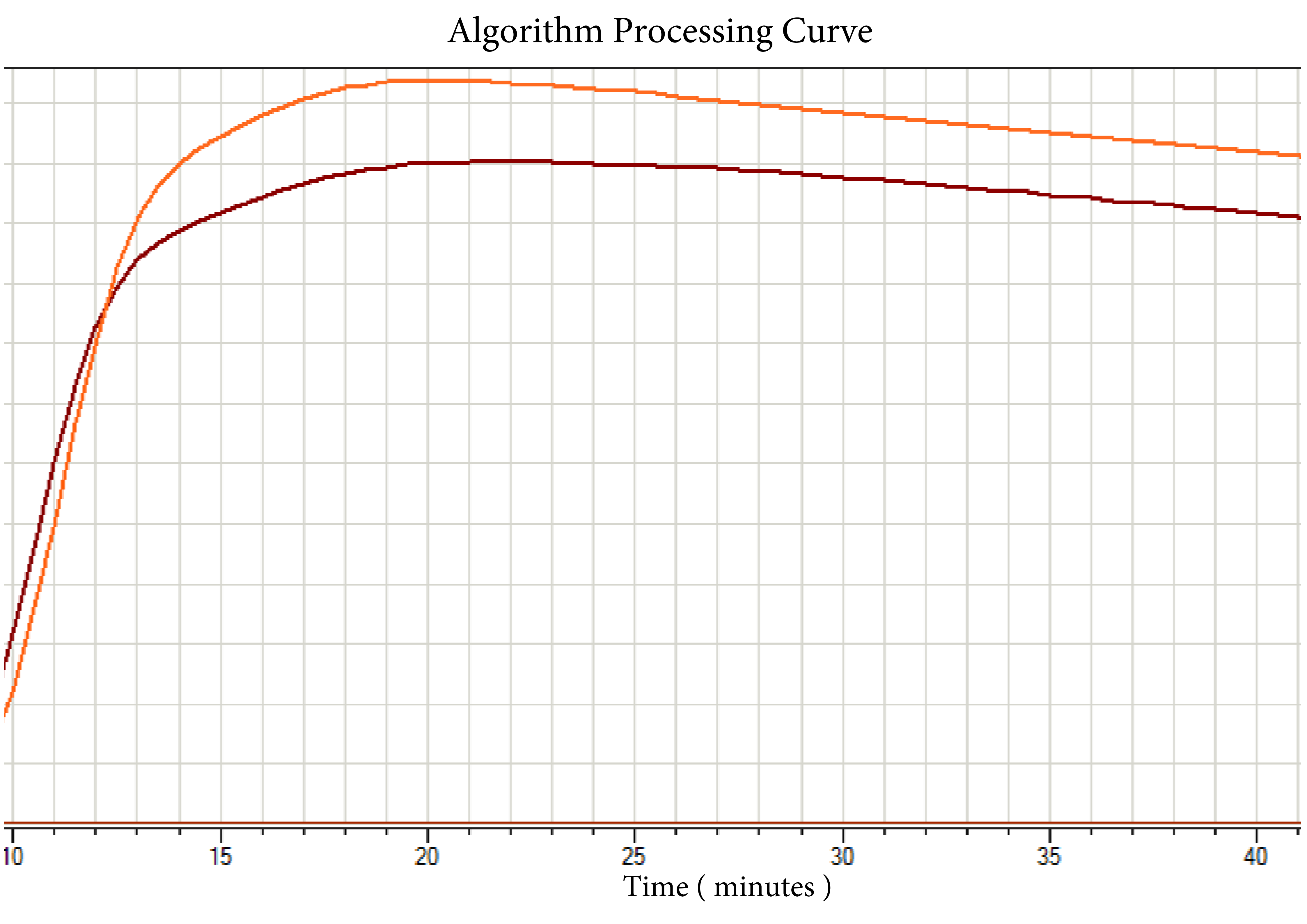

Algorithm Processing Curve
10
15
20
25
30
35
40
Time (minutes)

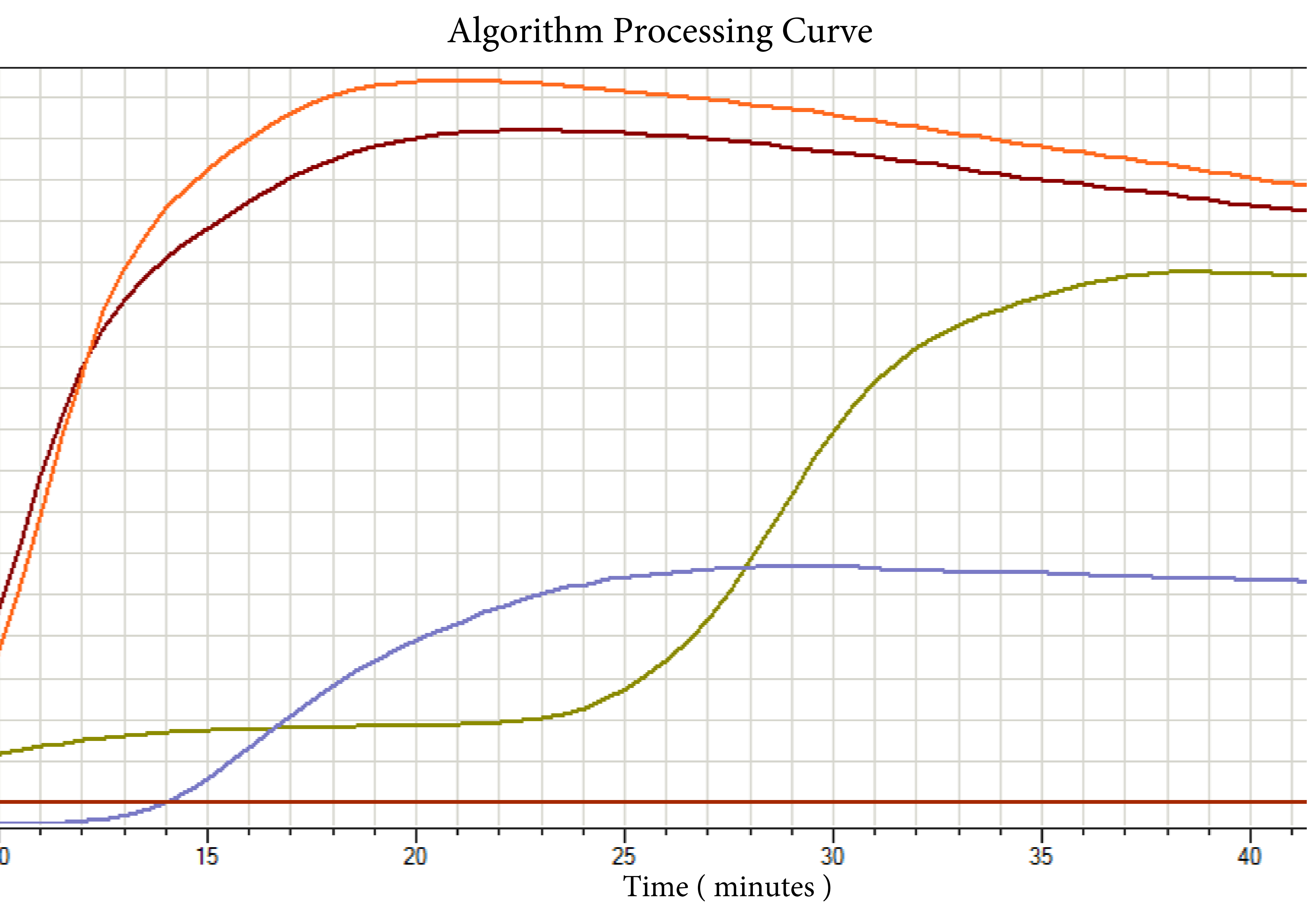

Algorithm Processing Curve
0
15
20
25
30
35
40
Time (minutes)

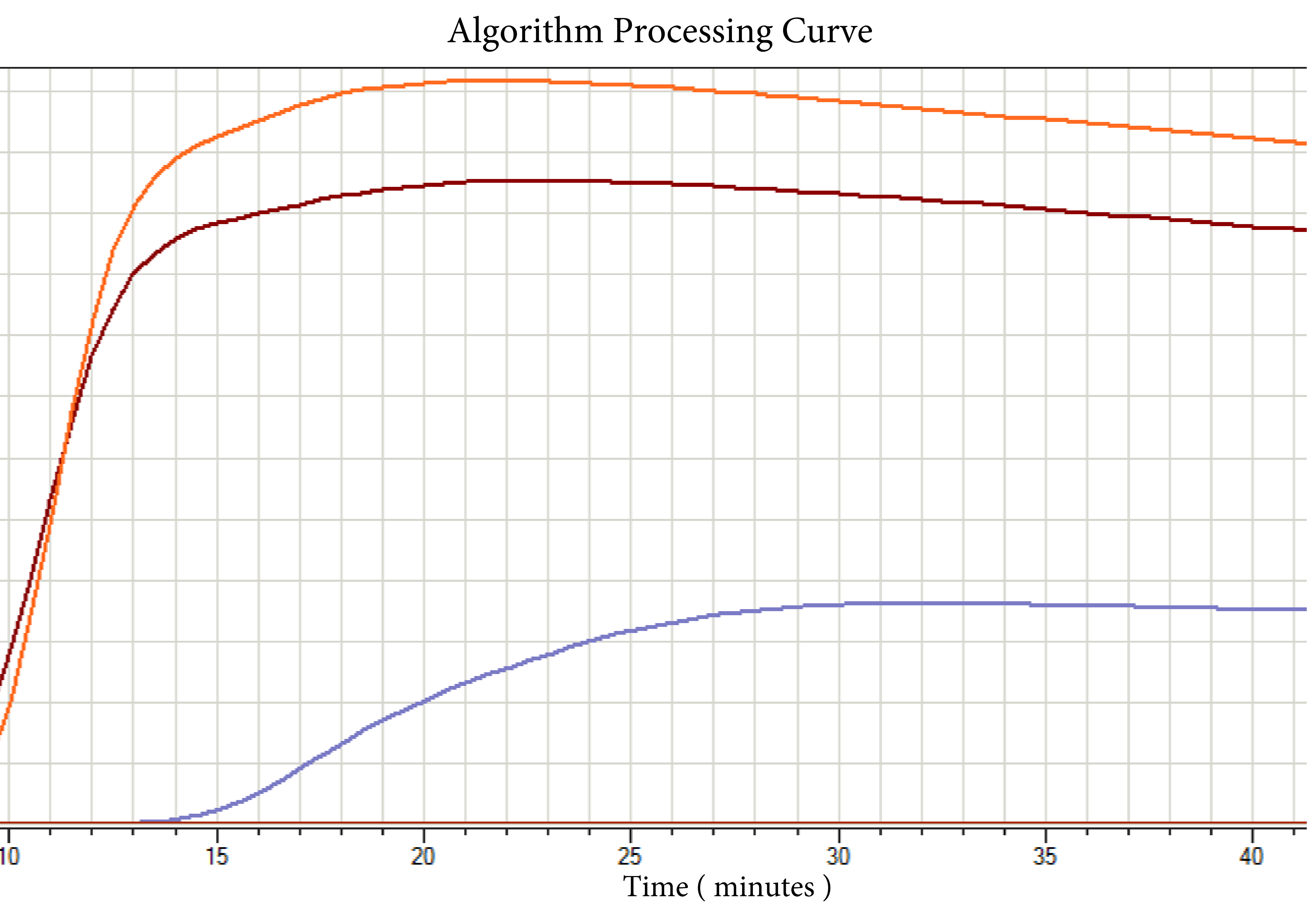
Algorithm Processing Curve
10
15
20
25
30
35
40
Time (minutes)

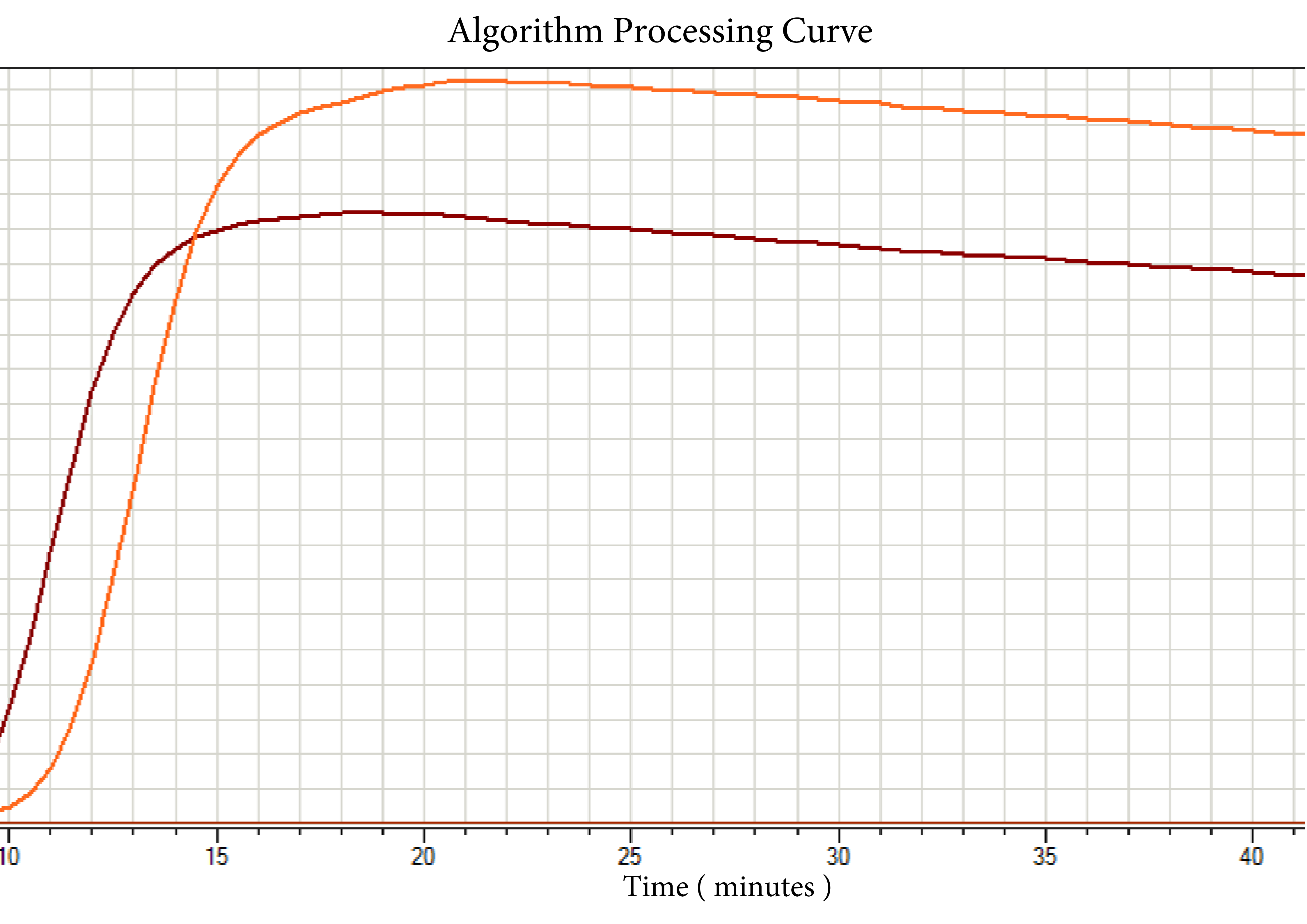

Algorithm Processing Curve
10
15
20
25
30
35
40
Time (minutes)

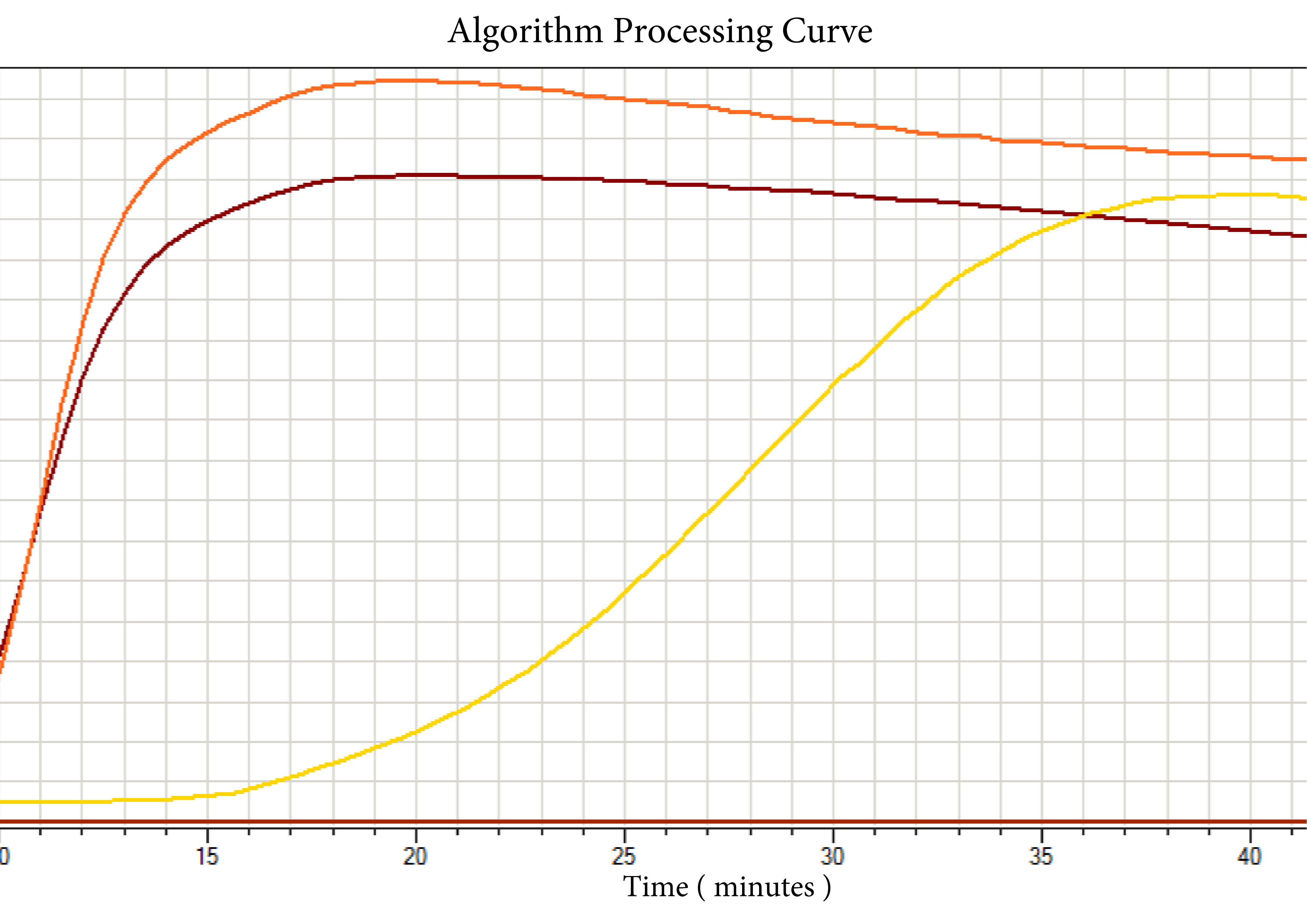
Algorithm Processing Curve
0
15
20
25
30
35
40
Time (minutes)

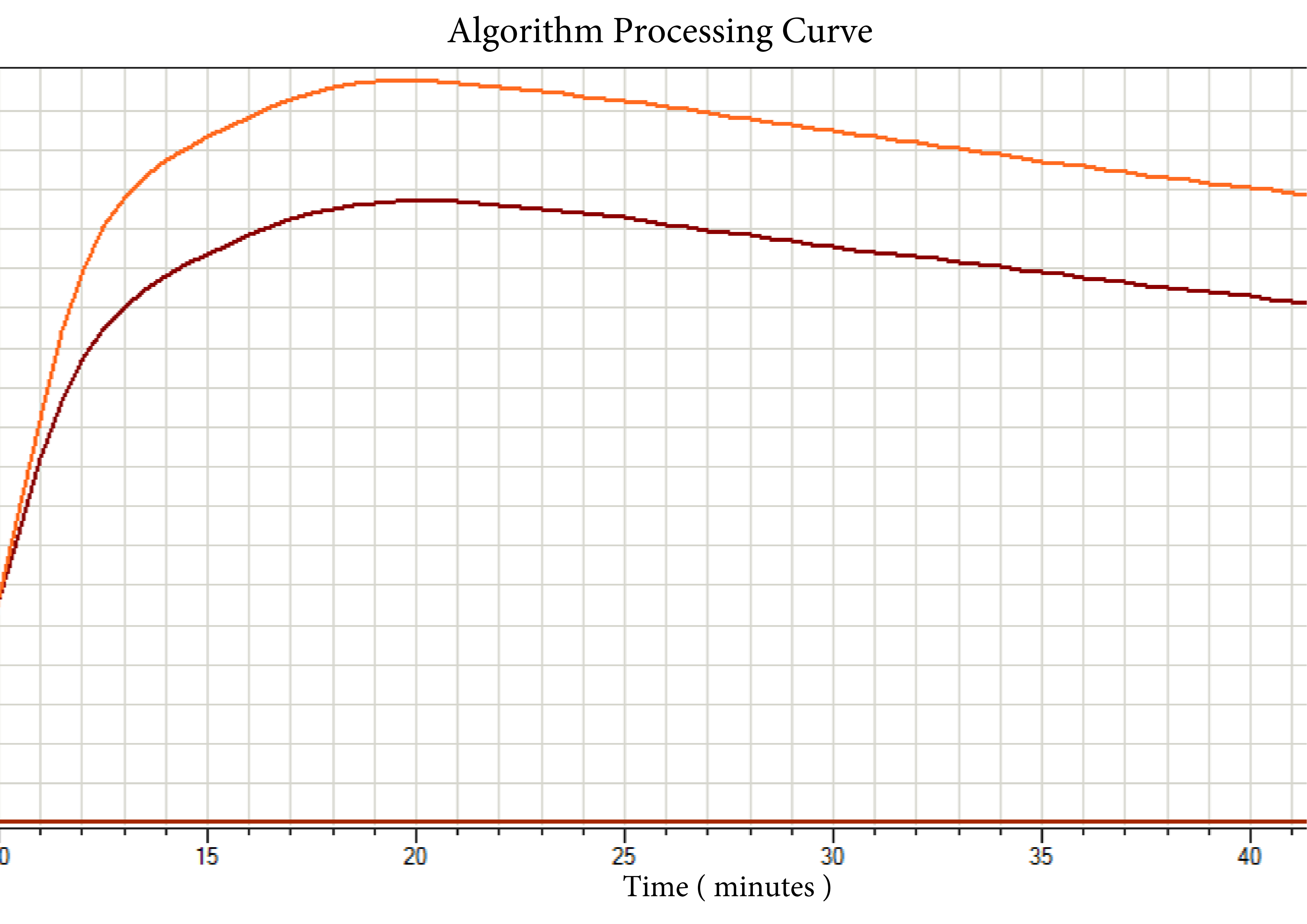
Algorithm Processing Curve
0
15
20
25
30
35
40
Time (minutes)

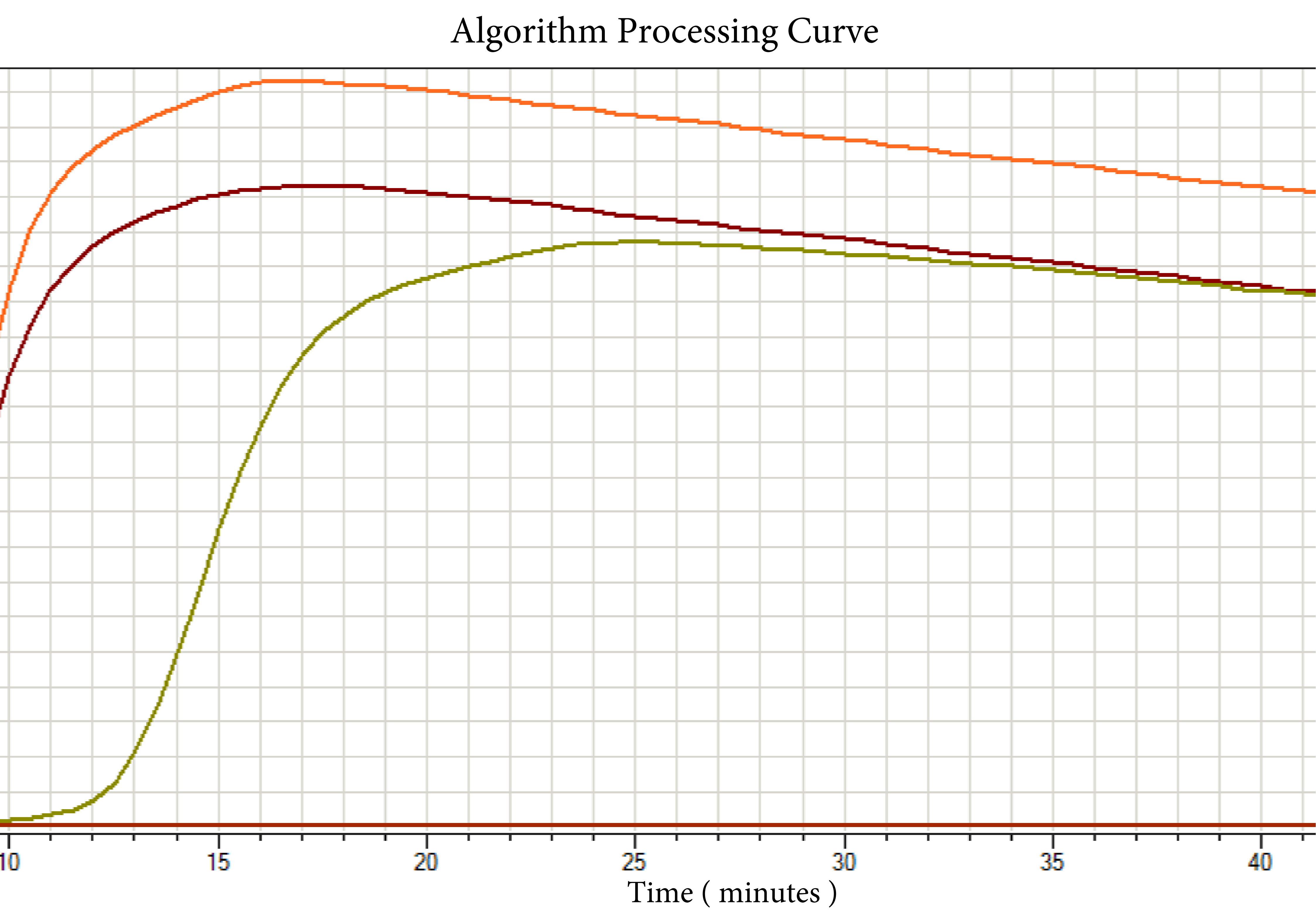

Algorithm Processing Curve
10
15
20
25
30
35
40
Time (minutes)

Algorithm Processing Curve

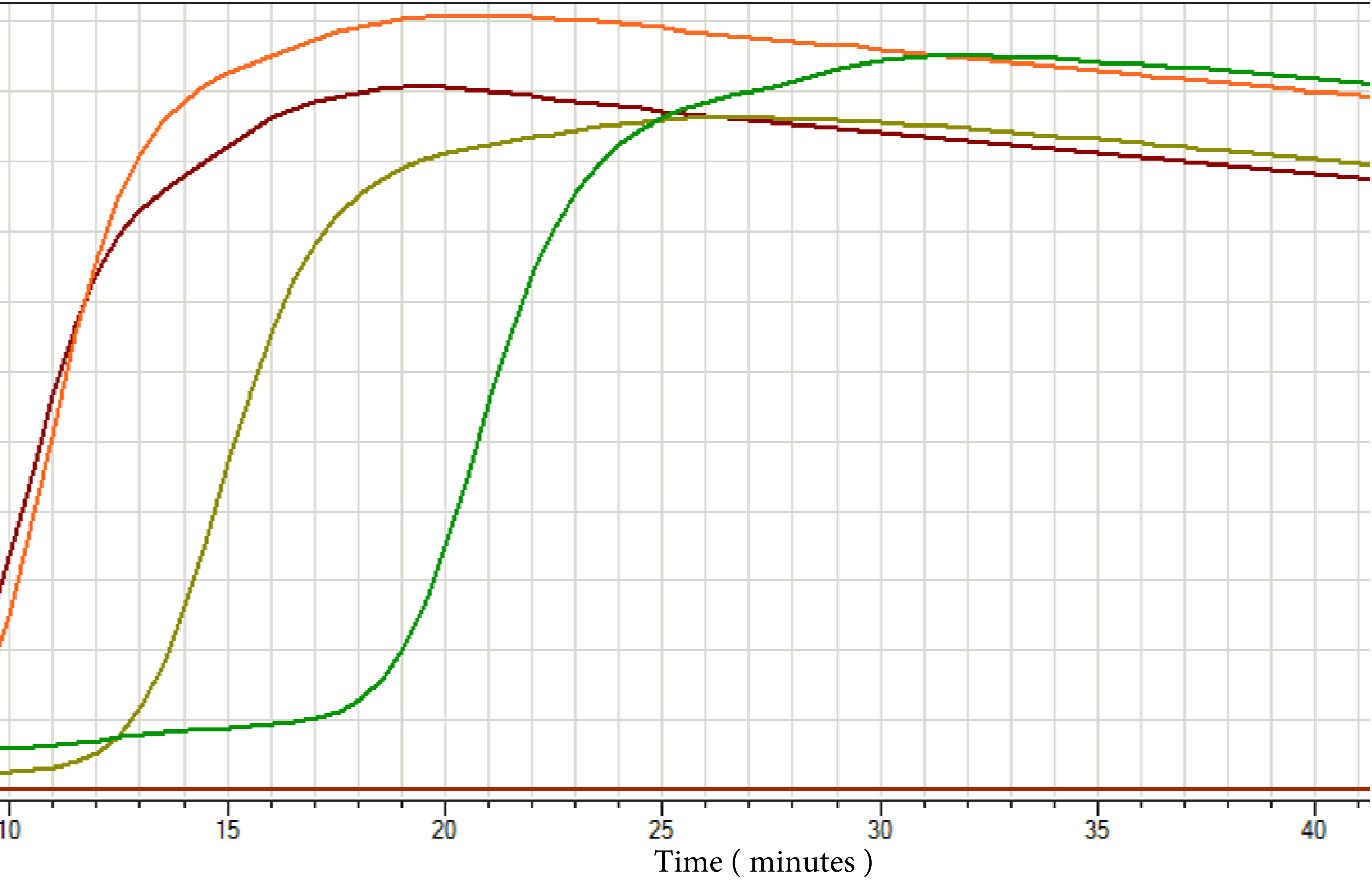

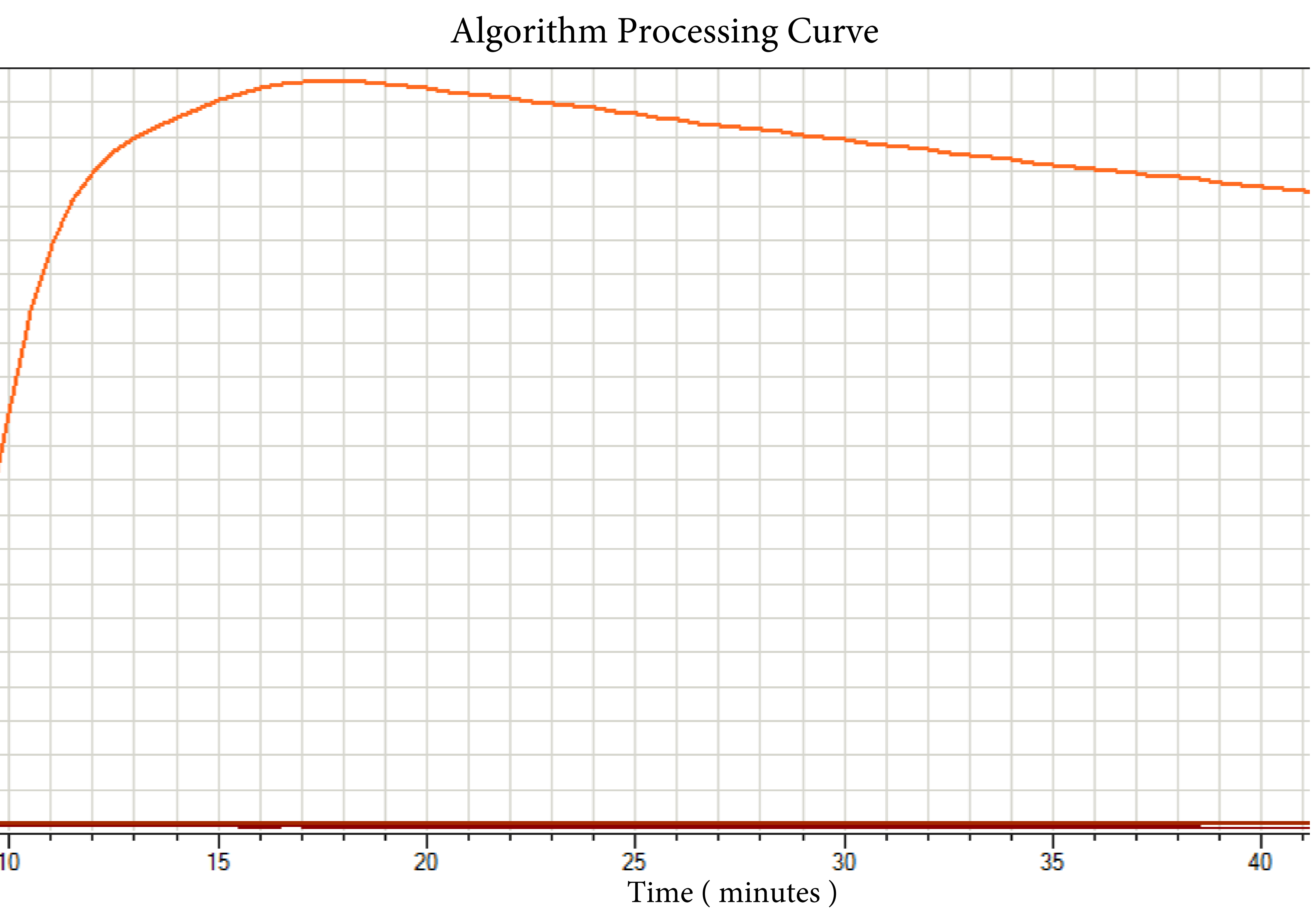
Algorithm Processing Curve
10
15
20
25
30
35
40
Time (minutes)

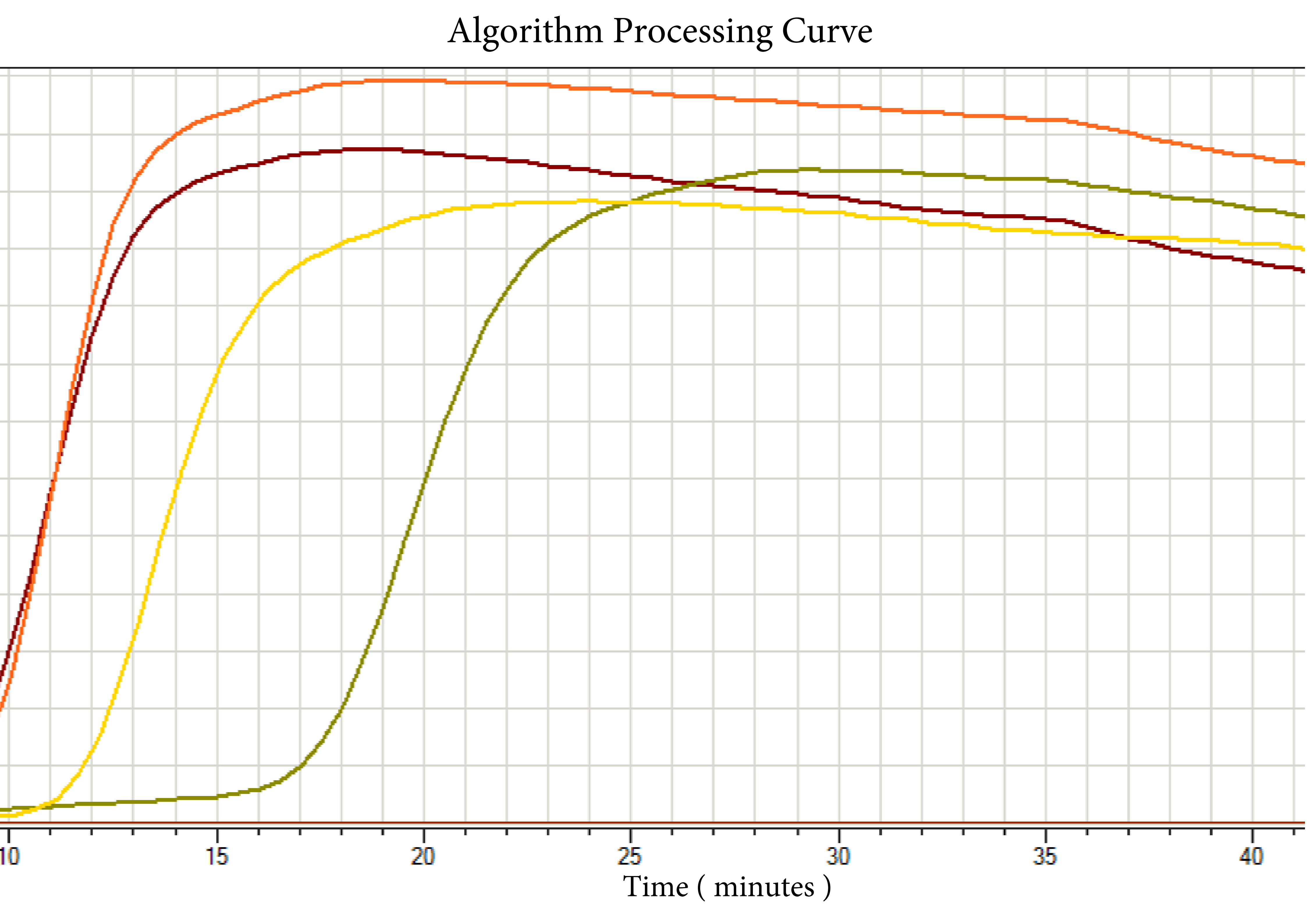

Algorithm Processing Curve
10
15
20
25
30
35
40
Time (minutes)

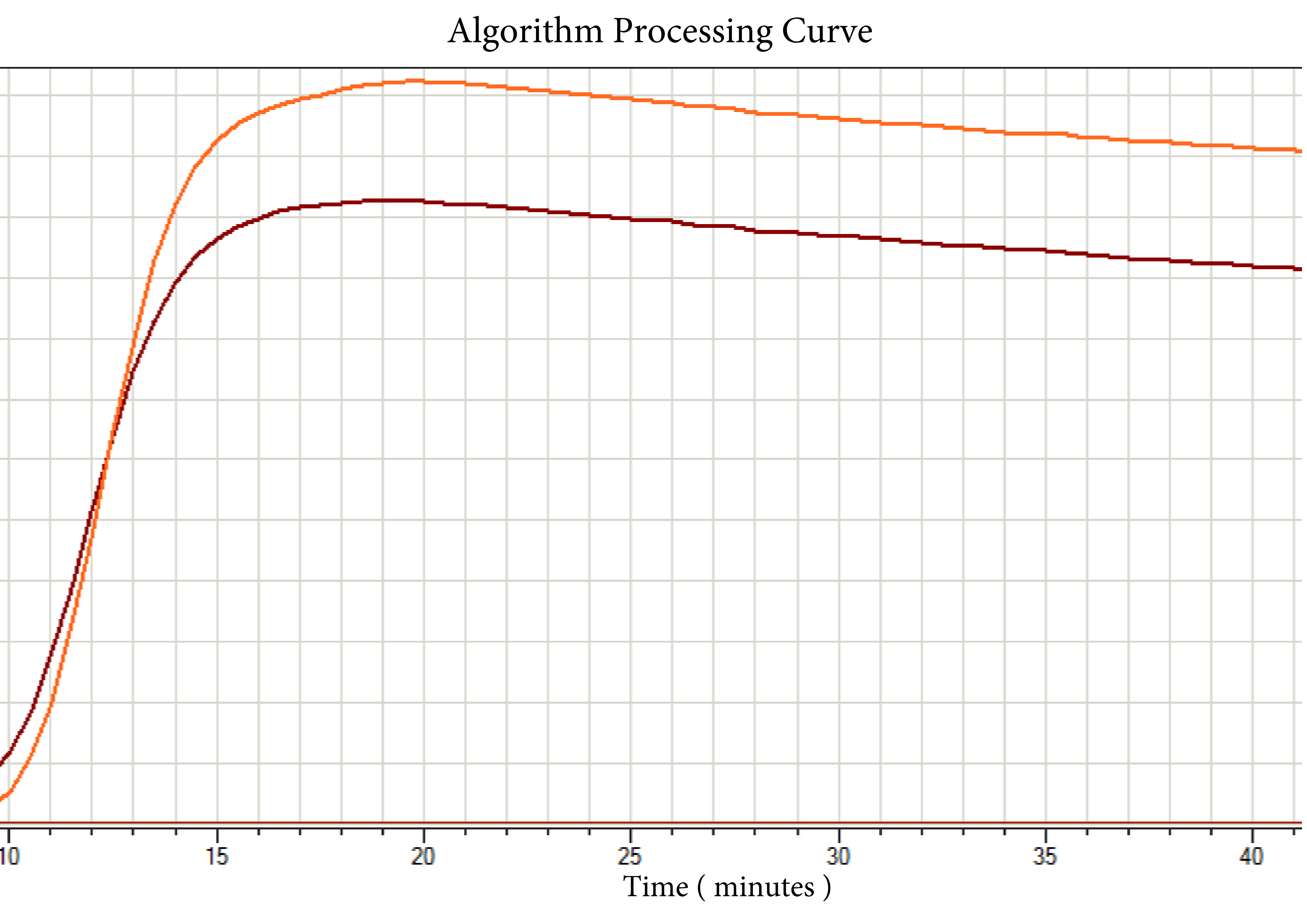

Algorithm Processing Curve
10
15
20
25
30
35
40
Time (minutes)

Algorithm Processing Curve

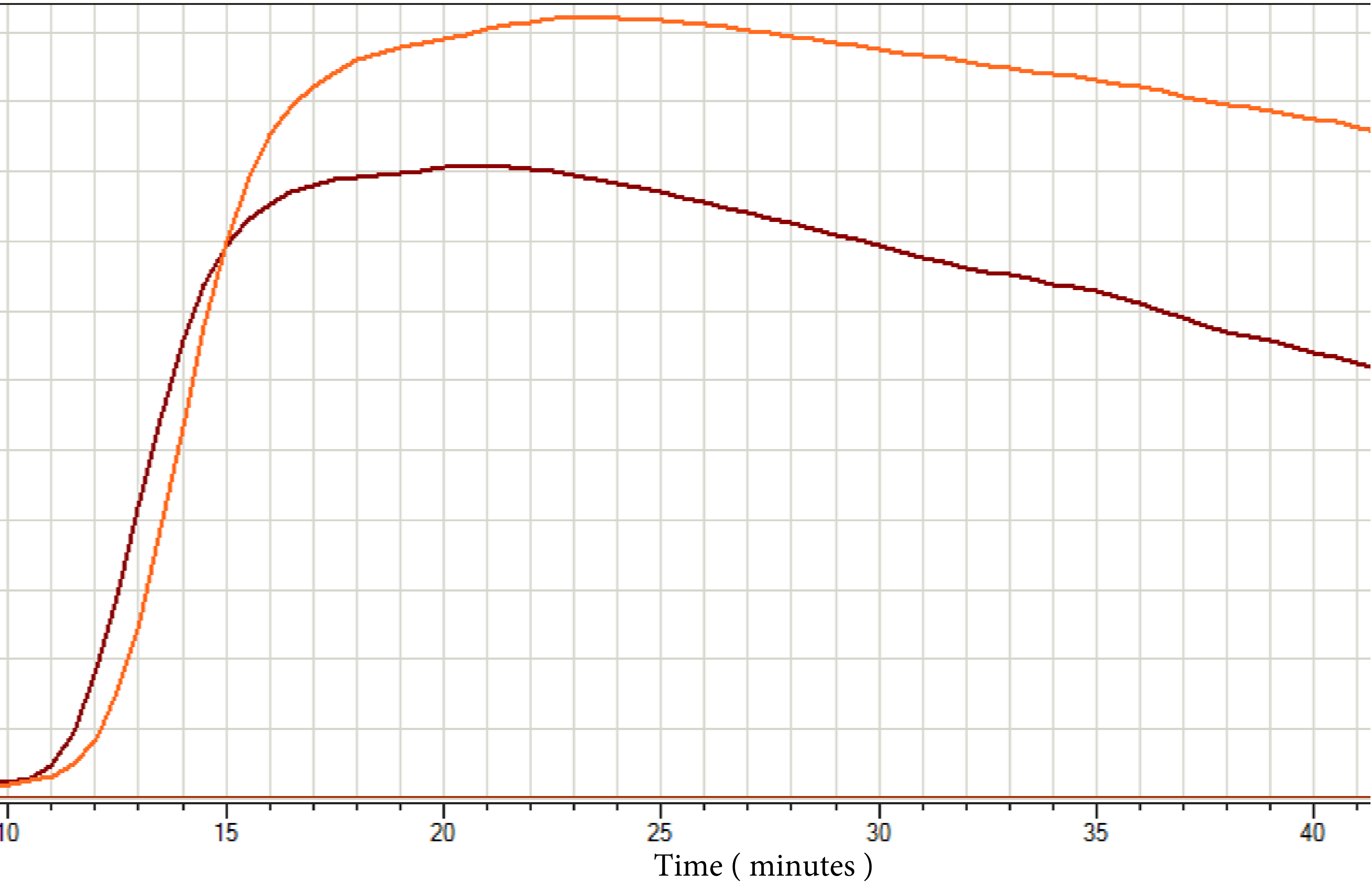

Algorithm Processing Curve

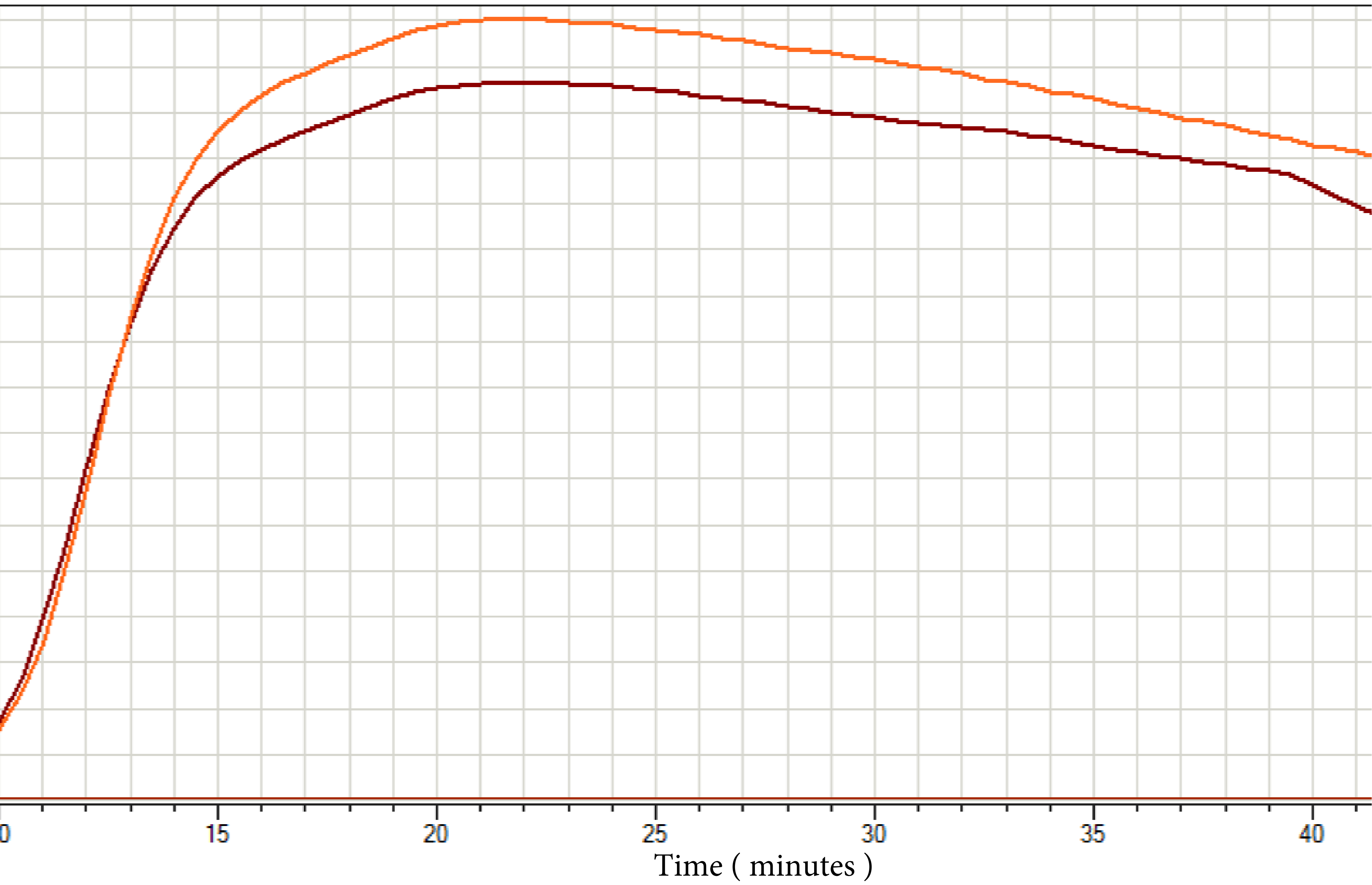

Algorithm Processing Curve

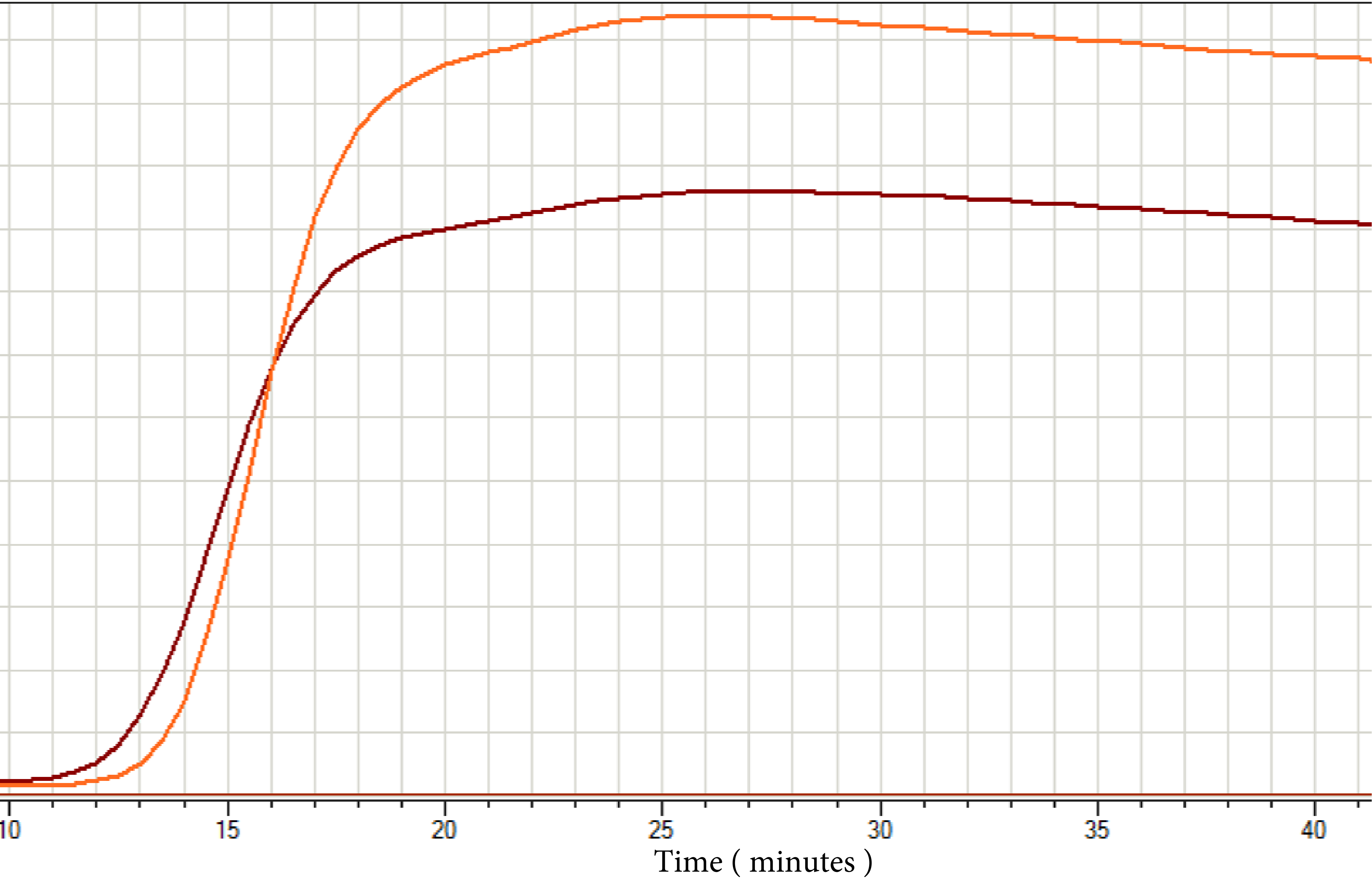

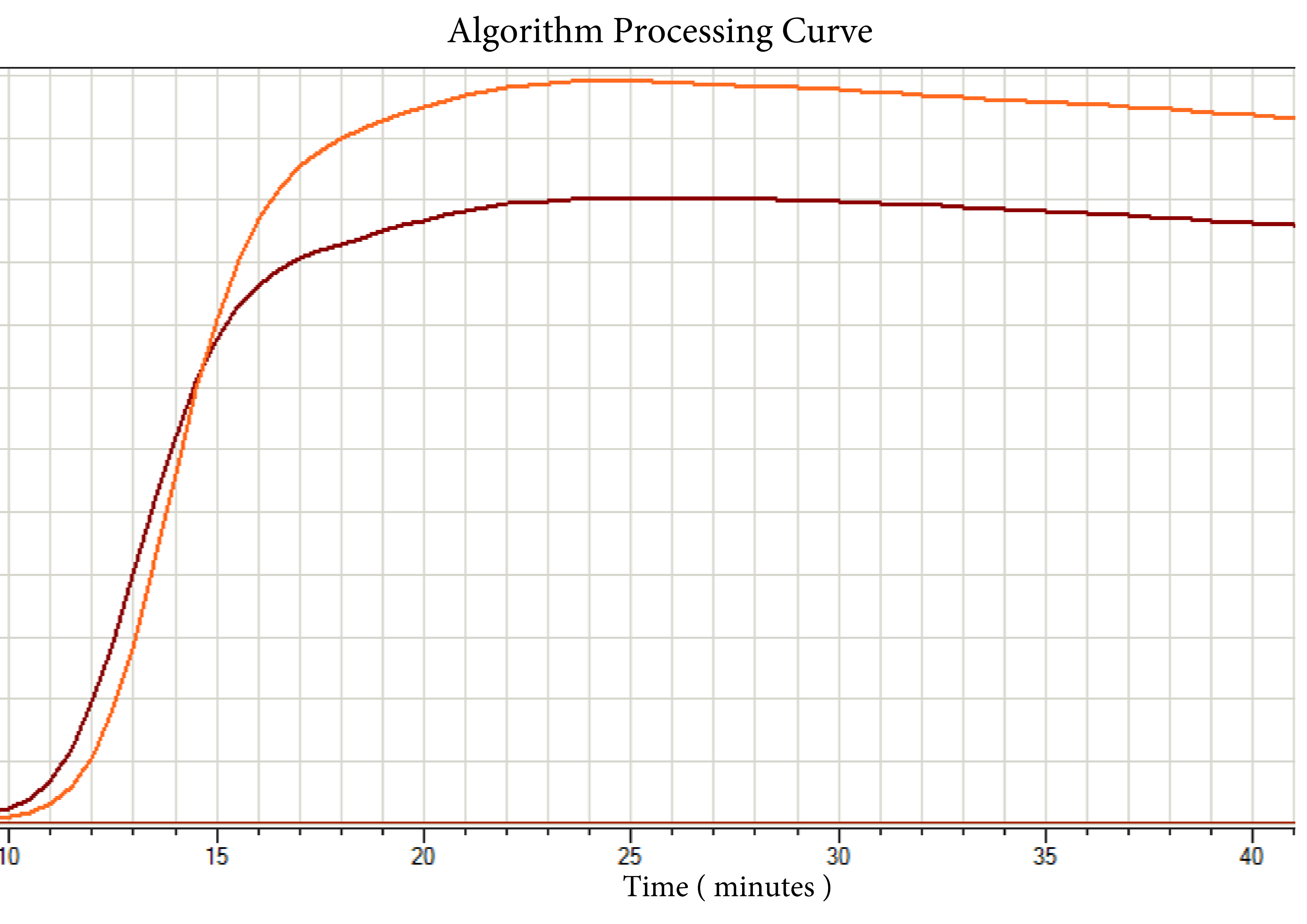
Algorithm Processing Curve
10
15
20
25
30
35
40
Time (minutes)

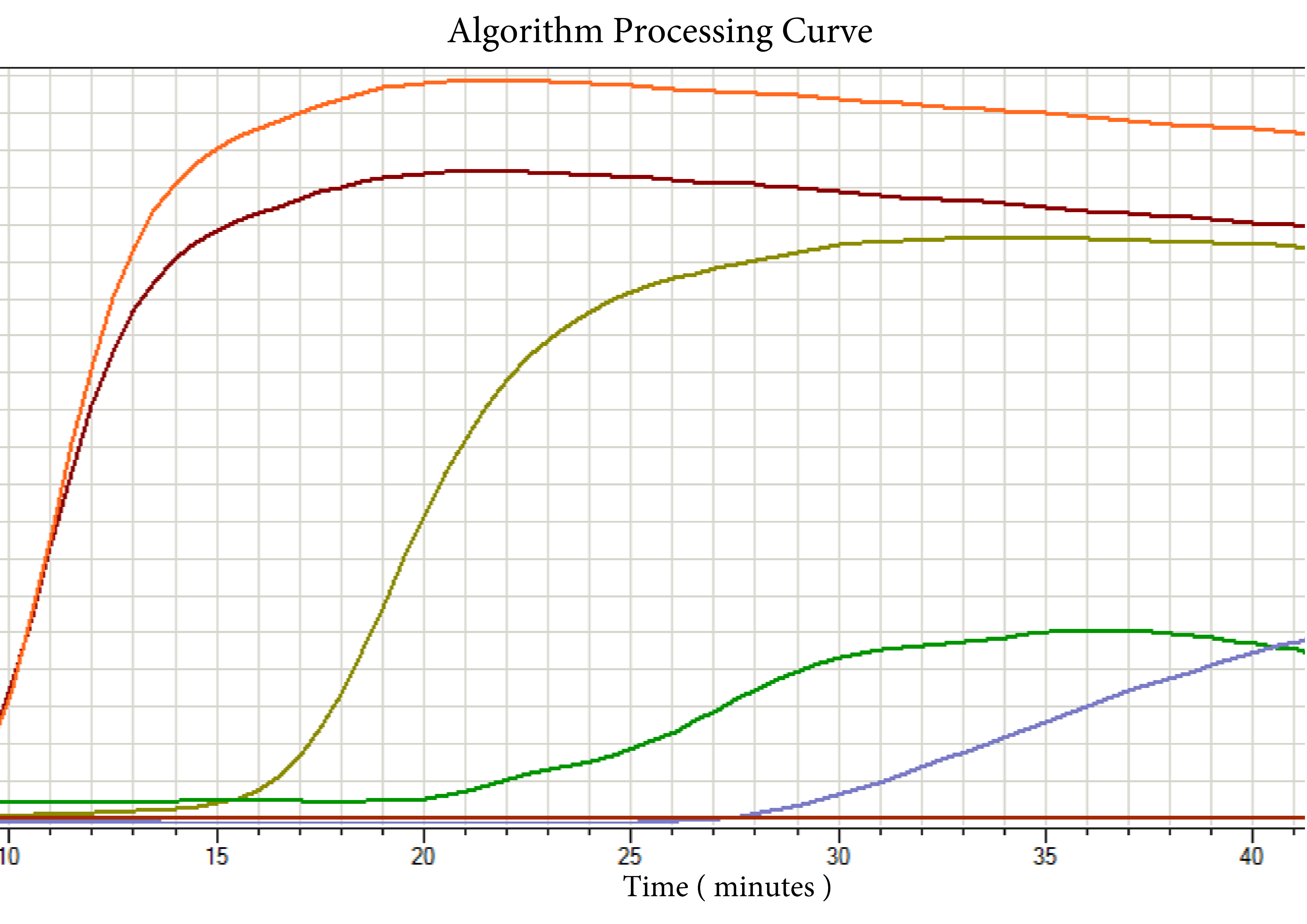
Algorithm Processing Curve
10
15
20
25
30
35
40
Time (minutes)

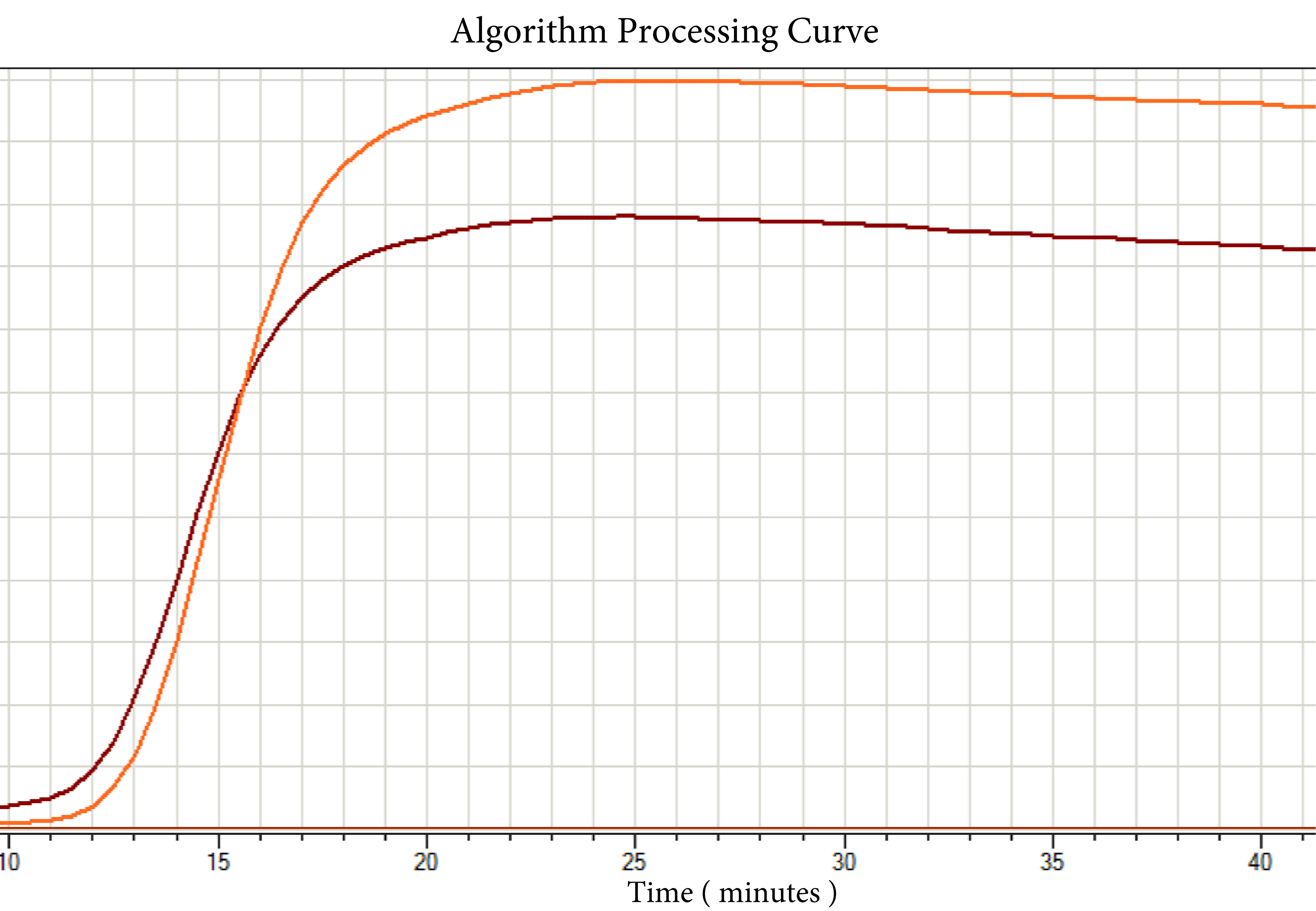
Algorithm Processing Curve
10
15
20
25
30
35
40
Time (minutes)

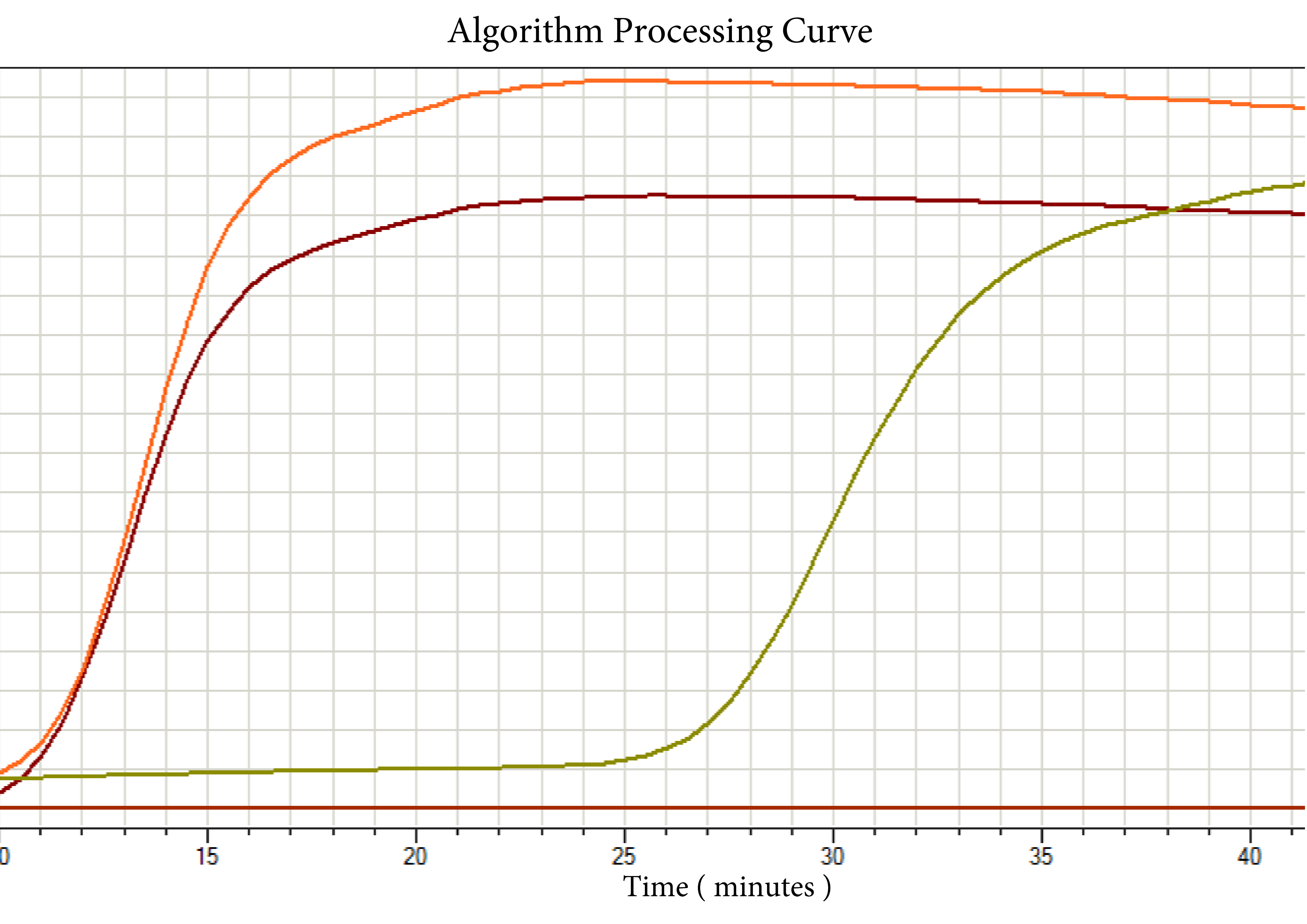

Algorithm Processing Curve
0
15
20
25
30
35
40
Time (minutes)

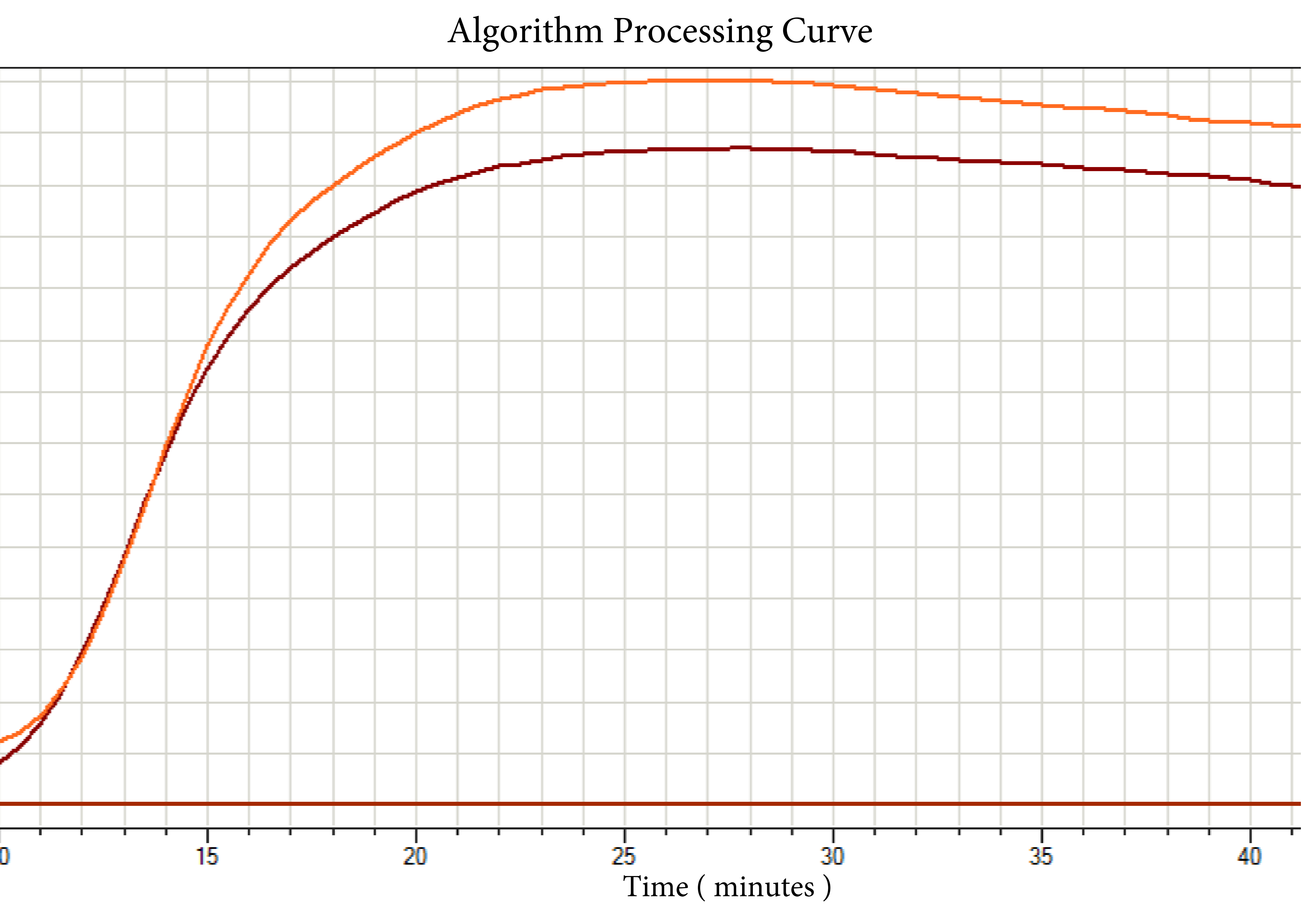

Algorithm Processing Curve
0
15
20
25
30
35
40
Time (minutes)

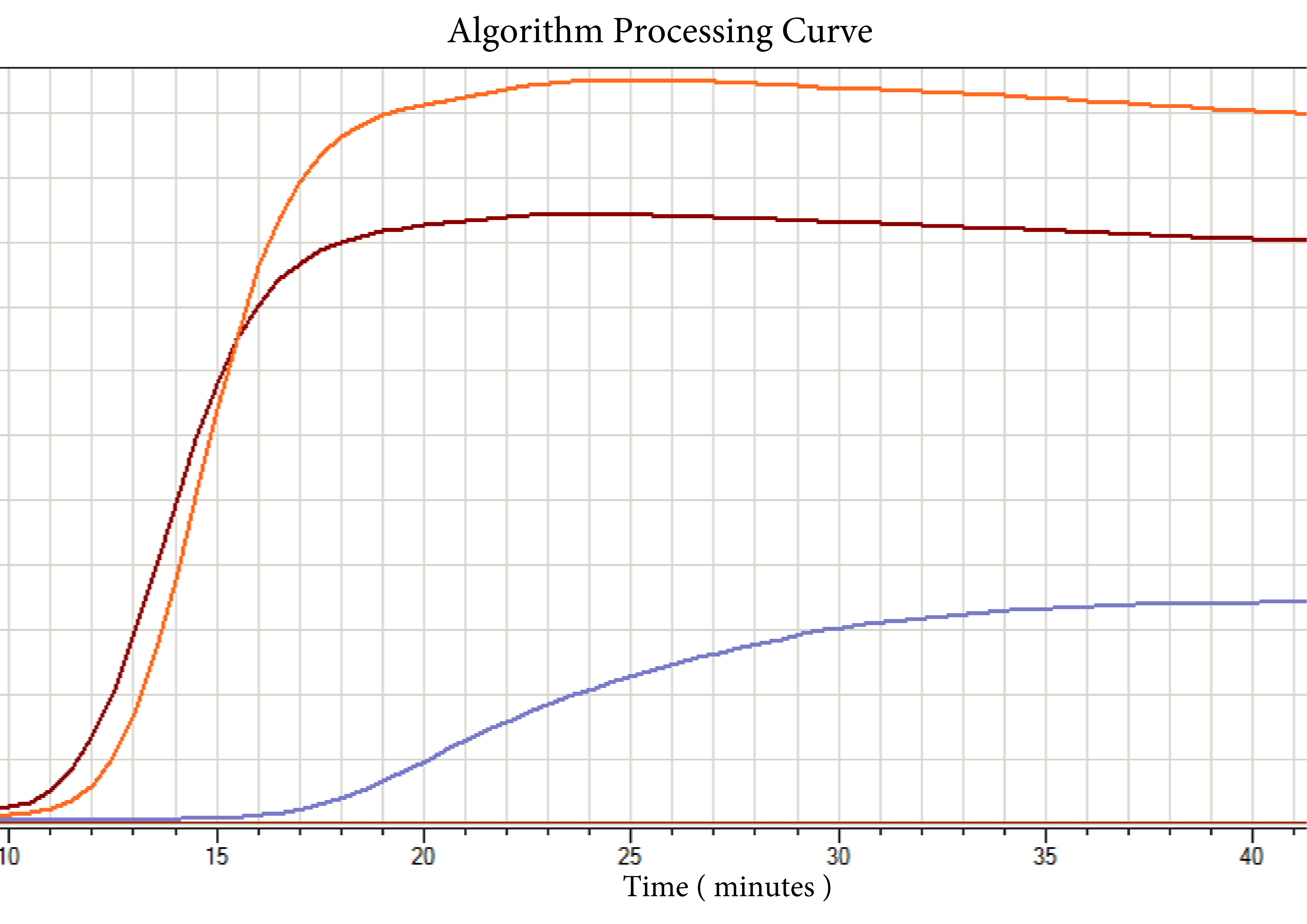
Algorithm Processing Curve
10
15
20
25
30
35
40
Time (minutes)

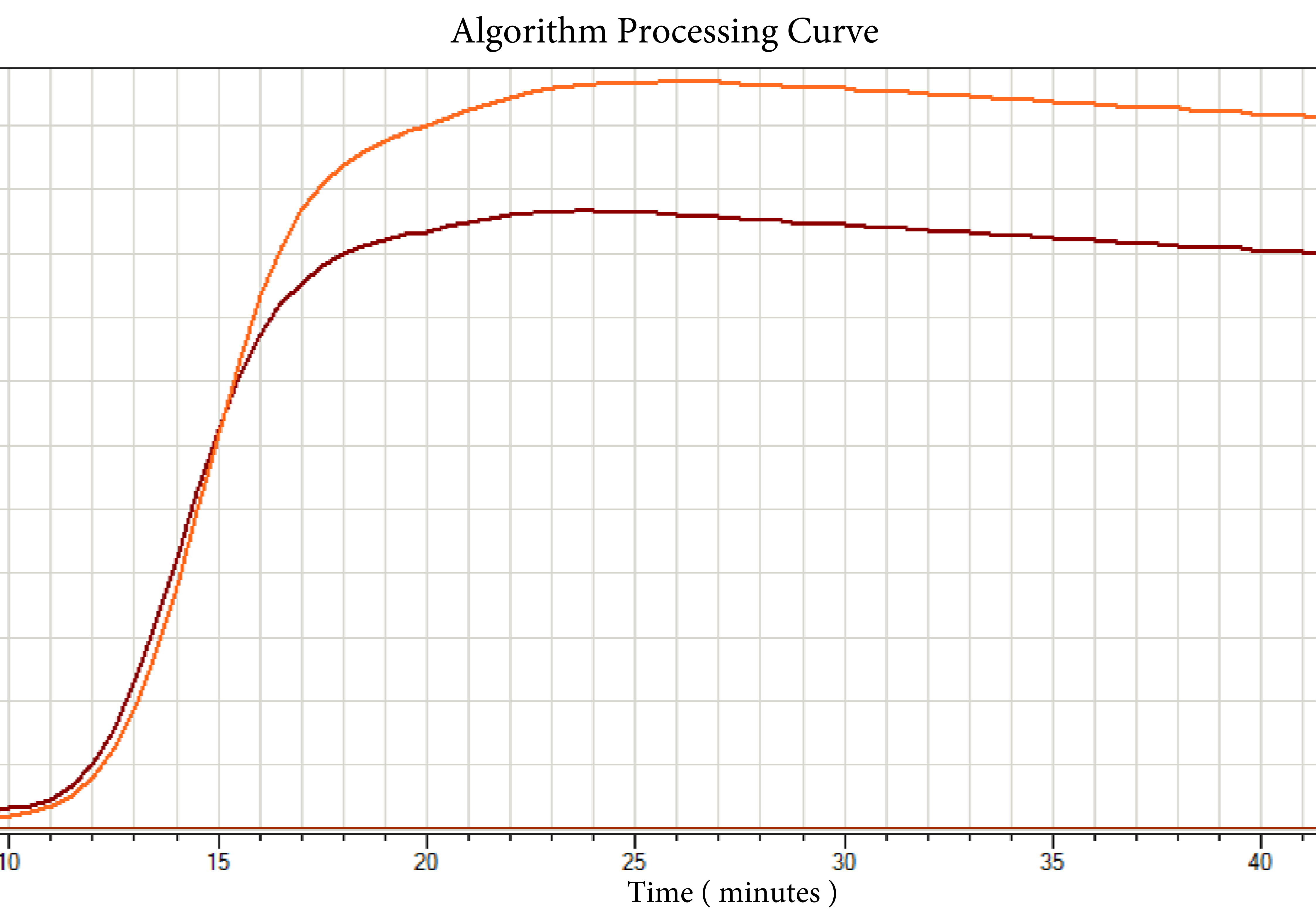

Algorithm Processing Curve
10
15
20
25
30
35
40
Time (minutes)

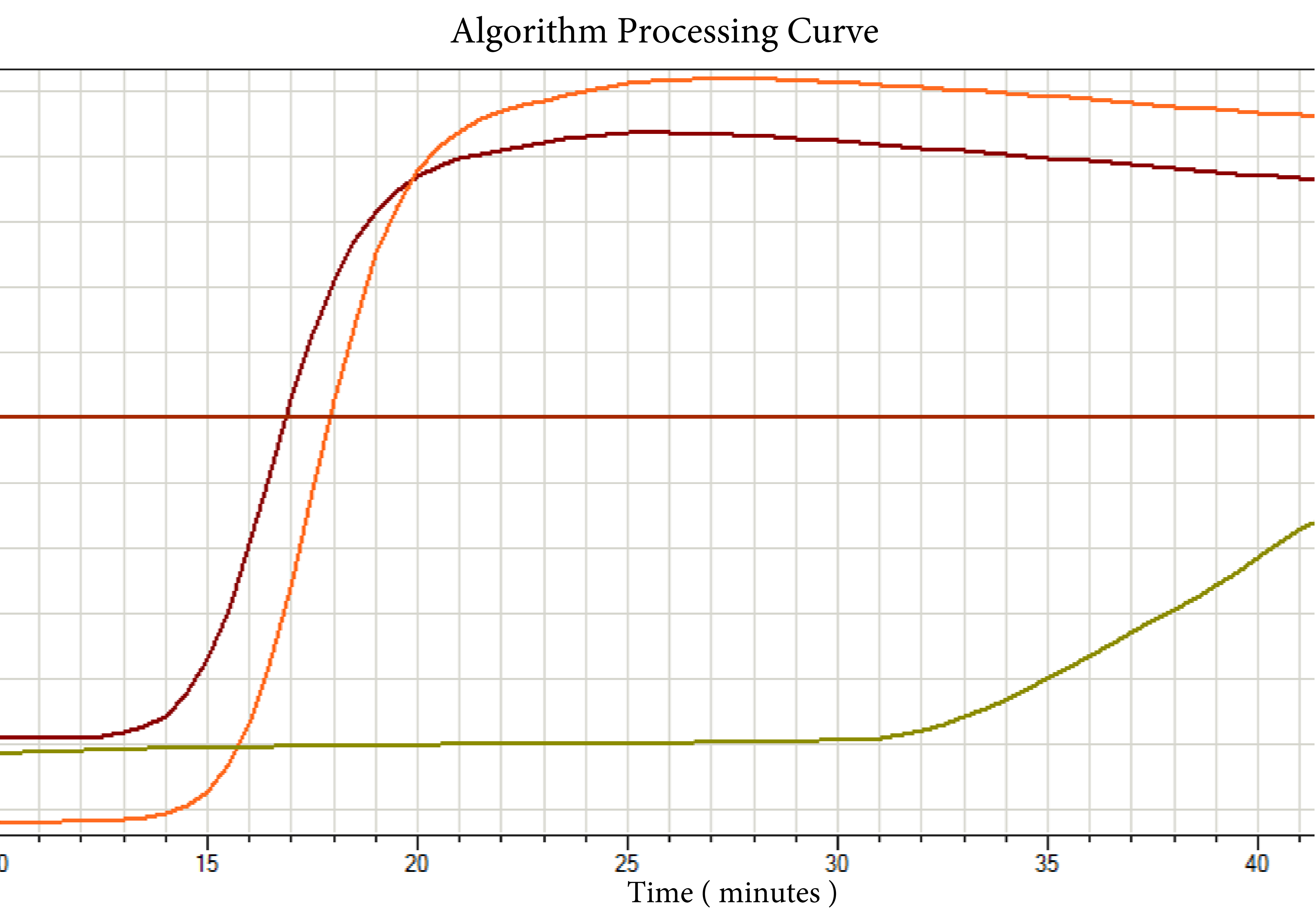

Algorithm Processing Curve
0
15
20
25
30
35
40
Time (minutes)

Real-time Fluorescence Curve

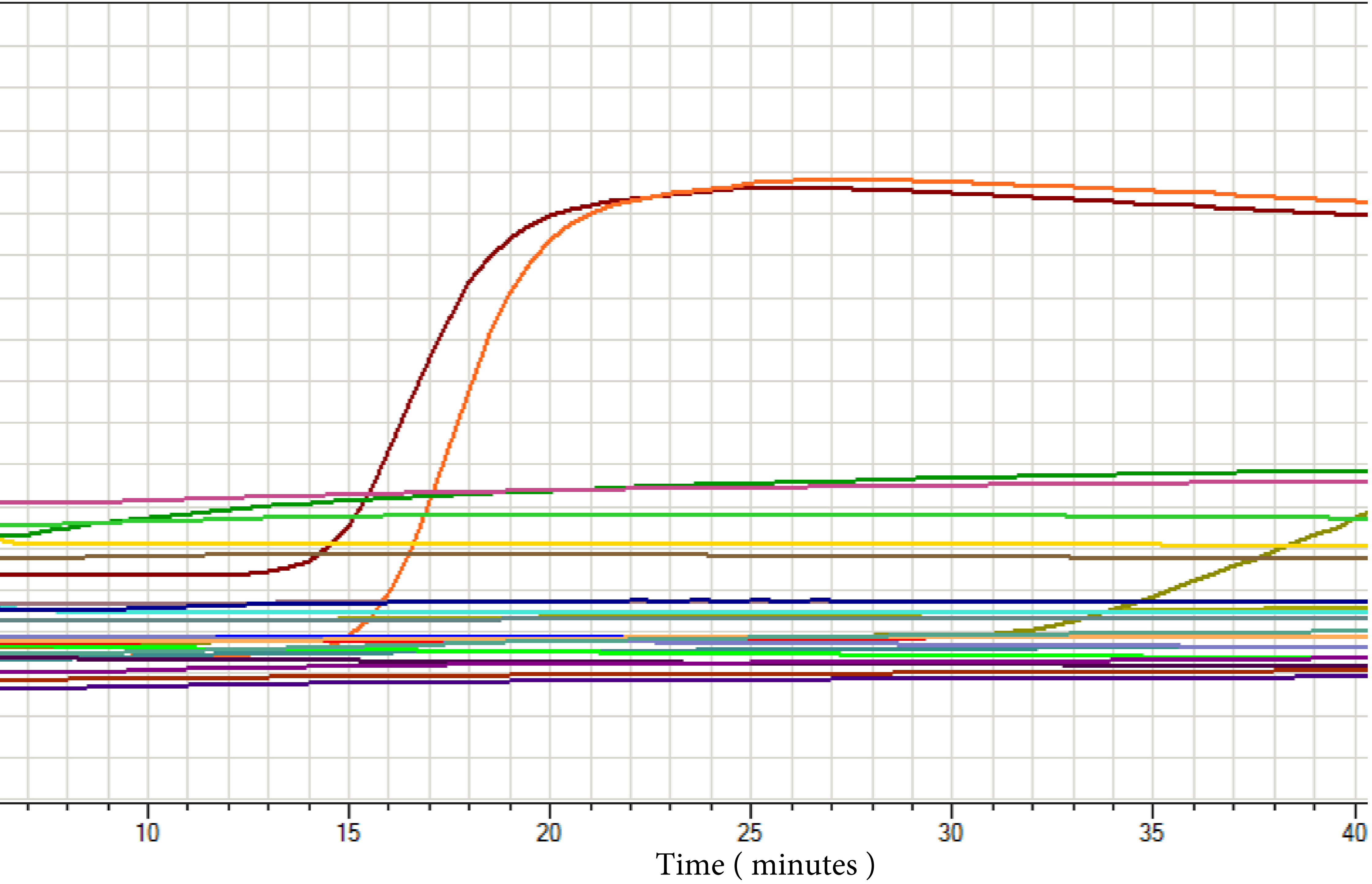

Supplement: Supplementary file 1 — Supplementary Material 1 [file 12879_2024_9212_MOESM1_ESM.pdf]
