# Supplementary material for: Rapid detection of pathogenic fungi from coastal population with respiratory infections using microfluidic chip technology
Source: BMC Infect Dis. 2024 Mar 18;24:326. doi: 10.1186/s12879-024-09212-4 (PMC10949588; doi:10.1186/s12879-024-09212-4)

C. Albicans

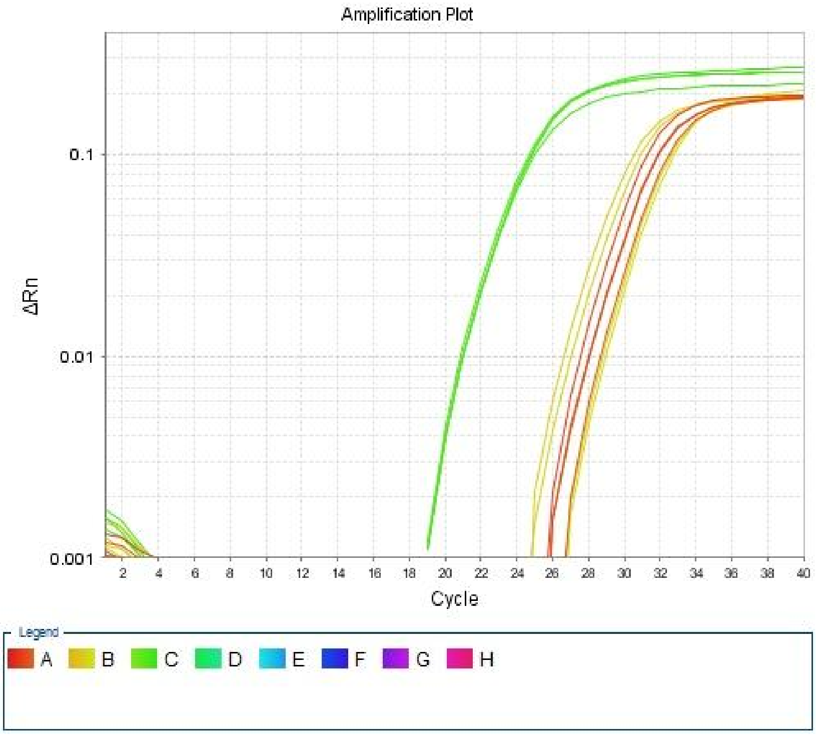
Amplification Plot
ΔRn
0.1
0.01
0.001
Cycle
2 4 6 8 10 12 14 16 18 20 22 24 26 28 30 32 34 36 38 40
Legend
A B C D E F G H

D.

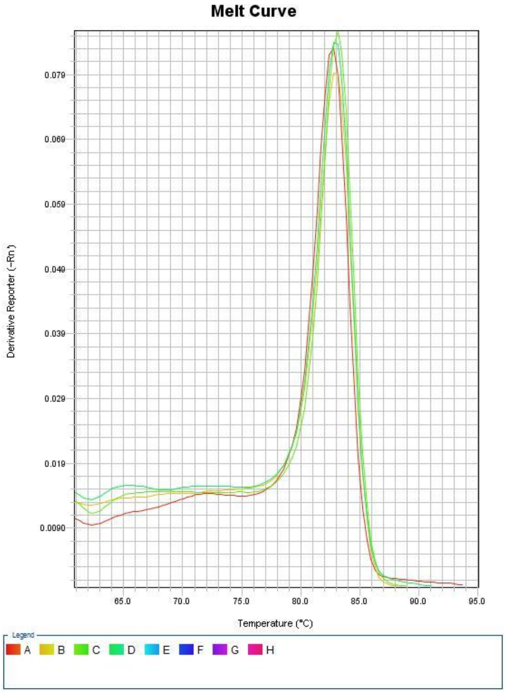
Melt Curve
Derivative Reporter (-Rn)
0.079
0.069
0.059
0.049
0.039
0.029
0.019
0.0090
65.0 70.0 75.0 80.0 85.0 90.0 95.0
Temperature (°C)
Legend
A B C D E F G H

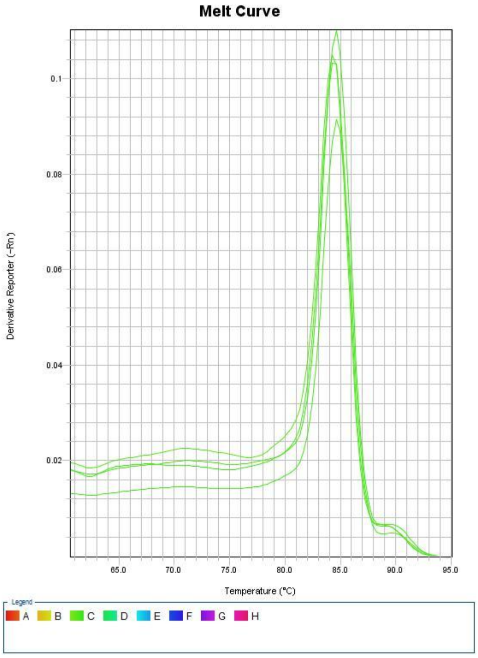
Melt Curve
Derivative Reporter (-Rn)
0.1
0.08
0.06
0.04
0.02
65.0 70.0 75.0 80.0 85.0 90.0 95.0
Temperature (°C)
Legend
A B C D E F G H

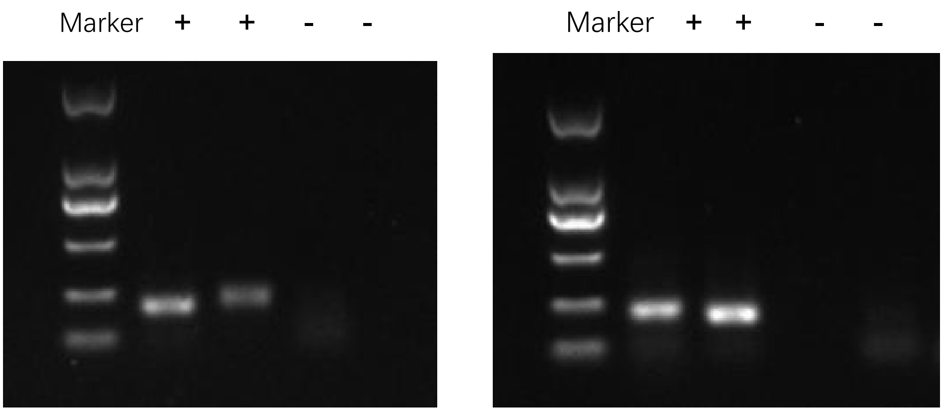

C. tropicalis

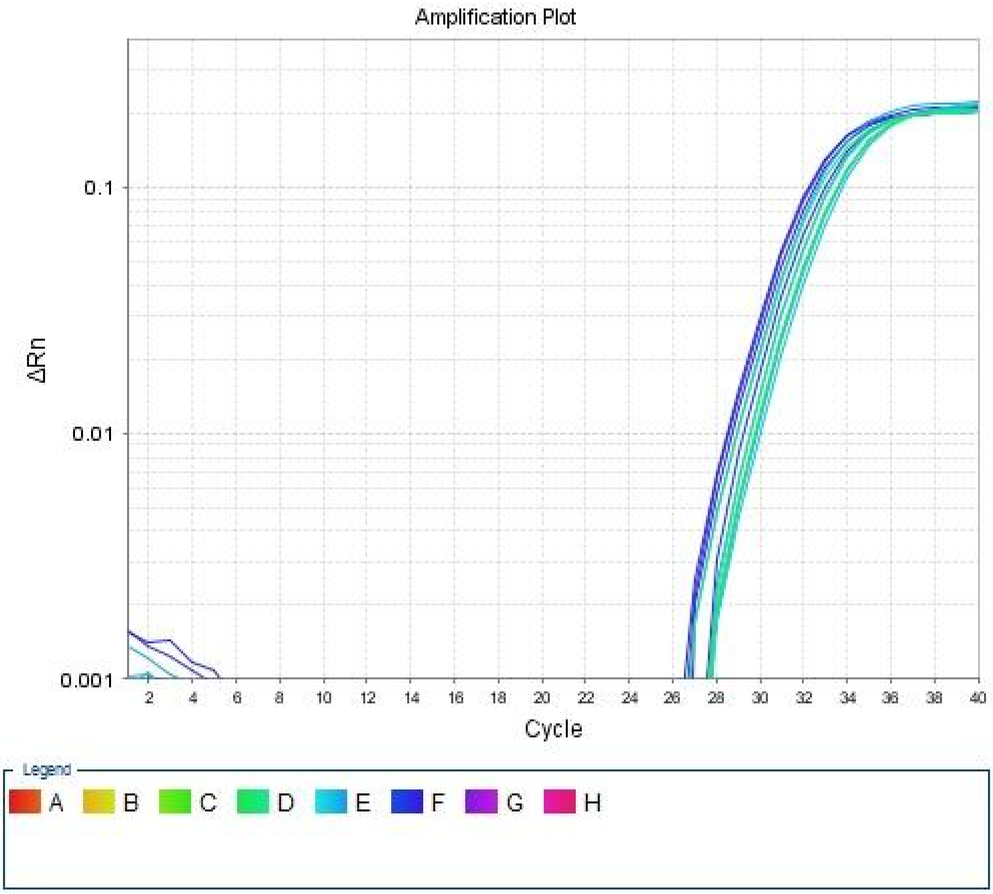

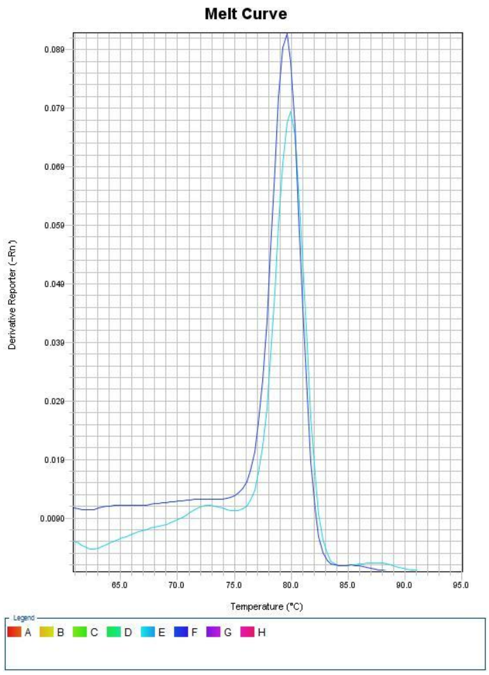
Melt Curve
0.089
0.079
0.069
0.059
0.049
0.039
0.029
0.019
0.0090
Derivative Reporter (-Rn)
65.0
70.0
75.0
80.0
85.0
90.0
95.0
Temperature (°C)
Legend
A B C D E F G H

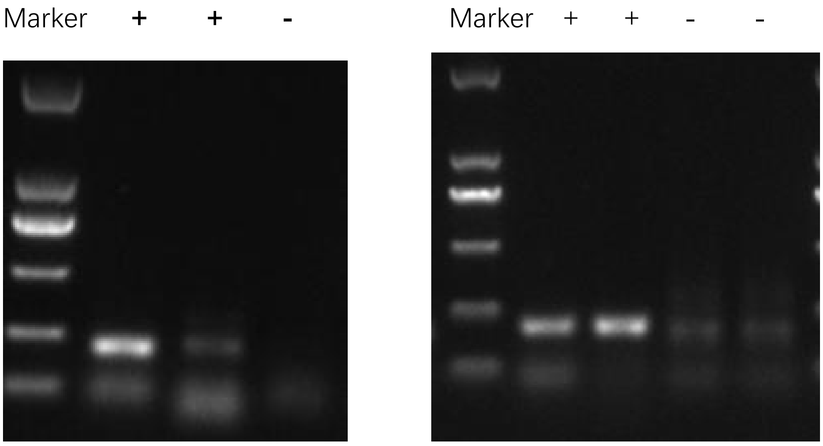
Marker + + -
Marker + + - -

C. glabrata

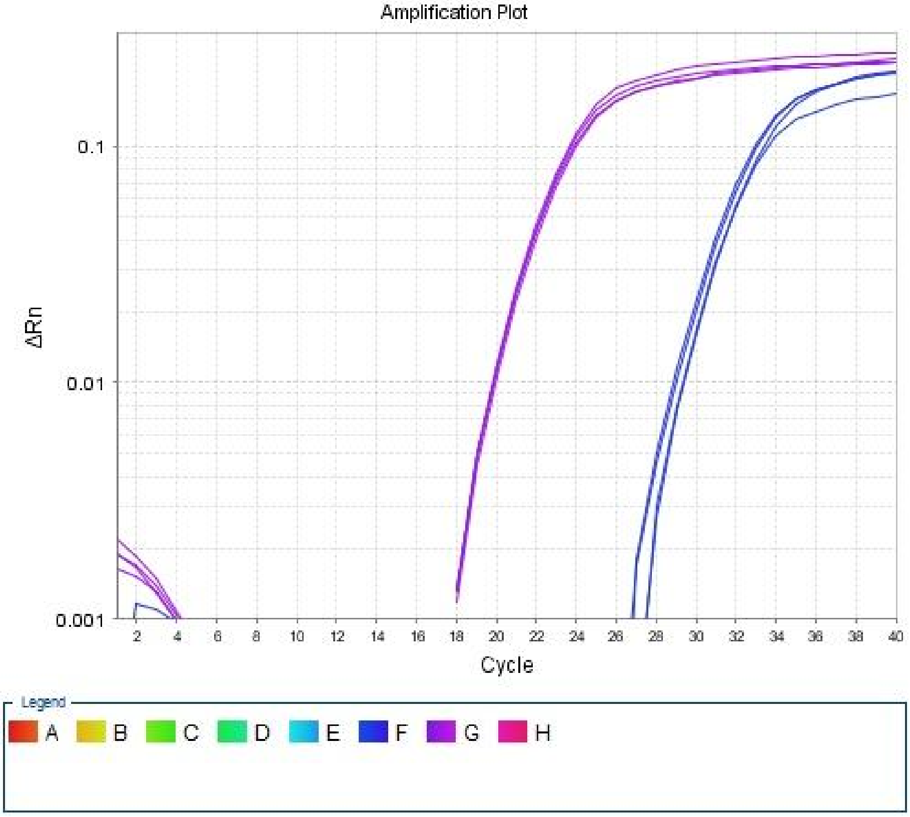
Amplification Plot
ΔRn
0.1
0.01
0.001
2 4 6 8 10 12 14 16 18 20 22 24 26 28 30 32 34 36 38 40
Cycle
Legend
A B C D E F G H

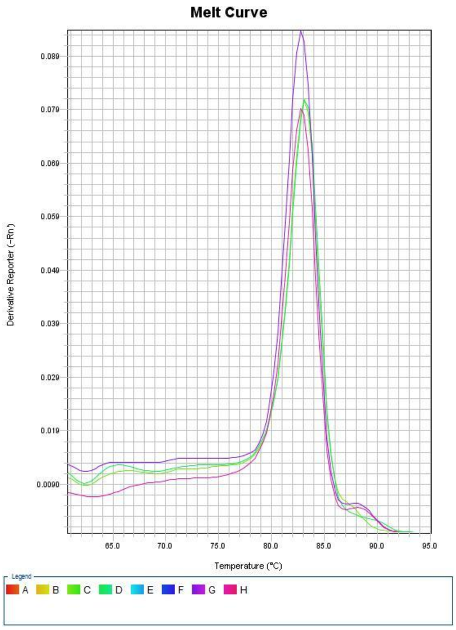
Melt Curve
Derivative Reporter (-Rn)
0.089
0.079
0.069
0.059
0.049
0.039
0.029
0.019
0.0090
65.0 70.0 75.0 80.0 85.0 90.0 95.0
Temperature (°C)
Legend
A B C D E F G H

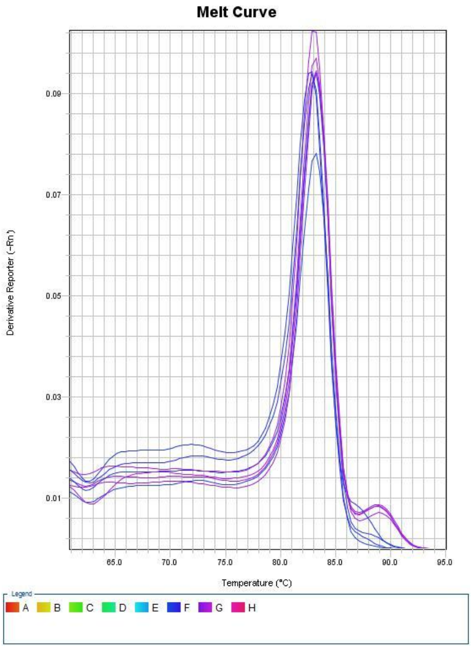
Melt Curve
Derivative Reporter (-Rn)
0.09
0.07
0.05
0.03
0.01
65.0 70.0 75.0 80.0 85.0 90.0 95.0
Temperature (°C)
Legend
A B C D E F G H

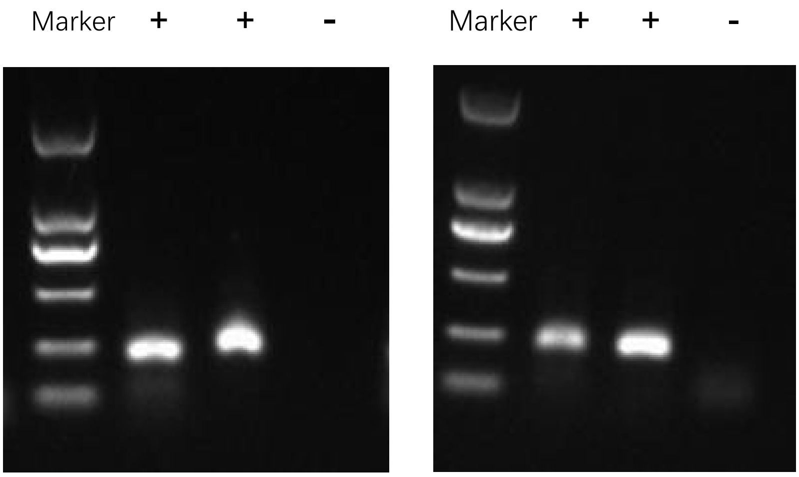

C. parapsilosis

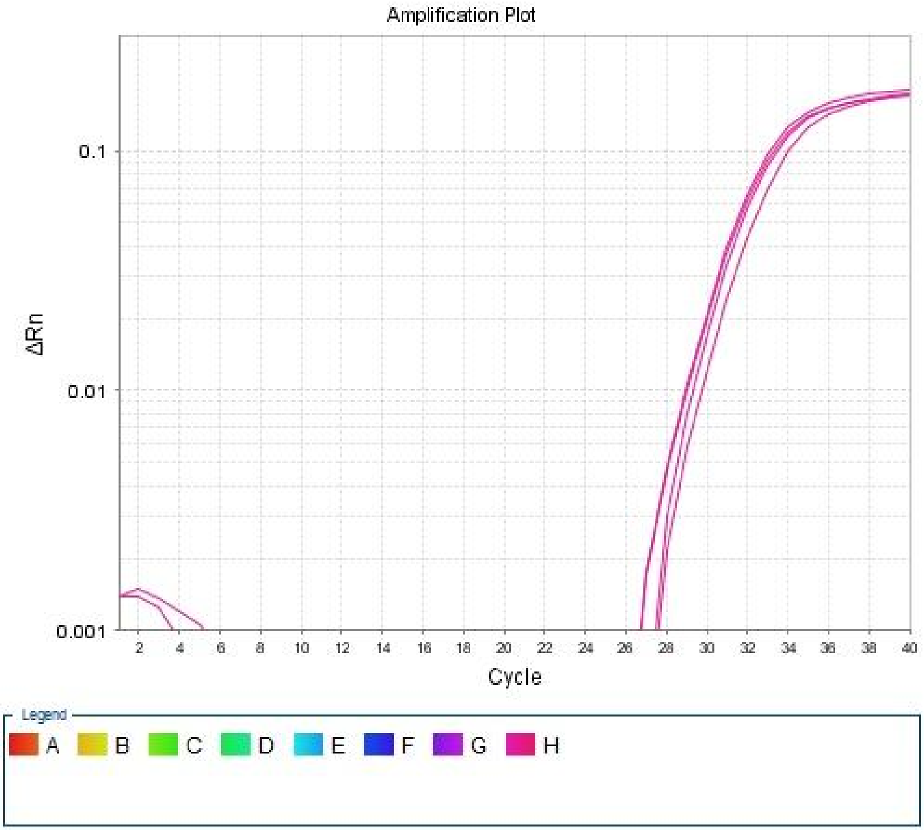

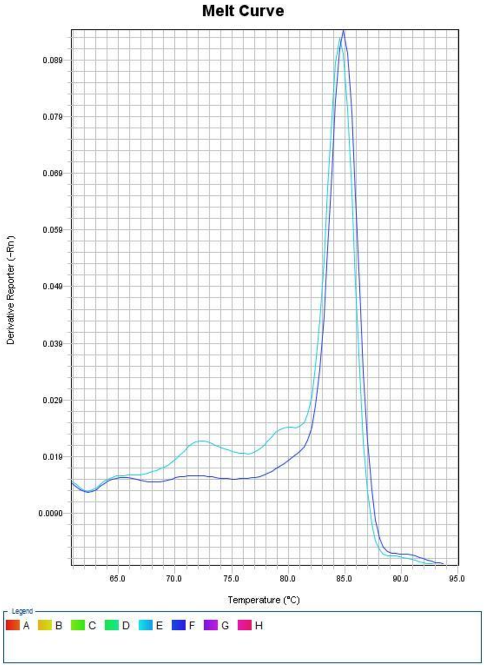

Melt Curve
Derivative Reporter (-Rn)
0.089
0.079
0.069
0.059
0.049
0.039
0.029
0.019
0.0090
65.0
70.0
75.0
80.0
85.0
90.0
95.0
Temperature (°C)
Legend
A B C D E F G H

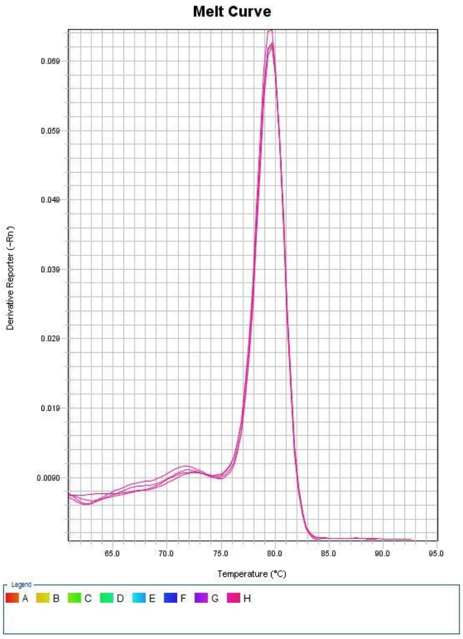

Melt Curve
Derivative Reporter (-Rn)
0.069
0.059
0.049
0.039
0.029
0.019
0.0090
65.0
70.0
75.0
80.0
85.0
90.0
95.0
Temperature (°C)
Legend
A B C D E F G H

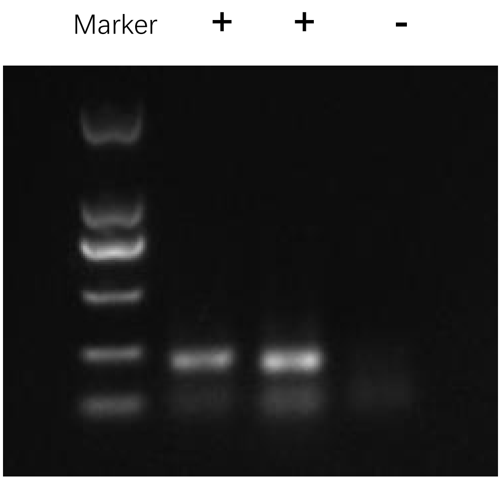

Marker
+
+
-

C. krusei

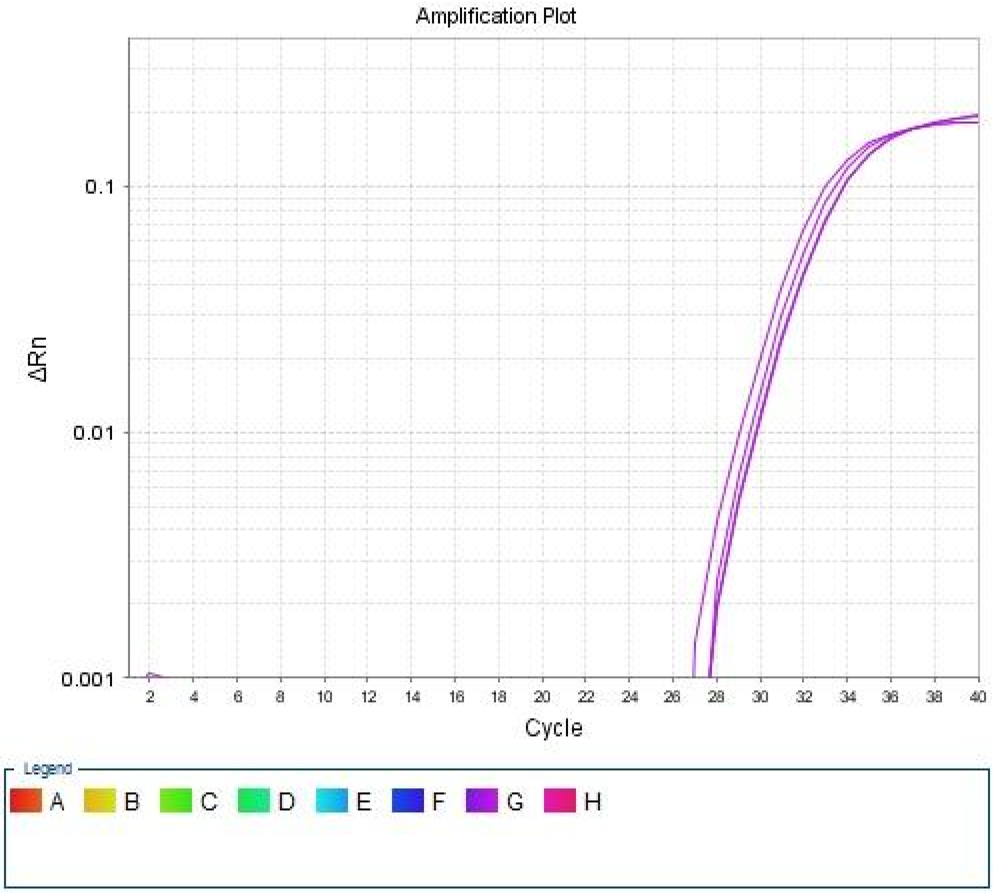
Amplification Plot
ΔRn
0.1
0.01
0.001
2 4 6 8 10 12 14 16 18 20 22 24 26 28 30 32 34 36 38 40
Cycle
Legend
A B C D E F G H

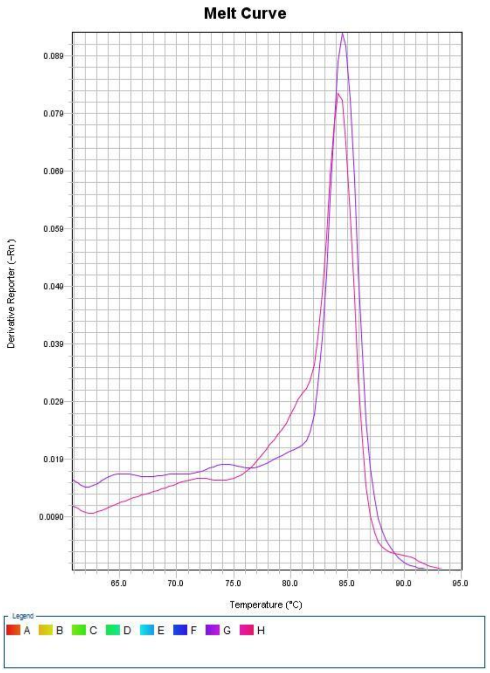
Melt Curve
Derivative Reporter (−Rn')
0.089
0.079
0.069
0.059
0.049
0.039
0.029
0.019
0.0090
65.0 70.0 75.0 80.0 85.0 90.0 95.0
Temperature (°C)
Legend
A B C D E F G H

Marker + + - -

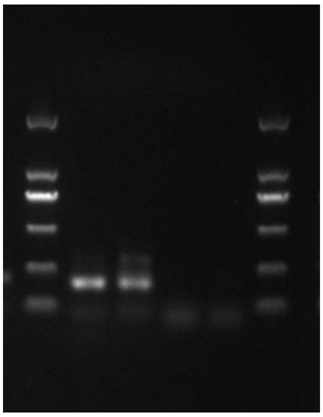

GAPDH

C. albicans

Marker + + - -

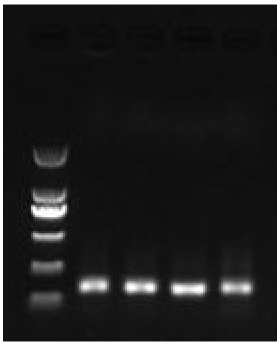

Marker + + - -

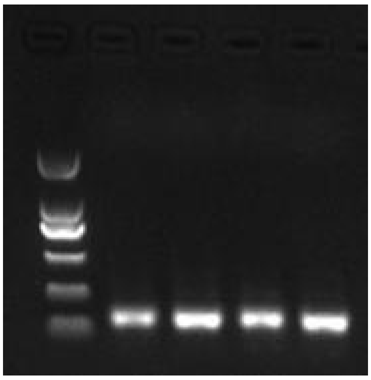

C. tropicalis

Marker + + - -

Marker + + - -

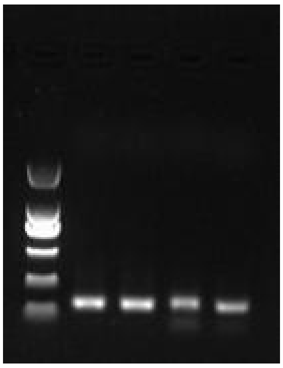

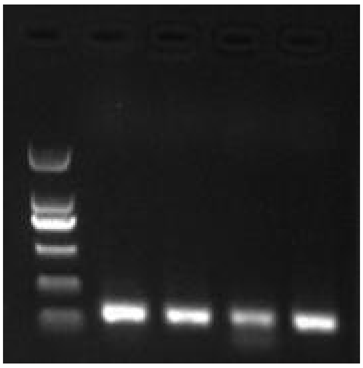

D. Glabrata

Marker + + - -

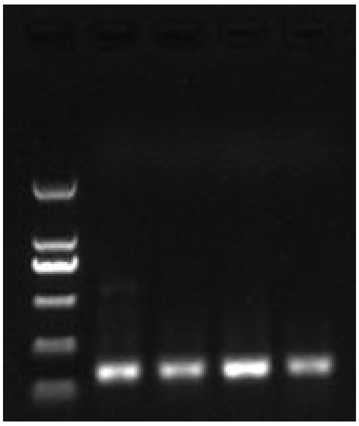

C. Parapsilosis

Marker + + - -

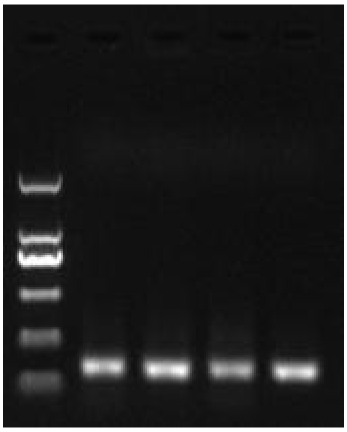

C. krusei

Marker + + - -

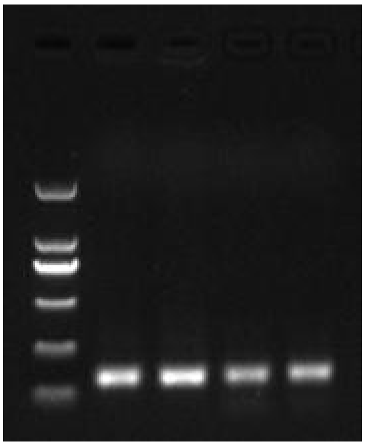

Supplement: Supplementary file 2 — Supplementary Material 2 [file 12879_2024_9212_MOESM2_ESM.pdf]
