# Supplementary material for: Rapid detection of pathogenic fungi from coastal population with respiratory infections using microfluidic chip technology
Source: BMC Infect Dis. 2024 Mar 18;24:326. doi: 10.1186/s12879-024-09212-4 (PMC10949588; doi:10.1186/s12879-024-09212-4)
Supplement: Supplementary file 4 — Supplementary Material 4 [file 12879_2024_9212_MOESM4_ESM.docx]

**Table S2.** Primer sequences used in qPCR validation

| *Candida* spp. | Primer sequences |
| --- | --- |
| *C. albicans* | Forward: 5’- CGATACGTAATATGAATTGCAGAT -3’ |
|  | Reverse: 5’- CCATTGTCAAAGCGATCC -3’ |
| *C. tropicalis* | Forward: 5’- GAGCGTCATTTCTCCCTC -3’ |
|  | Reverse: 5’- GCTTATTGATATGCTTAAGTTCAG -3’ |
| *C. glabrata* | Forward: 5’- TCTCGCATCGATGAAGAAC -3’ |
|  | Reverse: 5’- CACATACTGATATGGCCTACA -3’ |
| *C. parapsilosis* | Forward: 5’- GGTGAAGATTCTATTACTGATTGG -3’ |
|  | Reverse: 5’- CTTTTACCCCATTGCACAAT -3’ |
| *C. krusei* | Forward: 5’- GGATCTCTTGGTTCTCGC -3’ |
|  | Reverse: 5’- GCTTCGCTCCCTTTCAG -3’ |
